# Proteome Profiling of Canine Epididymal Fluid: In Search of Protein Markers of Epididymal Sperm Motility

**DOI:** 10.3390/ijms241914790

**Published:** 2023-09-30

**Authors:** Aleksandra W. Cichowska, Jerzy Wisniewski, Mariusz A. Bromke, Beata Olejnik, Marzena Mogielnicka-Brzozowska

**Affiliations:** 1 Department of Animal Biochemistry and Biotechnology, University of Warmia and Mazury in Olsztyn, Oczapowskiego 5, 10-719 Olsztyn, Poland; 2 Department of Biochemistry, Molecular Biology and Biotechnology, Wroclaw University of Science and Technology, Wyspianskiego 27, 50-370 Wroclaw, Poland; 3 Department of Biochemistry and Immunochemistry, Wroclaw Medical University, Chalubinskiego 10, 50-368 Wroclaw, Poland

**Keywords:** canine, epididymal fluid, epididymal spermatozoa, motility, proteome

## Abstract

Sperm maturation in the epididymis is based on interactions with proteins from epididymal fluid (EF). The aim of the study was to profile canine EF proteome and investigate correlations between EF protein content and epididymal spermatozoa (ES) motion parameters. Twenty-three male dogs were divided into two groups: good sperm motility (GSM) and poor sperm motility (PSM). The total motility and progressive motility differed significantly (*p* = 0.031; *p* < 0.001, respectively) between the GSM group and the PSM group. The semen samples were centrifuged to separate the EF apart from the ES. The canine EF proteins were analyzed using nano-liquid chromatography, which was coupled with quadrupole time-of-flight mass spectrometry (NanoUPLC-Q-TOF/MS) and bioinformatic tools for the first time. A total of 915 proteins were identified (GSM—506; PSM—409, respectively). UniProt identification resulted in six unique proteins (UPs) in the GSM group of dogs and four UPs in the PSM group. A semi-quantitative analysis showed a higher abundance (*p* < 0.05) of four differentially expressed proteins in the GSM group (ALB, CRISP2, LCNL1, PTGDS). Motility-dependent variations were detected in the EF proteome and were related to important metabolic pathways, which might suggest that several proteins could be potential ES motility biomarkers.

## 1. Introduction

The essential role of the epididymis in male fertility has been described extensively [1,2,3]. As sperm passes through the epididymis, it undergoes a maturation process that is characterized by morphological and physiological modifications necessary to obtain full capacity for fertilization. These modifications comprise changes in the biochemical composition of plasmalemma, including the addition, removal, or transformation of proteins and lipids, as well as final chromatin condensation and the acquisition of functional features, such as progressive motility [4,5]. This functional epididymal sperm (ES) maturation occurs in response to modifications in the epididymal fluid (EF) microenvironment [6]. The ES surface during transit along the epididymis is directly exposed to an epididymal milieu rich in numerous proteins, which may interact with the sperm in a specific manner [4,7]. This may have an impact on the functional features of the male reproductive cells [8]. Epididymal fluid proteins are thought to have a significant role in the subsequent steps leading to acquiring the ES functional capability for fertilization [9].

The mammalian spermatozoa leaving the testicles are immobile. In order to reach the oocyte, the sperm must be able to move progressively forward. Therefore, motility is a significant functional sperm parameter in both natural and assisted reproduction. Of all known semen quality parameters, sperm motility is considered to be a highly predictive marker of male fertility [10]. The sperm acquire this property gradually while they pass through the epididymal tract. Considering that approximately 17.5% of couples worldwide face fertility problems [11], as well as the development of animal husbandry and the breeders’ attitude towards reproductive success, it seems advisable to understand the mechanisms of sperm motility regulation by molecules present in EF.

A complete profiling of proteins present in a tissue or a cell is defined as proteomics [12]. Currently, this field of science is commonly used and has promising potential in animal reproduction research. The detection and identification of proteins found in EF may reveal unknown interactions between the components of these fluids and the sperm, which may affect the functional features of male gametes. Identification of the sperm motility-modulating proteins may be used in biomedical applications in strategies of infertility treatment, semen assessment, and conservation centers, both in animals and humans [13]. The development of a procedure for the identification of protein markers, which proves the quality of semen and its usefulness for reproduction on a dog model, would allow for the expansion of knowledge, not only in the aspect of dog breeding and preservation of endangered canine species but also in the field of human reproduction.

Taking into consideration that the epididymis provides a unique environment in which sperm acquire the fertilizing ability [7], it was decided to: (i) Provide profiling of the canine EF proteome; (ii) Investigate correlations between cauda EF proteins and selected ES motion parameters in the search for protein markers of ES motility.

## 2. Results

### 2.1. Epididymal Spermatozoa Quality Assessment

The concentration of ES in the studied groups of dogs ranged as follows: GSM—from 21.4 to 56.4 × 10^8^ spermatozoa/mL (34.74 ± 2.5 × 10^8^ spermatozoa/mL, mean ± SEM) and PSM—from 17.1 to 40.1 × 10^8^ spermatozoa/mL (33.17 ± 2.3 × 10^8^ spermatozoa/mL, mean ± SEM). There were no statistically significant differences (*p* < 0.05) between GSM and PSM groups in ES concentration.

The TMOT and PMOT differed significantly between the GSM group and PSM group (*p* = 0.031; *p* < 0.001, respectively). The obtained results showed significant differences in the VSL, VCL, ALH, and BCF values (*p* = 0.030; *p* = 0.011; *p* = 0.003; *p* < 0.001, respectively) between the studied groups. Moreover, significant differences in the percentage of STR (*p* < 0.001) and LIN (*p* < 0.001) between the studied groups of dogs were observed (Table 1). There were no statistically significant differences (*p* < 0.05) between GSM and PSM groups in VAP values or in rapid, medium, slow, or static motion parameters (Table 1).

### 2.2. Epididymal Fluid Protein Mass Spectrometry Analysis

#### 2.2.1. Qualitative Analysis

A total of 915 proteins were identified in both groups of dogs (GSM—506; PSM—409) by nanoUPLC-Q-TOF/MS (Supplementary Tables S1 and S2).

**Figure 1:**
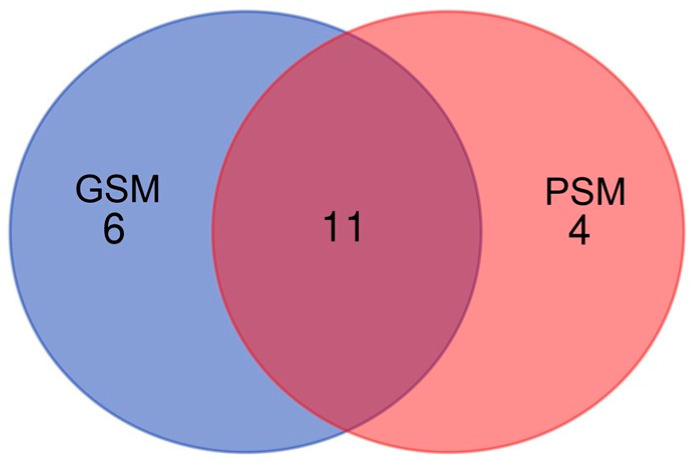
A Venn diagram showing the number of UPs and common proteins that were identified in the cauda EF of dogs (*Canis lupus familiaris*) divided into two groups according to values of ES progressive motility: GSM and PSM.

UniProt database-supported identification resulted in six unique proteins (UPs) that were identified in the GSM group of dogs and four UPs that were identified in the PSM group. Eleven proteins were common for GSM and PSM groups. UPs and common proteins were considered to be statistically significant (when present in 50% + 1 animals). The polypeptides, which were identified in the GSM group as UPs, were: Actin, cytoplasmic 1 (ACTB), Alkaline phosphatase (ALPL), Caspase recruitment domain family member 6 (CARD6), Clusterin (CLU), Polypeptide N-acetylgalactosaminyltransferase (GALNT6), and Olfactomedin 4 (OLFM4) (Figure 1). Four polypeptides were identified in the PSM group as UPs: Boule homolog, RNA-binding protein (BOLL), KRAB domain-containing protein (LOC606925), Cystatin domain-containing protein (LOC607874), and WAP domain-containing protein (N/A/WAPdcp) (Figure 1).

Protein identification resulted in 11 proteins common for both groups, GSM and PSM: Alpha-fetoprotein (AFP), Albumin (ALB), Carboxylesterase 5A (CES5A), Cysteine-rich secretory protein 2 (CRISP2), Epididymal sperm-binding protein 1 (ELSPBP1), Epididymal secretory glutathione peroxidase (GPX5), Lipocln_cytosolic_FA-bd_dom domain-containing protein (LCNL1), Lactotransferrin (LTF), NPC intracellular cholesterol transporter 2 (NPC2), Plastin 3 (PLS3), and Prostaglandin-H2 D-isomerase (PTGDS) (Figure 1).

#### 2.2.2. Semi-Quantitative Analysis

**Figure 2:**
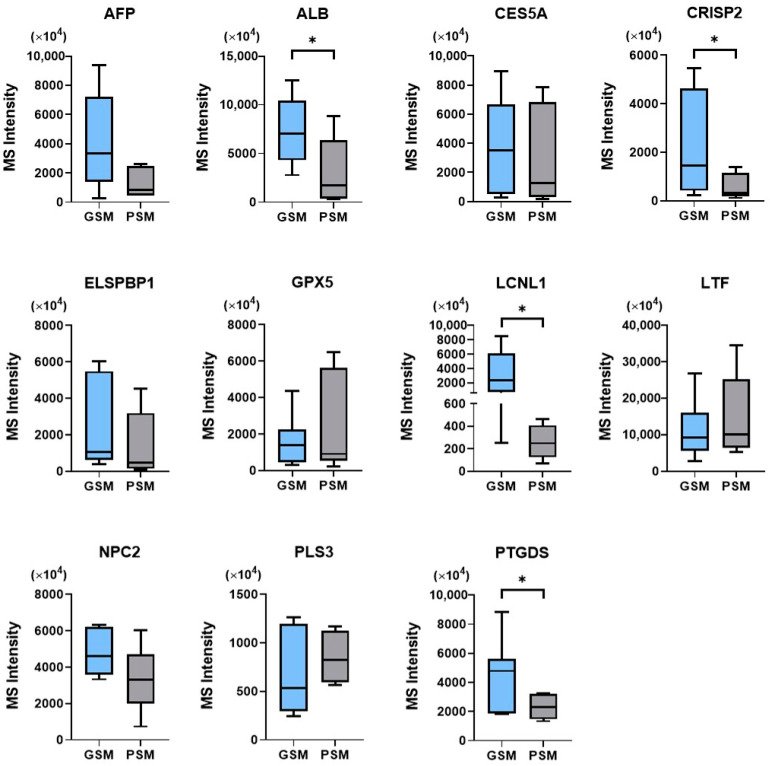
Box plots representing the abundance (MS intensity) distribution of common proteins in cauda EF of dogs (*Canis lupus familiaris*) divided into two groups according to values of ES progressive motility: GSM and PSM. (*) indicates significant differences between GSM and PSM groups (*p* < 0.05). AFP—Alpha-fetoprotein, ALB—Albumin, CES5A—Carboxylesterase 5A, CRISP2—Cysteine-rich secretory protein 2, ELSPBP1—Epididymal sperm-binding protein 1, GPX5—Epididymal secretory glutathione peroxidase, LCNL1—Lipocln_cytosolic_FA-bd_dom domain-containing protein, LTF—Lactotransferrin, NPC2—NPC intracellular cholesterol transporter 2, PLS3—Plastin 3, PTGDS—Prostaglandin-H2 D-isomerase.

A semi-quantitative analysis of protein abundance, based on MS intensity measurement, was also included in the study. When comparing the MS intensity of proteins common for both studied groups (GSM and PSM), statistically significant differences (*p* < 0.05) were found for four proteins. For all of the four proteins, MS intensities were higher in the GSM group (ALB: 7314 ± 1518 × 10^4^; CRISP2: 2235 ± 882.9 × 10^4^; LCNL1 3270 ± 1268 × 10^4^; PTGDS: 4646 ± 904.7 × 10^4^; mean ± SEM) when compared with the PSM group (ALB: 3030 ± 1594 × 10^4^; CRISP2: 628 ± 202.6 × 10^4^; LCNL1: 260.8 ± 60.65 × 10^4^; PTGDS: 2323 ± 280.9 × 10^4^; mean ± SEM) (Figure 2).

No significant differences (*p* < 0.05) between GSM and PSM groups were found when comparing the MS intensity of AFP, CES5A, ELSPBP1, GPX5, LTF, NPC2, and PLS3 (Figure 2).

### 2.3. Bioinformatic Analysis

#### 2.3.1. Gene Ontology and ToppCluster Analysis

A GO analysis of proteins found in GSM and PSM groups showed similar main molecular functions, biological processes, and protein classes.

**Figure 3:**
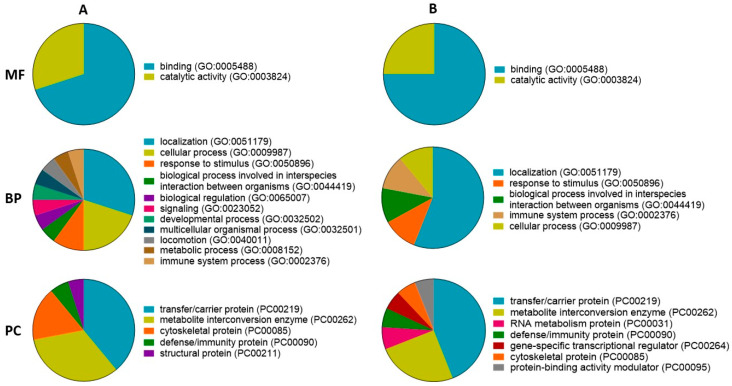
GO enrichment of proteins presented in cauda EF of dogs (*Canis lupus familiaris*) divided into two groups according to values of ES progressive motility: GSM (**A**) and PSM (**B**). The following GO terms were distinguished: molecular function (MF), biological process (BP), and protein class (PC).

Based on GO enrichment, the most dominant molecular functions of the proteins in the GSM group were binding (63%) and catalytic activity (37%) (Figure 3A: MF). In addition, a GO analysis of the proteins in the PSM group showed that dominant molecular function terms were binding (75%) and catalytic activity (25%) (Figure 3B: MF). For the proteins found in the GSM group, the dominant biological processes were localization (30%), cellular process (20%), and a response to stimulus (10%) (Figure 3A: BP). For the proteins found in the PSM group, the most dominant biological process was localization (56%). In other identified biological processes (biological process involved in interspecies interaction between organisms, cellular process, immune system process, response to stimulus), PSM group proteins had an equal share (11%) (Figure 3B: BP).

The most dominant protein classes of proteins in the GSM group were transfer/carrier protein (39%), metabolite interconversion enzyme (33%), and cytoskeletal protein (16%) (Figure 3A: PC). Moreover, for proteins found in the PSM group, transfer/carrier protein (44%) and metabolite interconversion enzyme (25%) were the most dominant protein classes (Figure 3B: PC).

**Figure 4:**
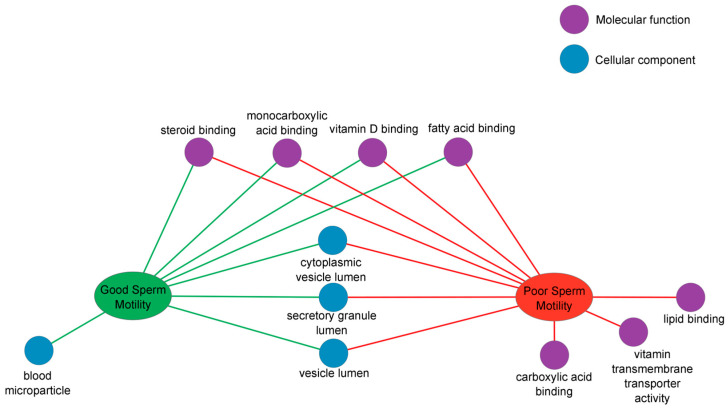
The ToppCluster analysis of proteins associated with the molecular function (purple circle) and cellular component (blue circle). The functional map illustrates shared and group-specific functional annotation terms generated in multicluster protein functional enrichment analysis for proteins identified in the cauda EF of dogs (*Canis lupus familiaris*) divided into two groups according to values of ES progressive motility: GSM (green ellipse) and PSM (red ellipse).

A detailed analysis of the biological categories that differ between experimental groups of dogs (GSM, PSM) and GO terms (molecular function and cellular component) was performed using the ToppCluster tool. This analysis resulted in a network representation of shared and GSM- and PSM-specific GO terms generated by comparing multiple protein lists (Figure 4).

The ToppCluster analysis detailed the molecular functions previously identified during GO analysis using PANTHER. For proteins found in the PSM group, ToppCluster indicates carboxylic acid binding, lipid binding, and vitamin transmembrane transporter activity (Figure 4). Four molecular functions were common for proteins in the GSM and PSM groups: steroid binding, monocarboxylic acid binding, vitamin D binding, and fatty acid binding (Figure 4). The analysis indicated that proteins found in both GSM and PSM groups were components of cytoplasmic vesicle lumen, secretory granule lumen, and vesicle lumen (Figure 4).

#### 2.3.2. The Kyoto Encyclopedia of Genes and Genomes Pathway Analysis

The KEGG pathway analysis showed statistically significant pathways for both GSM and PSM groups. Mainly arachidonic acid metabolism (PTGDS, GPX5) and thyroid hormone synthesis (GPX5, ALB) were significant for both GSM and PSM groups (Table 2). The Hippo signaling pathway (ACTB, AFP) and metabolic pathways (PTGDS, GALNT6, GPX5, ALPL) were characteristic for the GSM group (Table 2), while cholesterol metabolism (NPC2) and glutathione metabolism (GPX5) were specific for the PSM group (Table 2).

#### 2.3.3. Protein–Protein Interaction Network Analysis

**Figure 5:**
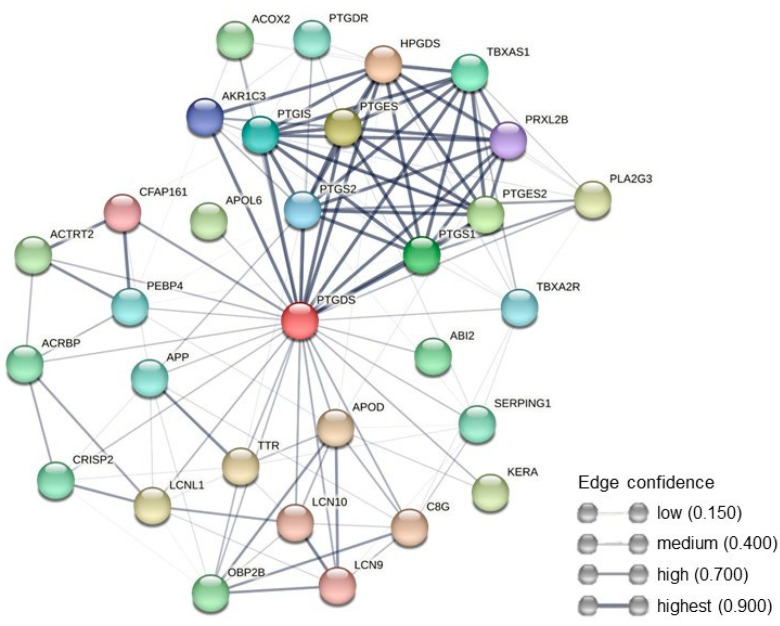
PPI network for Prostaglandin-H2 D-isomerase (PTGDS). The nodes represent proteins, and the edges represent the number of protein–protein interactions. The edges indicate both functional and physical protein associations. Line thickness indicates the strength of data support.

The PPI network analysis revealed that PTGDS could interact with different proteins involved in numerous biological processes (Figure 5). A total of 31 nodes and 135 edges were identified for PTGDS, which revealed PTGDS associations with other members of prostaglandins family, but also with CRISP2, LCNL1, and SERPING1 (Figure 5).

### 2.4. Correlations between Selected Cauda Epididymal Fluid Proteins and Epididymal Spermatozoa Motion Parameters

**Figure 6:**
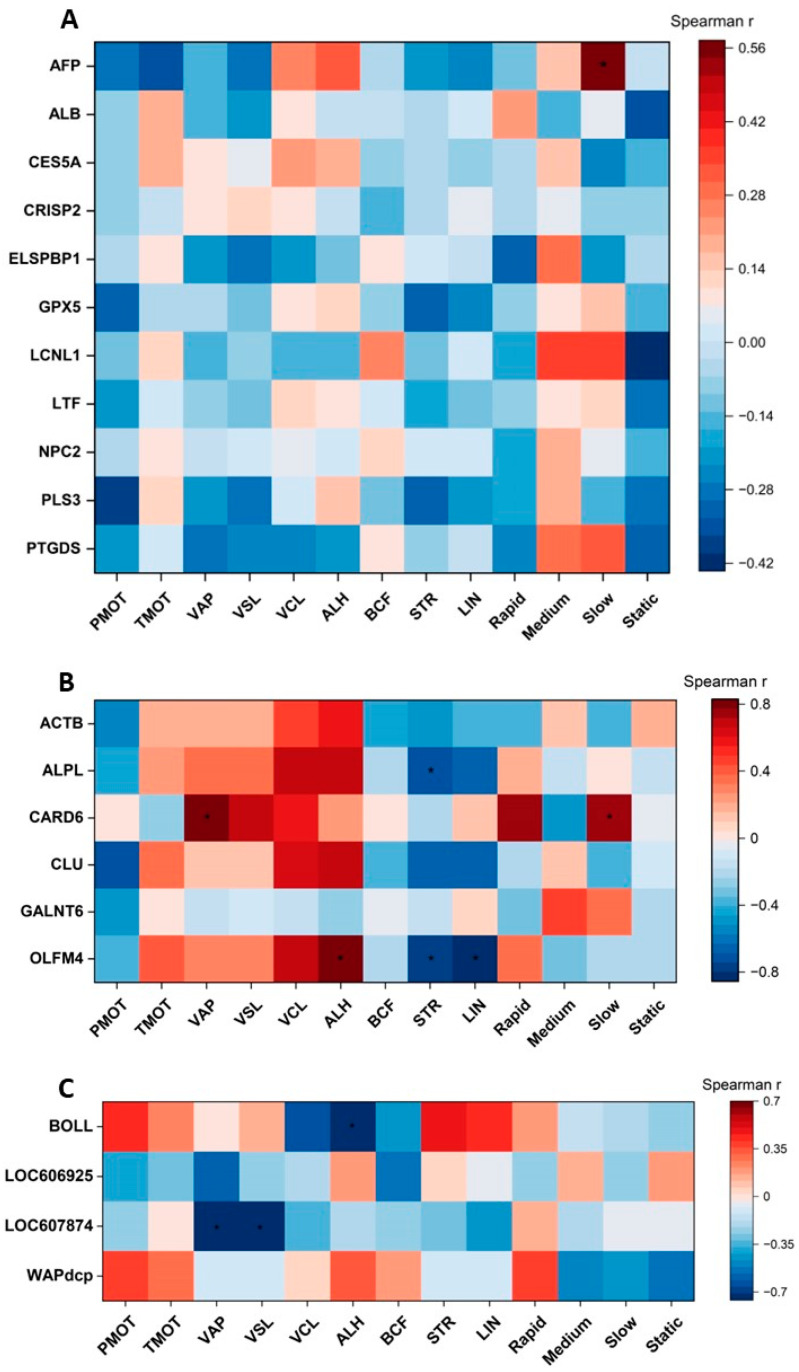
Heatmap showing Spearman’s rank correlation coefficients between the values of the ES motion parameters and the abundance (MS intensity) of the cauda EF proteins (**A**) common for both GSM and PSM groups; (**B**) unique for GSM group; and (**C**) unique for PSM group of dogs (*Canis lupus familiaris*). (*) indicates significant correlations (*p* < 0.05). ACTB—Actin, cytoplasmic 1, AFP—Alpha-fetoprotein, ALPL—Alkaline phosphatase, ALB—Albumin, BOLL—Boule homolog, RNA binding protein, CARD6—Caspase recruitment domain family member 6, CES5A—Carboxylesterase 5A, CLU—Clusterin, CRISP2—Cysteine-rich secretory protein 2, ELSPBP1—Epididymal sperm-binding protein 1, GALNT6—Polypeptide N-acetylgalactosaminyltransferase, GPX5—Epididymal secretory glutathione peroxidase, LCNL1—Lipocln_cytosolic_FA-bd_dom domain-containing protein, LTF—Lactotransferrin, LOC606925—KRAB domain-containing protein, LOC607874—Cystatin domain-containing protein, NPC2—NPC intracellular cholesterol transporter 2, OLFM4—Olfactomedin 4, PLS3—Plastin 3, PTGDS—Prostaglandin-H2 D-isomerase, WAPdcp—WAP domain-containing protein. Motion parameter abbreviations are explained in Table 1.

Spearman’s rank correlation coefficient was used to present the relationships between ES motion parameters and the content (MS intensity) of individual cauda EF proteins. Figure 6A shows Spearman’s rank correlation coefficients between ES motion parameters and the content of cauda EF polypeptides, which is common for both GSM and PSM groups of dogs. No correlations between cauda EF protein contents and ES motion parameters were found, except for a positive correlation between AFP content and the percentage of the slow motion parameter (r = 0.57; *p* < 0.05) (Figure 6A).

Spearman’s rank correlation coefficients were analyzed to evaluate the relationship between the content of the EF proteins unique for the GSM group of dogs and individual ES motion parameters. Positive correlations were observed between CARD6 content and VAP (r = 0.83; *p* < 0.05), but also with a slow motion parameter (r = 0.77; *p* < 0.05) and between OLFM4 content and ALH (r = 0.82; *p* < 0.05) (Figure 6B). Negative correlations were found between ALPL content and STR (r = −0.72; *p* < 0.05), and also OLFM4 and STR (r = −0.77; *p* < 0.05) and LIN (r = −0.85; *p* < 0.05) (Figure 6B).

Spearman’s rank correlation coefficients were also analyzed between the ES motion parameters and the content of the EF polypeptides unique for the PSM group of dogs. Only negative correlations were observed: between BOLL content and ALH (r = −0.76; *p* < 0.05), LOC607874 content and VAP (r = −0.74; *p* < 0.05), and VSL (r = −0.71; *p* < 0.05) (Figure 6C).

### 2.5. Validation of Results by Western Blot

**Figure 7:**
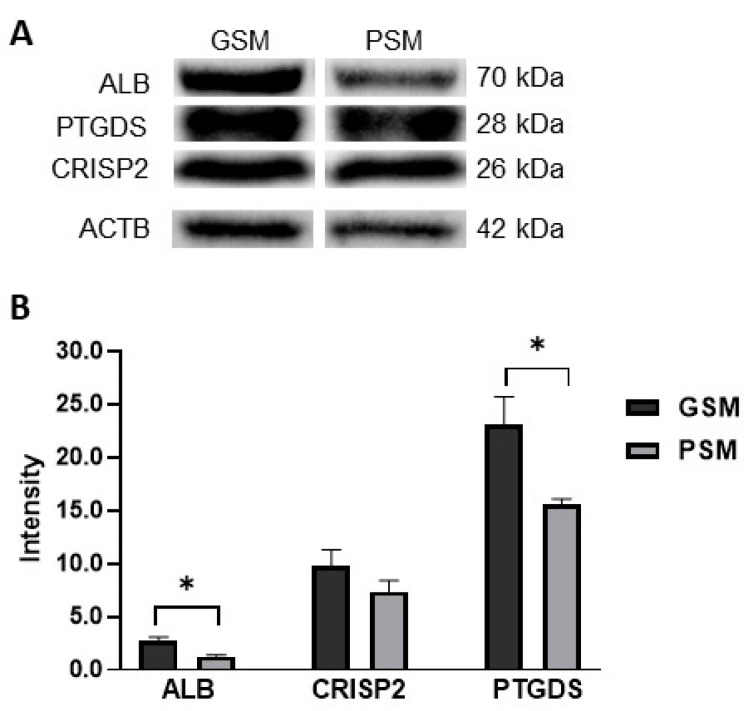
Western blot validation of change in abundances of cauda EF proteins of dogs (*Canis lupus familiaris*) divided into two groups according to values of ES progressive motility: GSM and PSM (**A**). Each column indicates the normalized value (mean ± SEM) of protein abundance from three replicates. Protein abundance represents band intensity, which is significantly different among the analyzed groups. (*) indicates significant differences between GSM and PSM groups at *p* < 0.05 (**B**). ALB—Albumin, ACTB—Actin, cytoplasmic 1, CRISP2—Cysteine-rich secretory protein 2, PTGDS—Prostaglandin-H2 D-isomerase.

To validate and verify the abundance of proteins identified by mass spectrometry, a Western blot (WB) analysis was performed (Figure 7A,B). WB analysis showed a significantly higher abundance of ALB and PTGDS in the GSM group when compared to the PSM group (Figure 7B). WB analysis did not reveal any significant difference in CRISP2 abundance between GSM and PSM groups (Figure 7B).

## 3. Discussion

Proteins found in EF are known to exert an important influence on sperm function and metabolism [1,14,15,16,17]. Each epididymal segment is considered an individual “organ” and creates its unique microenvironment, with its own genes, regulatory proteins, and signal transduction pathways [18]. As part of the search for protein markers of epididymal semen quality, our goal was to check whether some proteins present in the dog’s EF may be related to the quality of spermatozoa present in this fluid as measured by the motion parameters.

The study demonstrated that certain proteins were associated with an EF surrounding ES exhibiting good motility parameters, while another group of proteins was found to be indicative of the EF of ES with poor motility. Proteins unique to the EF of the GSM group may potentially exert a specific positive influence on sperm motion parameters, as shown in the current study. Some UPs found in the canine EF in which spermatozoa were showing poor motion parameters may exert an inhibitory effect on sperm motility. The exact mechanism of this action is specific for each protein and must be discussed separately. However, we cannot rule out that there are functional relationships between some proteins, which was shown in the PPI network.

In the study, proteome profiling of the canine EF was performed using NanoUPLC-Q-TOF/MS for the first time. There are no such reports in world literature, only EF mass spectrometry research in humans [19], boars [20], bulls [21,22,23,24], rams [25,26], and monkeys [27].

In the analyzed groups of semen differing in motility, it was found that the EF homologous with sperm of good motility possesses some UPs, such as ACTB, ALPL, CARD6, CLU, GALNT6, and OLFM4, which are important for the ES metabolism.

ACTB builds the cytoskeleton, flagellar, and acrosomal membrane of spermatozoa [28,29]. It is responsible for cell volume changes, and its role was proposed in sperm capacitation and motility [28,29,30]. ACTB was previously found in the canine ES, and its expression was correlated with the dog’s age [31]. It seems to be a part of the ES structure, but a high abundance of it was found in the EF of the GSM group. The exact mechanism of the EF ACTB influence on ES motility needs to be evaluated in the future.

In dogs, most ALPL is produced in the epididymis [32]. Alkaline phosphatase appears in very high concentrations in canines [33] EF. It plays a role in the transport of sugars and other organic molecules across biological membranes [34]. Its activity has been demonstrated in cytoplasmic droplets, which raises the hypothesis that ALPL catalyzes dephosphorylation and transport of phosphate groups between the ES and EF. The source of the presence of this enzyme in EF could be the intense release of it from the cytoplasmic droplets during the maturation of the ES in the epididymis.

Immune response, cell proliferation, and apoptosis are controlled by proteins containing a caspase recruitment domain (CARD) [35]. CARD6 may be involved in microtubular transport mechanisms. Accordingly, proteins that interact with CARD6 may be targeted to the microtubule-organizing center, resulting in either their inactivation or translocation to a site where they can perform their functions [36]. This protein was found in the canine EF for the first time, and its function in the canine reproductive processes is unknown.

CLU is an extracellular chaperone secreted in high amounts by epididymides [37] and testes [38]. Its presence in the canine ES was established in an earlier study [31]. It participates in sperm maturation by membrane remodeling, takes part in DNA reparation, and it may inhibit cell apoptosis [39]. It could be hypothesized that CLU might be adsorbed from the EF and cover ES plasma membranes while somehow positively influencing ES motility.

Currently, GALNTL was found in male reproductive organs in rodents and cattle [40,41,42,43]. A KEGG pathway analysis indicated that GALNT6 participates in mucin-type-O-glycan biosynthesis in the canine ES [31]. GALNTL5 was localized in the head–tail coupling apparatus of cauda ES in mice [42], and the expression of GALNTL5 was positively correlated with sperm motility [44]. In this study, we showed that GALNT6 is also important for ES motility in canines.

OLFM4 is a glycoprotein for which the absence of gene expression has been shown in human prostate cancer [45,46]. Olfactomedins play essential roles in cell development and differentiation [47]. Olfactomedin-4 precursor was found in human epididymosomes [48]. The role of OLFM4 in ES physiology is unknown, along with its influence on the above-mentioned reproductive cell motility.

Summarizing, it may be hypothesized that the above-mentioned EF proteins exert their essential impact on the ES good motility by participating in sperm cell differentiation, sperm membrane remodeling, and regulating sperm motility.

In EF homologous with poor motility of ES, the following proteins were found to be unique: BOLL, LOC606925, LOC607874, and WAPdcp.

RNA-binding proteins (RBPs), including BOLL, are a group of proteins capable of regulating a plethora of cellular posttranscriptional processes, the perturbation of which leads to impaired spermatogenesis [49]. A reduced number of BOLL mRNA transcripts is associated with spermatogenic failure [50,51]. The presence of BOLL in the EF of the canine was shown for the first time in this study. It might be suggested that it was released into the EF from immature sperm or sperm with impaired spermatogenesis, resulting in reduced sperm motility.

LOC606925 is a protein containing a Krueppel-associated box (KRAB) domain. It was found in about one-third of eukaryotic Krueppel-type C2H2 zinc finger proteins (ZFPs) [52]. The members of the KRAB–ZFPs family are involved in processes including apoptosis, cell proliferation, and tumorigenesis [52]. It has been reported that they are abundantly expressed in the testicles [53,54]. Although this protein might also be released from the ES into the EF milieu, its function and influence on sperm motility have not been determined.

LOC607874 is a cystatin-C-like protein, the content of which was associated with ageing of the canine epididymis [31]. The gene connected with this protein is specific for epididymis [55]. Considering that the presence of LOC607874 has been shown both in the canine ES of old dogs and in the EF with poor sperm motility, this protein might be considered in the future to be a poor epididymal semen quality marker.

Most regions of the canine epididymis show mRNA expression for WAPdcp [56]. Considering that it possesses serine-type endopeptidase inhibitor activity [57], it might be suggested to act as a serine-protease inhibitor specific for canine epididymis. This protein was found in the canine ES membrane [58] and in the EF, which might suggest that it was bound to the sperm plasma membrane or released from spermatozoa to the EF. WAPdcp was found in ES of very young or old dogs [31].

According to the above-mentioned information, we could hypothesize that BOLL, LOC606925, LOC607874, and WAPdcp might be released from dead or impaired spermatozoa to the EF milieu and, due to this fact, were found to be characteristic of lower-quality epididymal semen.

The study showed that 11 proteins were present in both types of dogs EF (GSM and PSM): AFP, ALB, CES5A, CRISP2, ELSPBP1, GPX5, LCNL1, LTF, NPC2, PLS3, and PTGDS. Four of them were selected based on statistical analysis, which showed content variability depending on the motility group. The content of ALB, CRISP2, LCNL1, and PTGDS was higher in the EF surrounding ES with good motility. The presence of ALB, CRISP2, and PTGDS in the canine cauda EF was confirmed by WB analysis. However, statistical differences between analyzed groups were shown only for ALB and PTGDS.

CRISP2 is a cysteine-rich secretory protein (CRISP) family member found in the authors’ previous study of the canine ES [31]. CRISP2 was identified in spermatids [59,60] and in the ejaculated sperm acrosome and sperm tail [61,62,63,64]. A decrease in CRISP2 amounts in the sperm is associated with male infertility [65,66,67]. This fact corresponds with the authors’ recent study in which this protein content in the canine ES cells was lower in very young and senile dogs [31].

LCNL1 is a member of the lipocalin family. These proteins transport or store small molecules, such as vitamins, hormones, and secondary metabolites [68]. LCN proteins are hypothesized to be important for sperm maturation [69]. LCNL1 was expressed in different regions of the epididymis [70]. Epididymal fluid LCNL1 seems to be somehow important for ES sperm motility, but the exact mechanism needs to be investigated.

Albumins are low-molecular weight proteins (about 65 kDa) [71], which are secreted by the testes, epididymides, and prostate. Studies have shown that albumins are implicated in the fertilization-associated events, such as sperm capacitation [72] and the zona pellucida penetration [73]. In one of our previous studies, we have shown that the seminal plasma albumin (ALB) is highly correlated with the motility parameters of stallion spermatozoa [74]. Moreover, albumins have been shown to regulate the number of bivalent cations and possess antioxidant properties [75,76]. It could be suggested that the binding of albumins to the ES plasma membrane might play a protective role in sperm motility [74]. It is likely that the albumins, analyzed in the current study, could have a potential effect on the ES motility by another mechanism in which zinc ions might play a significant role. It has been demonstrated that albumins have a strong affinity for zinc ions, thus reducing their concentrations in spermatozoa [74]. Such a mechanism has been shown to enhance sperm motility [74], and we suggest that a similar mechanism could be responsible for improved ES motility, as shown in the current study.

The presence of PTGDS (prostaglandin-H2 D-isomerase, also known as prostaglandin-D2-synthase, or lipocalin-type prostaglandin-D-synthase [77]) was found in the EF and seminal plasma of rams [26] and cats [78]. It is an enzyme that converts the cyclooxygenase product of prostaglandin H2 (PGH2) to prostaglandin D2 (PGD2). It binds small non-substrate lipophilic molecules such as retinoids [79], which affect the permeability of the plasma membrane, resulting in a greater input of ions from the outside [80]. This phenomenon may be related to the regulation of access of Ca^2+^ ions to the sperm cell, which might be related to sperm motility [1,81]. Intracellular retinol content was shown to positively correlate with sperm motility in humans [82]. A positive correlation between PTGDS content in human sperm and the cells progressive motility was shown [83]. Additionally, PPI networks have revealed that PTGDS is interconnected with several other essential proteins involved in sperm metabolism such as CRISP2, LCNL1, and SERPING1. However, the exact function of PTGDS in ES metabolism is unknown and requires further study.

A broad-range proteomic study revealed that ALB and PTGDS present with greater abundance in EF surrounding sperm with good motility, with high probability are important for the good ES motility.

As immature spermatozoa pass through the epididymis tracts, there is a process of mixing between the EF and the ES, allowing for contact between the substances present in them. The influence of the analyzed proteins on ES mobility should be considered in two aspects. Firstly, the degree of release of proteins from the ES during their maturation or from damaged or immature spermatozoa is important. Secondly, the ES motility might be influenced by secretory proteins produced by the epididymal tissue, which will coat ES and provide changes in the ES surface properties, biochemistry, and metabolism. This metabolically complex environment of the sperm functioning in the epididymis is still a mystery to scientists.

## 4. Materials and Methods

### 4.1. Chemicals and Media

All chemicals of the highest purity were purchased from the Sigma Chemical Company (St. Louis, MO, USA) unless otherwise stated.

### 4.2. Animals

Twenty-three mixed-breed dogs aged from 12 to 132 months (average 72 months) were used in the study. The weight of the dogs was from 9 to 30 kg (mean 19.5 kg). The dogs were kept in the Shelter for Homeless Animals in Tomaryny (Poland) in the same environmental conditions and were fed the standard diet. The dogs were in a program to prevent animal homelessness and promote adoption and were presented for a routine orchiectomy by a qualified veterinary doctor.

### 4.3. Cauda Epididymal Semen Collection

The experimental dogs’ testicles, with the epididymis after removal, were put in sterile plastic bags in a solution of 0.9% NaCl and then placed in a thermobox at a temperature of 4 °C. The tissues were transported within 1 h to the laboratory of the Department of Animal Biochemistry and Biotechnology (University of Warmia and Mazury in Olsztyn, Poland). The gonads were carefully washed with DPBS (Dulbecco’s Phosphate-Buffered Saline, Gibco, Grand Island, NY, USA). A sterile scalpel was used to carefully remove the cauda epididymal tissue from the testicle to avoid damage to the blood vessels. An automatic pipette was used to aspirate the effluent of the epididymal semen, according to Ramos Angrimani et al. (2017) [84], with modification. The samples obtained from the right and left epididymis of the same animal were pooled. Following analyses were performed separately for each dog cauda epididymal semen: cauda epididymal spermatozoa quality assessment, cauda epididymal fluid isolation, preliminary sample preparation for proteomic analysis, and nanoUPLC-Q-TOF/MS analysis.

### 4.4. Cauda Epididymal Spermatozoa Quality Assessment

The ES concentration was determined using a Bürker chamber under a light microscope (Olympus BX41TF, Tokyo, Japan).

A computer-assisted semen analysis (CASA-system, HTM-IVOS 12.3, Hamilton Thorne Biosciences, MA, USA) was used to assess the ES motion parameters. The software settings were described in an earlier study [58]. The procedure was described previously by Mogielnicka-Brzozowska et al. (2020) [78]. The following sperm motion parameters were analyzed in each epididymal semen sample: total motility (TMOT, %), progressive motility (PMOT, %), average path velocity (VAP, µm/s), straight-line velocity (VSL, µm/s), curvilinear velocity (VCL, µm/s), the amplitude of lateral head displacement (ALH, µm), beat cross frequency (BCF, Hz), straightness (STR, %), and linearity coefficient (LIN, %).

After an ES motion parameter assessment, the sperm samples were divided into two experimental groups: good sperm motility (GSM) (n = 13) and poor sperm motility (PSM) (n = 10). The GSM group included the sperm samples showing PMOT ≥ 55%, and the PSM group included the sperm samples with PMOT < 55%. Supplementary Table S3 shows the age of individual dogs and sperm count.

### 4.5. Cauda Epididymal Fluid Isolation

The epididymal semen samples were centrifuged at 800× *g* for 10 min at 4 °C to separate EF from the ES [84]. The supernatants (EF) were immediately collected from above the pellets (ES) and transferred to new Eppendorf tubes and then centrifuged again at 15,000× *g* for 10 min [19]. Then, EF samples were stored for two weeks at −80 °C for further LC-MS analysis.

### 4.6. Preliminary Sample Preparation for Proteomic Analysis

#### 4.6.1. Total Protein Content Measurement

The total protein content in EF was measured using Bradford Reagent (Sigma-Aldrich/B6916).

#### 4.6.2. Sample Preparation for NanoUPLC-Q-TOF/MS Analysis

In order to obtain lipid-free protein extracts, frozen samples were thawed in pre-cooled to −18 °C 800 µL of MTBE:MeOH mixture (3:1). Next, the samples were sonicated in an ice-water-filled sonic bath for 15 min. To pellet the proteins, 400 µL of water:MeOH mixture (3:1) were added to each tube. Next, the Eppendorf tubes were placed in a pre-cooled centrifuge (4 °C) and spun for 7 min at 21,000× *g*. The supernatants were transferred to new tubes and pellets were frozen and kept in −80 °C prior to further analysis.

#### 4.6.3. In-Solution Trypsin Digestion

In-solution protein digestion was processed according to the protocol described by Morelle and Michalski (2007) [85] with some modifications. Protein precipitates were dissolved in 100 µL of 50 mM NH_4_HCO_3_ (pH 8.0) with 0.1% SDS. Freshly prepared DTT solution was added so that the final concentration of DTT was 20 mM, and the sample was incubated at 60 °C for 1 h. The freshly prepared iodoacetamide solution was then added so that the final concentration was 20 mM, and the sample was incubated for 1 h in the dark. The sample was diluted twice, and 5 µg of trypsin were added. The sample was then left overnight with gentle shaking at 37 °C for complete digestion. After trypsinolysis, 1% formic acid was added to adjust the pH to 3–4, and the sample was desalted on Pierce™ C18 columns (Thermo Fisher Scientific, Waltham, MA, USA), then vacuum dried and resuspended in acetonitrile for further LC-MS analysis.

### 4.7. NanoUPLC-Q-TOF/MS Analysis

To separate the digested samples, a Waters Acquity liquid chromatography M-Class system (Waters Corp., Milford, MA, USA) equipped with a Peptide BEH C18 analytical column (150 mm × 75 µm; 1.7 µm, Waters Corp.) and a Symmetry C18 precolumn (180 µm × 20 mm; 1.7 µm, Waters Corp.) was performed. Each sample was injected into the pre-column and washed with 99% solvent A (0.1% formic acid in water). Then, peptides were transferred to an analytical column. A mass spectrometry (MS) using Synapt G2-Si (Waters Corp.) with a nano-electrospray ionization (nESI) source operating under a positive ion mode was performed. Raw chromatography files were analyzed with Byonic software (Protein Metrics, Cupertino, CA, USA). The detected peptides were compared to the SWISSPROT dog proteome (CANLF) downloaded in February 2022. The total intensity was a sum of all the peak intensities over all MS/MS spectra. The protein *p*-value is the likelihood of the peptide-spectrum matches (PSMs) to this protein (or protein group) arising by random chance, according to a simple probabilistic model. A log *p*-value of −3.0 corresponds to a protein *p*-value of 0.001, or one chance in a thousand. A detailed method was described in our previous study [31].

### 4.8. Bioinformatic Analyses and Imaging of Data

A Venn diagram was constructed using a web tool (http://bioinformatics.psb.uugent.be/webtools/Venn, accessed on 13 October 2022).

The functional enrichment of proteins presented in the dog (*Canis lupus familiaris*) cauda EF were analyzed in both groups (GSM, PSM) in gene ontology (GO) categories: molecular function (MF), biological process (BP), and protein class (PC), with the PANTHER Classification System v. 17.0 online tool (http://pantherdb.org, accessed on 20 October 2022).

ToppCluster software [86] was used to identify the following GO categories: molecular function (MF) and cellular component (CC) enriched by proteins identified in the dog (*Canis lupus familiaris*) cauda EF analyzed in groups (GSM, PSM). Results were summarized in a tabular format and visualized as a relationship network using Cytoscape 3.9.1. [87].

Protein–protein interactions (PPI) were obtained and visualized with the use of the STRING v. 11.5 database (http://string-db.org, accessed on 18 October 2022) [88].

The significance of the dog (*Canis lupus familiaris*) cauda EF proteins was analyzed in groups (GSM, PSM) in the Kyoto Encyclopedia of Genes and Genomes (KEGG) pathway database and the KOBAS 3.0 (http://kobas.cbi.pku.edu, accessed on 6 December 2022) protein functional annotation tool [89]. Annotations were performed with the Canis familiaris database, using the false discovery rate (FDR) of the Benjamini–Hochberg method for the significance threshold. The used threshold was 0.05.

Box plots and GO plots were performed using GraphPad PRISM v. 8.0.1 software (GraphPad Software, San Diego, CA, USA). Heatmaps were performed using OriginPro 2023 (OriginLab Corporation, Northampton, MA, USA).

### 4.9. Western Blot Analysis

Epididymal fluid samples containing 50 µg of protein were analyzed using 12% SDS-PAGE and then transferred onto Immobilon-P polyvinylidene fluoride (PVDF) membranes (Millipore, Bedford, MA, USA). Electroblotting was carried out for 1 h at 300 mA, according to the previously described method [90].

The 5% non-fat milk in Tris-buffered saline, TBS (1 M Tris, 5 M NaCl, pH 8.0), containing 0.05% (*v*/*v*) Tween 20, TBST (MP Biomedicals LLC, Santa-Ana, CA, USA) was used for blocking non-specific binding sites. Next, the membranes were incubated with one of the following primary antibodies obtained from Thermo Fisher Scientific: Albumin rabbit polyclonal antibody (1:1000; catalog number PA5-89332;), CRISP2 rabbit polyclonal antibody (1:1000; catalog number PA5-97621), PTGDS rabbit polyclonal antibody (1:1000; catalog number PA1-48023), except Anti-beta Actin antibody (1:2000; catalog number ab8227; Abcam, Cambridge, Great Britain). Despite lengthy searches and correspondence with many manufacturers and suppliers, obtaining an antibody for LCNL1 proved impossible. Primary antibodies were incubated overnight at 4 °C. The membranes were then washed and incubated for 1 h at room temperature with Peroxidase AffiniPure Goat Anti-Rabbit secondary antibody (1:20,000; catalog number 111-035-003; Jackson ImmunoResearch, Baltimore Pike, PA, USA). The ServaLight CL EOS Substrate kit (Serva, Heidelberg, Germany) was used to induce chemiluminescence, and the membranes were then scanned with the ChemiDoc™ Touch Imaging System (Bio-Rad Laboratories, Hercules, CA, USA).

The molecular weight standard (PageRuler™ Prestained Protein Ladder, catalog number 26617, Thermo Fisher Scientific) was used to determine the molecular weights of the proteins. The membranes were scanned, and the intensity of the protein bands was quantified using the Image Lab version 5.2 (Bio-Rad Laboratories). The signal was normalized to a stable beta-Actin protein band. Raw Western blot images were attached to Supplementary Materials as Supplementary Figure S1.

### 4.10. Statistical Analyses

GraphPad PRISM v. 8.0.1 software (GraphPad Software) was used to perform statistical analyses. Epididymal sperm motion parameters were compared between GSM and PSM groups of dogs through a parametric Student’s *t*-test, and a non-parametric Mann–Whitney U test was used when data did not meet normality assumptions. Student’s *t*-test and the Mann–Whitney U test were also used to compare the abundance of proteins common for GSM and PSM groups of dogs. Spearman’s rank coefficient was used to determine the correlations between the relative abundance (MS intensity) of cauda EF proteins and the values of cauda ES motion parameters in dogs. The values were considered to differ significantly at *p* < 0.05.

## 5. Conclusions

Differences in individual protein expression profiles in the canine EF were established and shown to be associated with ES motility parameters. Complete EF proteome profiling could reveal basic knowledge about protein composition and provide essential information of individual protein participation in mechanisms regulating sperm motility. An understanding of the involvement of particular proteins in the sperm functional properties acquisition crucial for the fertilization process can help pinpoint the causes of male infertility and might be used for the construction of commercial tests suitable for semen quality assessment.

## Figures and Tables

**Table 1:** Average motion parameters of the ES of dogs (*Canis lupus familiaris*) divided into two groups according to values of ES progressive motility: GSM and PSM.

Sperm Parameters	Good Sperm Motility (n = 13)	Poor Sperm Motility (n = 10)	*p*-Value
Total motility (TMOT, %)	92.46 ± 0.4	87.9 ± 2.3	0.031
Progressive motility (PMOT, %)	62.08 ± 1.5	37.5 ± 5.2	0.001
Average path velocity (VAP, µm/s)	135.9 ± 3.5	127.8 ± 7.5	0.784
Straight line velocity (VSL, µm/s)	116.1 ± 3.3	99.06 ± 6.3	0.030
Curvilinear velocity (VCL, µm/s)	194.5 ± 5.2	217.3 ± 8.8	0.011
Amplitude of lateral head displacement (ALH, µm)	6.48 ± 0.2	7.68 ± 0.3	0.003
Beat cross frequency (BCF, Hz)	18.51 ± 1.0	14.0 ± 0.5	0.001
Straightness (STR, %)	84.46 ± 0.7	76.3 ± 0.8	0.001
Linearity (LIN, %)	61.92 ± 1.6	49.1 ± 1.4	0.001
Rapid (%)	82.23 ± 1.6	81.38 ± 1.94	0.744
Medium (%)	10.31 ± 1.8	8.37 ± 0.88	0.405
Slow (%)	4.31 ± 0.4	5.63 ± 0.82	0.112
Static (%)	3.15 ± 0.4	4.62 ± 0.73	0.080

Values are represented as the mean ± SEM. Differences between the GSM and PSM groups for TMOT, PMOT, STR, LIN, and rapid, medium, slow, static parameters were compared using Student’s *t*-test. Differences between the GSM and PSM groups for VAP, VSL, VCL, ALH, and BCF parameters were compared using the Mann–Whitney U test. Significant differences with *p* < 0.05.

**Table 2:** The KEGG pathway analysis of the cauda EF proteins of dogs (*Canis lupus familiaris*) divided into two groups according to values of ES progressive motility: GSM and PSM (*p* < 0.05).

GSM				
ID	Pathway Name	Protein Counts	−Log^10^ (Corrected *p*-Value)	Protein Names
cfa00590	Arachidonic acid metabolism	2	4.69 × 10^−4^	PTGDS|GPX5
cfa04918	Thyroid hormone synthesis	2	6.47 × 10^−4^	GPX5|ALB
cfa04390	Hippo signaling pathway	2	2.79 × 10^−3^	ACTB|AFP
cfa01100	Metabolic pathways	4	4.48 × 10^−3^	PTGDS|GALNT6|GPX5|ALPL
**PSM**				
**ID**	**Pathway Name**	**Protein Counts**	**−Log^10^** **(Corrected *p*-Value)**	**Protein Names**
cfa00590	Arachidonic acid metabolism	2	2.16 × 10^−4^	PTGDS|GPX5
cfa04918	Thyroid hormone synthesis	2	2.99 × 10^−4^	GPX5|ALB
cfa04979	Cholesterol metabolism	1	1.71 × 10^−2^	NPC2
cfa00480	Glutathione metabolism	1	1.85 × 10^−2^	GPX5

ACTB—Actin, cytoplasmic 1, AFP—Alpha-fetoprotein, ALB—Albumin, ALPL—Alkaline phosphatase, GALNT6—Polypeptide N-acetylgalactosaminyltransferase, GPX5—Epididymal secretory glutathione peroxidase, NPC2—NPC intracellular cholesterol transporter 2, PTGDS—Prostaglandin-H2 D-isomerase. Significant differences with *p* < 0.05.

## Data Availability

The data underlying this article are available in the article and in its online Supplementary Materials.

## Supplementary Materials

The following supporting information can be downloaded at: https://www.mdpi.com/article/10.3390/ijms241914790/s1.
