# Supplementary material for: Proteome Profiling of Canine Epididymal Fluid: In Search of Protein Markers of Epididymal Sperm Motility
Source: Int J Mol Sci. 2023 Sep 30;24(19):14790. doi: 10.3390/ijms241914790 (PMC10573609; doi:10.3390/ijms241914790)

Supplementary Figure S1. Raw Western blot images showing changes in abundances of selected cauda epididymal fluid proteins of dogs (*Canis lupus familiaris*) divided into two groups according to epididymal sperm progressive motility: good sperm motility (GSM) and poor sperm motility (PSM). ALB—Albumin **(A)**, PTGDS—Prostaglandin-H2 D-isomerase **(B)**, CRISP2—Cysteine-rich secretory protein 2 **(C)**, ACTB—Actin, cytoplasmic 1 **(D)**.

(A) ALB

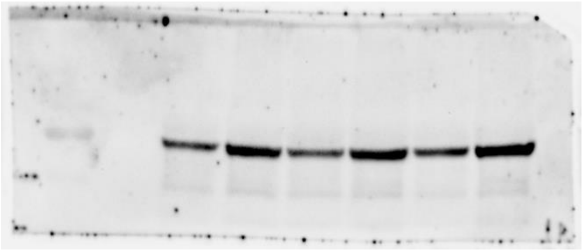

(B) PTGDS

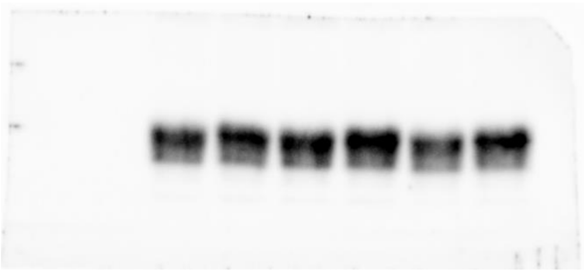

(C) CRISP2

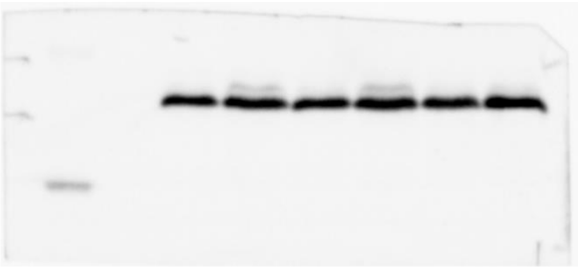

(D) ACTB

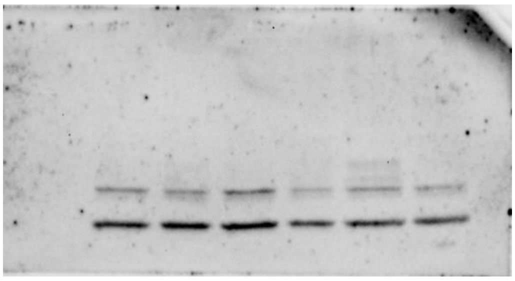

Supplement: Supplementary file 1 [file ijms-24-14790-s001.zip › Supplementary Figure S1.pdf]
