# Supplementary material for: Proteome Profiling of Canine Epididymal Fluid: In Search of Protein Markers of Epididymal Sperm Motility
Source: Int J Mol Sci. 2023 Sep 30;24(19):14790. doi: 10.3390/ijms241914790 (PMC10573609; doi:10.3390/ijms241914790)
Supplement: Supplementary file 1 [file ijms-24-14790-s001.zip › Supplementary Table S1.pdf]

Supplementary Table S1. Proteins of the cauda epididymal fluid of dogs (*Canis lupus familiaris*) with good sperm motility (GSM) evaluated by nanoUPLC-Q-TOF/MS.

Description	|Log Prob|	Best |Log Prob|	Best score	Total Intensity	# of spectra	# of unique peptides	# of mod peptides	Coverage %	# AA's in protein	Protein DB number
>tr|F1PR54|F1PR54_CANLF Lactotransferrin OS=Canis lupus familiaris OX=9615 GN=LTF PE=3 SV=1	126.64	9.12	722.90	1218684786.3	155	33	3	50.56	708	40436
>tr|F1PR54|F1PR54_CANLF Lactotransferrin OS=Canis lupus familiaris OX=9615 GN=LTF PE=3 SV=1	122.87	8.36	687.50	846941286.9	153	34	6	51.13	708	40436
>tr|F1PR54|F1PR54_CANLF Lactotransferrin OS=Canis lupus familiaris OX=9615 GN=LTF PE=3 SV=1	72.13	5.42	600.60	594782951.0	89	25	1	44.49	708	40436
>tr|F1PJ71|F1PJ71_CANLF Glutathione peroxidase OS=Canis lupus familiaris OX=9615 GN=GPX5 PE=3 SV=2	36.68	7.19	628.70	428551591.9	57	9	1	48.42	221	19009
>tr|E2RFZ4|E2RFZ4_CANLF Non-specific serine/threonine protein kinase OS=Canis lupus familiaris OX=9615 GN=SLK PE=4 SV=1	0.14	0.03	165.70	326749944.8	17	1	0	0.40	1242	17002
>sp|Q9XS65|PTGDS_CANLF Prostaglandin-H2 D-isomerase OS=Canis lupus familiaris OX=9615 GN=PTGDS PE=2 SV=1	18.01	5.62	614.50	280176370.2	28	6	3	47.12	191	165
>tr|F1PR54|F1PR54_CANLF Lactotransferrin OS=Canis lupus familiaris OX=9615 GN=LTF PE=3 SV=1	61.88	7.31	653.30	267776591.1	85	22	4	34.04	708	40436
>sp|P49822|ALBU_CANLF Albumin OS=Canis lupus familiaris OX=9615 GN=ALB PE=1 SV=3	59.02	9.73	745.90	253959715.1	80	15	2	36.51	608	490
>sp|Q28895|NPC2_CANLF NPC intracellular cholesterol transporter 2 OS=Canis lupus familiaris OX=9615 GN=NPC2 PE=2 SV=1	24.71	11.54	811.40	234891576.8	37	4	0	38.25	149	153
>sp|Q659K0|CCNB3_CANLF G2/mitotic-specific cyclin-B3 OS=Canis lupus familiaris OX=9615 GN=CCNB3 PE=2 SV=1	1.59	1.30	121.30	223630232.6	17	1	0	0.38	1330	483
>sp|O18840|ACTB_CANLF Actin. cytoplasmic 1 OS=Canis lupus familiaris OX=9615 GN=ACTB PE=2 SV=3	16.24	4.37	415.30	204469348.1	24	8	1	20.53	375	642
>sp|Q9GL25|ESPB1_CANLF Epididymal sperm-binding protein 1 OS=Canis lupus familiaris OX=9615 GN=ELSPBP1 PE=1 SV=1	18.77	4.65	491.00	192341032.2	33	6	2	19.59	245	36
>tr|E2R7F4|E2R7F4_CANLF Olfactory receptor OS=Canis lupus familiaris OX=9615 PE=3 SV=2	0.32	0.10	77.80	167871440.9	13	2	0	1.92	313	14455
>tr|F1PR54|F1PR54_CANLF Lactotransferrin OS=Canis lupus familiaris OX=9615 GN=LTF PE=3 SV=1	65.77	9.01	663.90	162287673.0	78	21	3	36.16	708	40436
>tr|F1PJ71|F1PJ71_CANLF Glutathione peroxidase OS=Canis lupus familiaris OX=9615 GN=GPX5 PE=3 SV=2	16.29	4.76	504.20	157929427.1	24	6	0	32.58	221	19009
>tr|F1PR54|F1PR54_CANLF Lactotransferrin OS=Canis lupus familiaris OX=9615 GN=LTF PE=3 SV=1	49.36	6.79	654.80	157029360.2	53	21	5	42.09	708	40436
>tr|J9P1P6|J9P1P6_CANLF Coiled-coil domain-containing protein 25 OS=Canis lupus familiaris OX=9615 PE=3 SV=2	1.05	0.65	125.00	155323538.9	21	1	0	1.71	292	12368
>sp|Q9XS65|PTGDS_CANLF Prostaglandin-H2 D-isomerase OS=Canis lupus familiaris OX=9615 GN=PTGDS PE=2 SV=1	18.64	7.06	627.00	142743379.4	20	4	3	47.12	191	165
>sp|P49822|ALBU_CANLF Albumin OS=Canis lupus familiaris OX=9615 GN=ALB PE=1 SV=3	8.20	4.36	385.80	124776196.6	13	3	0	6.91	608	490

>sp|O18840|ACTB_CANLF Actin. cytoplasmic 1 OS=Canis lupus familiaris OX=9615 GN=ACTB PE=2 SV=3	9.17	4.97	537.70	117644955.7	12	2	0	9.07	375	642
>tr|J9P1P6|J9P1P6_CANLF Coiled-coil domain-containing protein 25 OS=Canis lupus familiaris OX=9615 PE=3 SV=2	1.89	1.47	113.30	112582603.4	22	1	0	1.71	292	12368
>tr|E2RCT1|E2RCT1_CANLF WAP domain-containing protein OS=Canis lupus familiaris OX=9615 PE=4 SV=2	8.38	8.18	716.60	111314896.6	11	1	0	13.79	116	21717
>sp|Q28894|WFDC2_CANLF WAP four-disulfide core domain protein 2 OS=Canis lupus familiaris OX=9615 GN=WFDC2 PE=2 SV=1	10.65	5.32	550.00	107584263.2	15	4	2	45.16	124	53
>tr|A0A5F4C9S3|A0A5F4C9S3_CANLF Boule homolog. RNA binding protein OS=Canis lupus familiaris OX=9615 GN=BOLL PE=4 SV=1	0.58	0.23	200.20	105145012.8	22	1	0	1.92	365	16536
>sp|O46607|GPX5_CANLF Epididymal secretory glutathione peroxidase OS=Canis lupus familiaris OX=9615 GN=GPX5 PE=2 SV=1	8.94	3.80	359.00	104063830.5	12	3	0	11.31	221	564
>tr|F1PR54|F1PR54_CANLF Lactotransferrin OS=Canis lupus familiaris OX=9615 GN=LTF PE=3 SV=1	37.18	9.06	708.60	100761850.1	49	13	1	19.49	708	40436
>sp|P49822|ALBU_CANLF Albumin OS=Canis lupus familiaris OX=9615 GN=ALB PE=1 SV=3	22.44	5.32	535.70	97451514.9	32	8	1	17.93	608	490
>sp|P25473|CLUS_CANLF Clusterin OS=Canis lupus familiaris OX=9615 GN=CLU PE=2 SV=1	13.06	4.53	483.50	96712175.0	14	4	0	10.56	445	725
>tr|F2Z4Q6|F2Z4Q6_CANLF Alpha fetoprotein OS=Canis lupus familiaris OX=9615 GN=AFP PE=4 SV=2	53.95	11.46	848.50	93904365.5	85	10	0	27.32	637	24990
>tr|F1PR54|F1PR54_CANLF Lactotransferrin OS=Canis lupus familiaris OX=9615 GN=LTF PE=3 SV=1	36.38	7.13	690.00	92477257.7	53	12	1	22.03	708	40436
>sp|P49822|ALBU_CANLF Albumin OS=Canis lupus familiaris OX=9615 GN=ALB PE=1 SV=3	13.13	4.58	458.60	92223673.6	13	3	0	7.89	608	490
>sp|Q6AW47|EST5A_CANLF Carboxylesterase 5A OS=Canis lupus familiaris OX=9615 GN=CES5A PE=2 SV=1	11.67	5.16	536.20	89288745.7	21	3	0	8.35	575	629
>sp|Q9XS65|PTGDS_CANLF Prostaglandin-H2 D-isomerase OS=Canis lupus familiaris OX=9615 GN=PTGDS PE=2 SV=1	9.78	4.69	578.60	88406341.9	15	3	2	39.79	191	165
>sp|Q6AW47|EST5A_CANLF Carboxylesterase 5A OS=Canis lupus familiaris OX=9615 GN=CES5A PE=2 SV=1	12.29	11.99	835.60	85610673.5	16	1	0	3.65	575	629
>tr|E2R6E0|E2R6E0_CANLF Lipocln_cytosolic_FA-bd_dom domain-containing protein OS=Canis lupus familiaris OX=9615 GN=LCNL1 PE=3 SV=2	10.20	4.40	426.00	85200988.7	10	3	0	11.71	299	1932
>sp|Q659K0|CCNB3_CANLF G2/mitotic-specific cyclin-B3 OS=Canis lupus familiaris OX=9615 GN=CCNB3 PE=2 SV=1	1.53	1.29	103.20	81340061.7	13	1	0	0.38	1330	483
>sp|O18840|ACTB_CANLF Actin. cytoplasmic 1 OS=Canis lupus familiaris OX=9615 GN=ACTB PE=2 SV=3	8.98	4.76	409.90	79858615.0	13	3	0	11.47	375	642
>sp|Q659K0|CCNB3_CANLF G2/mitotic-specific cyclin-B3 OS=Canis lupus familiaris OX=9615 GN=CCNB3 PE=2 SV=1	1.45	0.98	129.10	75541895.4	25	1	0	0.38	1330	483
>tr|J9P1P6|J9P1P6_CANLF Coiled-coil domain-containing protein 25 OS=Canis lupus familiaris OX=9615 PE=3 SV=2	0.51	0.23	92.60	75164656.6	15	1	0	1.71	292	12368
>sp|P25473|CLUS_CANLF Clusterin OS=Canis lupus familiaris OX=9615 GN=CLU PE=2 SV=1	7.93	3.57	309.10	68311252.6	6	3	0	7.64	445	725
>tr|F1PR54|F1PR54_CANLF Lactotransferrin OS=Canis lupus familiaris OX=9615 GN=LTF PE=3 SV=1	31.70	5.55	586.50	66958711.4	27	14	1	23.59	708	40436

>sp|Q6AW47|EST5A_CANLF Carboxylesterase 5A OS=Canis lupus familiaris OX=9615 GN=CES5A PE=2 SV=1	8.37	5.46	543.10	66848757.0	19	2	0	6.78	575	629
>tr|F2Z4Q6|F2Z4Q6_CANLF Alpha fetoprotein OS=Canis lupus familiaris OX=9615 GN=AFP PE=4 SV=2	20.55	4.99	540.60	64892825.2	18	7	0	18.05	637	24990
>tr|F1PDT8|F1PDT8_CANLF WAP four-disulfide core domain protein 2 OS=Canis lupus familiaris OX=9615 GN=WFDC2 PE=4 SV=3	18.18	5.87	656.60	63954626.0	18	5	2	50.91	110	33116
>sp|Q28895|NPC2_CANLF NPC intracellular cholesterol transporter 2 OS=Canis lupus familiaris OX=9615 GN=NPC2 PE=2 SV=1	18.41	9.19	746.60	63147505.6	25	3	0	38.25	149	153
>tr|J9NVE0|J9NVE0_CANLF KRAB domain-containing protein OS=Canis lupus familiaris OX=9615 GN=LOC606925 PE=4 SV=1	0.12	0.06	112.50	63104415.6	4	1	0	4.08	147	3752
>sp|Q28895|NPC2_CANLF NPC intracellular cholesterol transporter 2 OS=Canis lupus familiaris OX=9615 GN=NPC2 PE=2 SV=1	13.58	7.54	703.80	61954617.2	17	3	0	36.24	149	153
>sp|Q9GL25|ESPB1_CANLF Epididymal sperm-binding protein 1 OS=Canis lupus familiaris OX=9615 GN=ELSPBP1 PE=1 SV=1	6.92	3.89	373.00	60361363.3	24	3	1	10.20	245	36
>sp|Q6AW47|EST5A_CANLF Carboxylesterase 5A OS=Canis lupus familiaris OX=9615 GN=CES5A PE=2 SV=1	5.80	5.39	546.60	58878724.1	17	2	0	5.74	575	629
>tr|F1PR54|F1PR54_CANLF Lactotransferrin OS=Canis lupus familiaris OX=9615 GN=LTF PE=3 SV=1	28.82	4.76	582.90	56699211.5	37	12	1	22.18	708	40436
>sp|Q9XS65|PTGDS_CANLF Prostaglandin-H2 D-isomerase OS=Canis lupus familiaris OX=9615 GN=PTGDS PE=2 SV=1	4.31	3.95	351.40	56401279.5	5	2	1	18.32	191	165
>tr|F1PR54|F1PR54_CANLF Lactotransferrin OS=Canis lupus familiaris OX=9615 GN=LTF PE=3 SV=1	57.94	7.46	665.70	56113940.3	70	24	0	41.10	708	40436
>tr|A0A5F4CCD0|A0A5F4CCD0_CANLF Cysteine rich secretory protein 2 OS=Canis lupus familiaris OX=9615 GN=CRISP2 PE=3 SV=1	9.32	4.64	490.10	54722108.5	14	4	2	15.76	311	11017
>sp|Q6AW47|EST5A_CANLF Carboxylesterase 5A OS=Canis lupus familiaris OX=9615 GN=CES5A PE=2 SV=1	7.12	4.20	516.10	53058401.5	14	3	0	8.70	575	629
>tr|E2R6E0|E2R6E0_CANLF Lipocln_cytosolic_FA-bd_dom domain-containing protein OS=Canis lupus familiaris OX=9615 GN=LCNL1 PE=3 SV=2	11.87	4.40	566.20	52536400.0	10	3	0	10.37	299	1932
>tr|F1PNV9|F1PNV9_CANLF Pecanex-like protein OS=Canis lupus familiaris OX=9615 GN=PCNX4 PE=3 SV=3	0.10	0.06	114.40	52250931.3	2	1	0	0.52	1145	3914
>sp|Q9GL25|ESPB1_CANLF Epididymal sperm-binding protein 1 OS=Canis lupus familiaris OX=9615 GN=ELSPBP1 PE=1 SV=1	8.15	4.59	352.20	49368920.4	11	2	0	10.20	245	36
>sp|O18840|ACTB_CANLF Actin. cytoplasmic 1 OS=Canis lupus familiaris OX=9615 GN=ACTB PE=2 SV=3	7.85	3.86	473.90	48561650.3	11	2	0	9.07	375	642
>sp|P49822|ALBU_CANLF Albumin OS=Canis lupus familiaris OX=9615 GN=ALB PE=1 SV=3	10.83	4.81	575.30	48366778.3	15	4	2	7.24	608	490
>sp|P49822|ALBU_CANLF Albumin OS=Canis lupus familiaris OX=9615 GN=ALB PE=1 SV=3	9.24	4.60	425.40	48177765.3	7	2	0	4.93	608	490
>sp|Q28895|NPC2_CANLF NPC intracellular cholesterol transporter 2 OS=Canis lupus familiaris OX=9615 GN=NPC2 PE=2 SV=1	18.03	9.37	736.30	48117521.0	28	2	0	24.16	149	153
>sp|Q9XS65|PTGDS_CANLF Prostaglandin-H2 D-isomerase OS=Canis lupus familiaris OX=9615 GN=PTGDS PE=2 SV=1	8.37	5.45	558.30	48074312.6	14	3	2	23.56	191	165
>sp|Q9XS65|PTGDS_CANLF Prostaglandin-H2 D-isomerase OS=Canis lupus familiaris OX=9615 GN=PTGDS PE=2 SV=1	5.87	4.48	537.20	47908769.0	13	3	2	39.79	191	165

>sp|Q9XS65|PTGDS_CANLF Prostaglandin-H2 D-isomerase OS=Canis lupus familiaris OX=9615 GN=PTGDS PE=2 SV=1	7.95	4.10	383.00	47598218.8	17	3	1	32.46	191	165
>tr|A0A5F4CLA9|A0A5F4CLA9_CANLF Kinesin family member 1A OS=Canis lupus familiaris OX=9615 GN=KIF1A PE=3 SV=1	2.68	1.32	205.50	45708942.1	5	3	0	1.18	1696	9452
>tr|F1PLT8|F1PLT8_CANLF Sulfhydryl oxidase OS=Canis lupus familiaris OX=9615 GN=QSOX1 PE=3 SV=3	4.86	4.70	544.70	44597401.6	9	1	0	2.46	568	33056
>sp|Q28895|NPC2_CANLF NPC intracellular cholesterol transporter 2 OS=Canis lupus familiaris OX=9615 GN=NPC2 PE=2 SV=1	9.41	9.23	731.70	43855473.4	10	1	0	15.44	149	153
>tr|A0A5F4CCD0|A0A5F4CCD0_CANLF Cysteine rich secretory protein 2 OS=Canis lupus familiaris OX=9615 GN=CRISP2 PE=3 SV=1	9.94	4.39	424.00	43574399.2	10	3	1	15.76	311	11017
>tr|J9P1P6|J9P1P6_CANLF Coiled-coil domain-containing protein 25 OS=Canis lupus familiaris OX=9615 PE=3 SV=2	1.20	0.82	97.50	43567575.9	20	1	0	1.71	292	12368
>tr|F1PJ71|F1PJ71_CANLF Glutathione peroxidase OS=Canis lupus familiaris OX=9615 GN=GPX5 PE=3 SV=2	14.58	6.51	564.30	43549566.7	14	3	0	31.67	221	19009
>tr|F2Z4Q6|F2Z4Q6_CANLF Alpha fetoprotein OS=Canis lupus familiaris OX=9615 GN=AFP PE=4 SV=2	21.50	4.83	547.00	43284986.2	13	5	0	13.34	637	24990
>tr|E2RCT1|E2RCT1_CANLF WAP domain-containing protein OS=Canis lupus familiaris OX=9615 PE=4 SV=2	7.95	7.81	582.00	40178535.6	8	1	0	13.79	116	21717
>tr|E2RG75|E2RG75_CANLF Inactive ribonuclease-like protein 9 OS=Canis lupus familiaris OX=9615 GN=RNASE9 PE=3 SV=2	12.54	4.13	336.80	39314867.0	8	4	0	37.88	198	41734
>sp|Q659K0|CCNB3_CANLF G2/mitotic-specific cyclin-B3 OS=Canis lupus familiaris OX=9615 GN=CCNB3 PE=2 SV=1	0.67	0.43	127.90	38342036.2	13	1	0	0.38	1330	483
>sp|Q28895|NPC2_CANLF NPC intracellular cholesterol transporter 2 OS=Canis lupus familiaris OX=9615 GN=NPC2 PE=2 SV=1	7.94	7.84	603.20	36789423.5	6	1	0	15.44	149	153
>sp|Q6AW47|EST5A_CANLF Carboxylesterase 5A OS=Canis lupus familiaris OX=9615 GN=CES5A PE=2 SV=1	18.64	7.40	694.20	35054589.9	17	5	0	18.09	575	629
>tr|J9NS29|J9NS29_CANLF Cystatin domain-containing protein OS=Canis lupus familiaris OX=9615 GN=LOC607874 PE=4 SV=2	4.21	4.11	419.90	34212574.4	6	1	0	4.79	313	30016
>sp|Q28895|NPC2_CANLF NPC intracellular cholesterol transporter 2 OS=Canis lupus familiaris OX=9615 GN=NPC2 PE=2 SV=1	9.44	9.16	694.30	33319827.3	15	1	0	15.44	149	153
>tr|F1PB68|F1PB68_CANLF Olfactomedin 4 OS=Canis lupus familiaris OX=9615 GN=OLFM4 PE=4 SV=3	14.51	4.38	411.60	32524389.9	7	4	0	13.21	477	17246
>tr|A0A5F4CQU2|A0A5F4CQU2_CANLF Ig-like domain-containing protein OS=Canis lupus familiaris OX=9615 GN=DLA-DMB PE=3 SV=1	0.11	0.11	107.40	32064177.3	1	1	0	4.10	244	18966
>tr|A0A5F4DFX7|A0A5F4DFX7_CANLF ATP binding cassette subfamily D member 4 OS=Canis lupus familiaris OX=9615 GN=ABCD4 PE=4 SV=1	0.14	0.12	208.30	31860118.0	2	1	0	1.09	548	1012
>tr|E2RC57|E2RC57_CANLF Free fatty acid receptor 1 OS=Canis lupus familiaris OX=9615 GN=FFAR3 PE=3 SV=2	0.19	0.03	117.60	31658657.0	9	1	0	1.98	404	5262
>tr|E2RG75|E2RG75_CANLF Inactive ribonuclease-like protein 9 OS=Canis lupus familiaris OX=9615 GN=RNASE9 PE=3 SV=2	4.86	3.39	304.90	29939099.0	4	2	0	21.21	198	41734
>tr|F1PJ71|F1PJ71_CANLF Glutathione peroxidase OS=Canis lupus familiaris OX=9615 GN=GPX5 PE=3 SV=2	9.01	6.09	605.20	29099093.1	8	2	0	11.76	221	19009
>tr|J9NVE0|J9NVE0_CANLF KRAB domain-containing protein OS=Canis lupus familiaris OX=9615 GN=LOC606925 PE=4 SV=1	0.10	0.01	128.10	29049938.3	6	1	0	4.08	147	3752

>tr|J9NVE0|J9NVE0_CANLF KRAB domain-containing protein OS=Canis lupus familiaris OX=9615 GN=LOC606925 PE=4 SV=1	0.15	0.05	113.30	28768551.6	6	1	0	4.08	147	3752
>tr|A0A5F4C9S3|A0A5F4C9S3_CANLF Boule homolog. RNA binding protein OS=Canis lupus familiaris OX=9615 GN=BOLL PE=4 SV=1	0.14	0.02	198.00	28373181.8	9	1	0	1.92	365	16536
>tr|F1PR54|F1PR54_CANLF Lactotransferrin OS=Canis lupus familiaris OX=9615 GN=LTF PE=3 SV=1	24.29	8.93	656.20	28043851.0	36	7	1	14.97	708	40436
>sp|P49822|ALBU_CANLF Albumin OS=Canis lupus familiaris OX=9615 GN=ALB PE=1 SV=3	14.11	3.92	539.40	27820537.9	23	5	0	12.50	608	490
>tr|A0A5F4D2Z7|A0A5F4D2Z7_CANLF Peptidyl-prolyl cis-trans isomerase OS=Canis lupus familiaris OX=9615 GN=CSNK1G1 PE=3 SV=1	4.08	3.97	253.10	27768145.1	4	2	0	9.89	273	35947
>tr|E2R6E0|E2R6E0_CANLF Lipocln_cytosolic_FA-bd_dom domain-containing protein OS=Canis lupus familiaris OX=9615 GN=LCNL1 PE=3 SV=2	10.57	3.80	502.60	27634392.7	17	3	0	10.37	299	1932
>tr|A0A5F4CLA9|A0A5F4CLA9_CANLF Kinesin family member 1A OS=Canis lupus familiaris OX=9615 GN=KIF1A PE=3 SV=1	0.55	0.32	200.20	26781914.9	6	2	0	0.94	1696	9452
>tr|E2RCT1|E2RCT1_CANLF WAP domain-containing protein OS=Canis lupus familiaris OX=9615 PE=4 SV=2	6.45	6.35	533.20	26369383.5	6	1	0	13.79	116	21717
>tr|J9NWY1|J9NWY1_CANLF Quinoid dihydropteridine reductase OS=Canis lupus familiaris OX=9615 GN=QDPR PE=3 SV=2	2.20	2.16	224.30	25973475.3	3	1	0	6.42	296	22710
>tr|F1PF06|F1PF06_CANLF Sodium channel protein OS=Canis lupus familiaris OX=9615 GN=SCN7A PE=3 SV=3	0.10	0.04	101.60	25772355.2	3	1	0	0.36	1677	19116
>tr|J9NWY1|J9NWY1_CANLF Quinoid dihydropteridine reductase OS=Canis lupus familiaris OX=9615 GN=QDPR PE=3 SV=2	3.51	3.45	266.10	25066880.4	4	1	0	6.42	296	22710
>tr|J9NS29|J9NS29_CANLF Cystatin domain-containing protein OS=Canis lupus familiaris OX=9615 GN=LOC607874 PE=4 SV=2	3.66	3.54	450.40	24807340.7	7	1	0	4.79	313	30016
>tr|J9NWY1|J9NWY1_CANLF Quinoid dihydropteridine reductase OS=Canis lupus familiaris OX=9615 GN=QDPR PE=3 SV=2	2.97	2.93	300.20	24779445.7	3	1	0	6.42	296	22710
>tr|F1PIZ1|F1PIZ1_CANLF Caspase recruitment domain family member 6 OS=Canis lupus familiaris OX=9615 GN=CARD6 PE=4 SV=3	1.39	1.25	173.50	24464975.7	8	1	0	0.65	1071	14260
>tr|F1PB68|F1PB68_CANLF Olfactomedin 4 OS=Canis lupus familiaris OX=9615 GN=OLFM4 PE=4 SV=3	3.35	3.05	243.30	24207005.8	3	2	0	7.13	477	17246
>tr|A0A5F4C6B5|A0A5F4C6B5_CANLF Plastin 3 OS=Canis lupus familiaris OX=9615 GN=PLS3 PE=4 SV=1	13.55	4.07	340.10	23828773.5	6	5	0	16.74	639	30730
>tr|F2Z4Q6|F2Z4Q6_CANLF Alpha fetoprotein OS=Canis lupus familiaris OX=9615 GN=AFP PE=4 SV=2	5.67	3.73	525.40	23650252.7	8	2	1	4.24	637	24990
>tr|A0A5F4C9S3|A0A5F4C9S3_CANLF Boule homolog. RNA binding protein OS=Canis lupus familiaris OX=9615 GN=BOLL PE=4 SV=1	0.11	0.02	202.70	23601704.8	7	1	0	1.92	365	16536
>tr|A0A5F4C687|A0A5F4C687_CANLF Bestrophin OS=Canis lupus familiaris OX=9615 GN=BEST1 PE=3 SV=1	0.15	0.08	129.80	22950697.2	5	1	0	0.90	554	1320
>tr|A0A5F4DGF5|A0A5F4DGF5_CANLF Alkaline phosphatase OS=Canis lupus familiaris OX=9615 GN=ALPL PE=3 SV=1	4.00	3.90	350.90	22901259.6	6	1	0	1.92	572	6357
>tr|F1Q4J2|F1Q4J2_CANLF Carboxypeptidase A6 OS=Canis lupus familiaris OX=9615 GN=CPA6 PE=3 SV=3	0.19	0.15	95.20	22300319.5	3	1	0	1.75	458	7546
>tr|E2RSI6|E2RSI6_CANLF Ezrin OS=Canis lupus familiaris OX=9615 GN=EZR PE=4 SV=1	2.70	2.36	259.70	22126974.4	5	2	0	4.27	586	15650

>tr|E2RPK8|E2RPK8_CANLF Phosphatidylethanolamine binding protein 4 OS=Canis lupus familiaris OX=9615 GN=PEBP4 PE=3 SV=2	6.64	4.71	532.70	21689019.2	3	2	0	12.96	247	4725
>tr|E2RFZ4|E2RFZ4_CANLF Non-specific serine/threonine protein kinase OS=Canis lupus familiaris OX=9615 GN=SLK PE=4 SV=1	0.20	0.19	119.70	21372674.6	4	2	0	2.98	1242	17002
>tr|F1PDT8|F1PDT8_CANLF WAP four-disulfide core domain protein 2 OS=Canis lupus familiaris OX=9615 GN=WFDC2 PE=4 SV=3	3.69	3.67	357.20	21264309.3	2	1	0	29.09	110	33116
>tr|E2R838|E2R838_CANLF Intraflagellar transport 88 OS=Canis lupus familiaris OX=9615 GN=IFT88 PE=4 SV=2	0.10	0.03	103.10	21185094.7	3	1	0	0.73	825	7901
>sp|P25473|CLUS_CANLF Clusterin OS=Canis lupus familiaris OX=9615 GN=CLU PE=2 SV=1	6.26	3.63	351.00	20585729.1	5	3	0	7.64	445	725
>tr|A0A5F4DGF5|A0A5F4DGF5_CANLF Alkaline phosphatase OS=Canis lupus familiaris OX=9615 GN=ALPL PE=3 SV=1	7.31	4.37	477.80	20338059.9	6	2	0	4.37	572	6357
>tr|A0A5F4CI02|A0A5F4CI02_CANLF Solute carrier family 35 member F4 OS=Canis lupus familiaris OX=9615 GN=SLC35F4 PE=3 SV=1	0.25	0.23	128.00	19966156.6	2	1	0	2.29	480	7991
>tr|E2R6E0|E2R6E0_CANLF Lipocln_cytosolic_FA-bd_dom domain-containing protein OS=Canis lupus familiaris OX=9615 GN=LCNL1 PE=3 SV=2	4.48	3.15	410.30	19319897.6	4	2	0	6.69	299	1932
>sp|Q9XS65|PTGDS_CANLF Prostaglandin-H2 D-isomerase OS=Canis lupus familiaris OX=9615 GN=PTGDS PE=2 SV=1	3.99	1.68	276.00	18832900.1	10	5	3	47.12	191	165
>tr|A0A5F4DGF5|A0A5F4DGF5_CANLF Alkaline phosphatase OS=Canis lupus familiaris OX=9615 GN=ALPL PE=3 SV=1	4.74	4.60	471.90	18753180.9	8	1	0	1.92	572	6357
>tr|E2RH40|E2RH40_CANLF Cilia and flagella associated protein 69 OS=Canis lupus familiaris OX=9615 GN=CFAP69 PE=4 SV=2	0.10	0.05	47.10	18417172.3	3	1	0	0.53	941	3287
>tr|E2RSI6|E2RSI6_CANLF Ezrin OS=Canis lupus familiaris OX=9615 GN=EZR PE=4 SV=1	1.32	1.26	212.50	18398388.4	4	1	0	2.90	586	15650
>sp|Q9XS65|PTGDS_CANLF Prostaglandin-H2 D-isomerase OS=Canis lupus familiaris OX=9615 GN=PTGDS PE=2 SV=1	5.60	4.00	440.80	18004374.4	16	3	1	32.46	191	165
>sp|O02812|MK14_CANLF Mitogen-activated protein kinase 14 OS=Canis lupus familiaris OX=9615 GN=MAPK14 PE=2 SV=3	0.24	0.05	106.70	17937349.4	11	1	0	0.83	360	104
>tr|F2Z4Q6|F2Z4Q6_CANLF Alpha fetoprotein OS=Canis lupus familiaris OX=9615 GN=AFP PE=4 SV=2	19.31	3.41	446.00	17706367.3	26	10	0	21.82	637	24990
>tr|E2RPK8|E2RPK8_CANLF Phosphatidylethanolamine binding protein 4 OS=Canis lupus familiaris OX=9615 GN=PEBP4 PE=3 SV=2	4.86	3.61	448.20	17175961.6	4	2	0	12.15	247	4725
>tr|J9P3D0|J9P3D0_CANLF Solute carrier family 4 member 9 OS=Canis lupus familiaris OX=9615 GN=SLC4A9 PE=3 SV=2	1.34	1.30	216.40	17136102.5	3	1	0	1.12	893	31921
>tr|E2RRW9|E2RRW9_CANLF Alpha-galactosidase OS=Canis lupus familiaris OX=9615 GN=NAGA PE=3 SV=2	0.10	0.07	63.10	17079596.2	2	1	1	7.06	411	9615
>tr|J9P1P6|J9P1P6_CANLF Coiled-coil domain-containing protein 25 OS=Canis lupus familiaris OX=9615 PE=3 SV=2	0.91	0.73	103.20	16933519.8	10	1	0	1.71	292	12368
>sp|P63273|RS17_CANLF 40S ribosomal protein S17 OS=Canis lupus familiaris OX=9615 GN=RPS17 PE=2 SV=2	0.11	0.03	99.90	16364382.5	5	1	0	3.70	135	277
>tr|A0A5F4D0R4|A0A5F4D0R4_CANLF RRM domain-containing protein OS=Canis lupus familiaris OX=9615 GN=CPEB1 PE=3 SV=1	0.35	0.31	201.60	16008402.1	2	2	0	2.74	802	10637
>tr|J9NWY1|J9NWY1_CANLF Quinoid dihydropteridine reductase OS=Canis lupus familiaris OX=9615 GN=QDPR PE=3 SV=2	3.52	3.44	360.40	15973260.8	5	1	0	6.42	296	22710

>tr|F1PUC4|F1PUC4_CANLF Olfactory receptor OS=Canis lupus familiaris OX=9615 GN=OR51T1 PE=3 SV=3	0.63	0.59	209.80	15552141.7	3	1	0	2.45	327	41557
>sp|O46607|GPX5_CANLF Epididymal secretory glutathione peroxidase OS=Canis lupus familiaris OX=9615 GN=GPX5 PE=2 SV=1	1.37	0.87	197.60	15539975.4	4	2	0	11.76	221	564
>tr|J9NS29|J9NS29_CANLF Cystatin domain-containing protein OS=Canis lupus familiaris OX=9615 GN=LOC607874 PE=4 SV=2	5.99	4.88	461.10	15223187.0	6	2	0	14.38	313	30016
>sp|Q28894|WFDC2_CANLF WAP four-disulfide core domain protein 2 OS=Canis lupus familiaris OX=9615 GN=WFDC2 PE=2 SV=1	1.20	1.18	507.30	15031324.7	2	1	1	25.81	124	53
>tr|A0A5F4CCD0|A0A5F4CCD0_CANLF Cysteine rich secretory protein 2 OS=Canis lupus familiaris OX=9615 GN=CRISP2 PE=3 SV=1	4.45	4.22	312.30	14786868.4	4	2	1	10.29	311	11017
>sp|O18840|ACTB_CANLF Actin. cytoplasmic 1 OS=Canis lupus familiaris OX=9615 GN=ACTB PE=2 SV=3	3.67	3.39	327.80	14757085.2	9	2	0	7.20	375	642
>tr|E2RCT1|E2RCT1_CANLF WAP domain-containing protein OS=Canis lupus familiaris OX=9615 PE=4 SV=2	5.87	5.79	548.00	14721975.2	5	1	0	13.79	116	21717
>tr|A0A5F4C9S3|A0A5F4C9S3_CANLF Boule homolog. RNA binding protein OS=Canis lupus familiaris OX=9615 GN=BOLL PE=4 SV=1	0.15	0.07	157.00	14716833.9	5	1	0	1.92	365	16536
>tr|E2R8Z9|E2R8Z9_CANLF Glutathione S-transferase C-terminal domain-containing protein OS=Canis lupus familiaris OX=9615 GN=GSTCD PE=3 SV=1	1.47	1.39	353.60	14373247.2	5	1	0	3.66	629	6707
>tr|F1PC86|F1PC86_CANLF SR-related CTD associated factor 8 OS=Canis lupus familiaris OX=9615 GN=SCAF8 PE=4 SV=3	0.12	0.12	214.80	14141295.8	1	1	0	0.39	1282	12800
>tr|E2RQF9|E2RQF9_CANLF Minichromosome maintenance complex component 3 associated protein OS=Canis lupus familiaris OX=9615 GN=MCM3AP PE=4 SV=3	0.73	0.63	310.20	14065856.1	6	1	0	0.25	1980	24083
>tr|A0A5F4D6X1|A0A5F4D6X1_CANLF Cysteine rich secretory protein 2 OS=Canis lupus familiaris OX=9615 GN=CRISP2 PE=3 SV=1	2.54	2.48	507.90	13993639.8	4	1	1	5.30	321	3538
>sp|O46607|GPX5_CANLF Epididymal secretory glutathione peroxidase OS=Canis lupus familiaris OX=9615 GN=GPX5 PE=2 SV=1	2.52	2.42	400.70	13954578.7	6	1	0	6.79	221	564
>tr|F1PJ71|F1PJ71_CANLF Glutathione peroxidase OS=Canis lupus familiaris OX=9615 GN=GPX5 PE=3 SV=2	5.55	2.97	380.60	13930890.2	5	2	0	11.76	221	19009
>sp|O18840|ACTB_CANLF Actin. cytoplasmic 1 OS=Canis lupus familiaris OX=9615 GN=ACTB PE=2 SV=3	4.91	3.87	308.40	13399071.6	4	2	0	9.07	375	642
>tr|E2R819|E2R819_CANLF Ring finger protein 213 OS=Canis lupus familiaris OX=9615 GN=RNF213 PE=4 SV=3	0.75	0.73	87.00	13339333.5	2	1	0	0.65	4454	14353
>tr|F1PR54|F1PR54_CANLF Lactotransferrin OS=Canis lupus familiaris OX=9615 GN=LTF PE=3 SV=1	8.04	2.57	431.00	13047678.1	17	5	0	13.98	708	40436
>tr|A0A5F4CX24|A0A5F4CX24_CANLF Essential for reactive oxygen species protein OS=Canis lupus familiaris OX=9615 GN=CYBC1 PE=3 SV=1	0.29	0.29	54.50	13009478.9	1	1	0	3.03	231	16149
>tr|A0A5F4CNT7|A0A5F4CNT7_CANLF Phosphatidylinositol glycan anchor biosynthesis class G OS=Canis lupus familiaris OX=9615 GN=PIGG PE=4 SV=1	0.20	0.14	173.90	12764824.5	4	1	1	1.06	850	6631
>tr|E2RCK6|E2RCK6_CANLF Hyaluronidase OS=Canis lupus familiaris OX=9615 GN=SPAM1 PE=3 SV=2	8.78	4.31	482.00	12701239.0	6	3	0	13.31	541	3939
>tr|A0A5F4C6B5|A0A5F4C6B5_CANLF Plastin 3 OS=Canis lupus familiaris OX=9615 GN=PLS3 PE=4 SV=1	6.69	2.98	300.20	12651962.3	6	3	0	11.58	639	30730

>tr|J9NSQ1|J9NSQ1_CANLF Ig-like domain-containing protein OS=Canis lupus familiaris OX=9615 PE=4 SV=2	4.30	3.91	352.90	12418811.9	7	2	0	29.17	120	9570
>tr|A0A5F4C2J2|A0A5F4C2J2_CANLF Alpha-mannosidase OS=Canis lupus familiaris OX=9615 GN=MAN2B1 PE=3 SV=1	4.76	4.68	373.90	12267576.2	5	1	0	2.21	1042	3130
>tr|A0A5F4C6B5|A0A5F4C6B5_CANLF Plastin 3 OS=Canis lupus familiaris OX=9615 GN=PLS3 PE=4 SV=1	4.61	2.69	390.10	12209620.1	5	2	0	5.95	639	30730
>tr|A0A5F4C2Y3|A0A5F4C2Y3_CANLF Carboxypeptidase X. M14 family member 1 OS=Canis lupus familiaris OX=9615 GN=CPXM1 PE=3 SV=1	0.61	0.55	77.70	12178494.5	4	1	0	1.21	661	21027
>tr|A0A5F4C4P7|A0A5F4C4P7_CANLF G_PROTEIN_RECEP_F1_2 domain-containing protein OS=Canis lupus familiaris OX=9615 PE=4 SV=1	0.73	0.73	102.10	12128979.2	1	1	0	3.24	309	30871
>tr|A0A5F4C739|A0A5F4C739_CANLF Rho guanine nucleotide exchange factor 15 OS=Canis lupus familiaris OX=9615 GN=ARHGEF15 PE=4 SV=1	0.32	0.29	54.50	11800443.6	3	1	0	0.79	885	14702
>tr|A0A5F4CLA9|A0A5F4CLA9_CANLF Kinesin family member 1A OS=Canis lupus familiaris OX=9615 GN=KIF1A PE=3 SV=1	0.51	0.29	225.70	11560522.9	2	2	0	0.94	1696	9452
>tr|A0A5F4D8I6|A0A5F4D8I6_CANLF Phospholipase A2 receptor 1 OS=Canis lupus familiaris OX=9615 GN=PLA2R1 PE=4 SV=1	0.12	0.10	164.80	11534982.4	3	2	1	0.50	1394	6796
>tr|F1PLT8|F1PLT8_CANLF Sulfhydryl oxidase OS=Canis lupus familiaris OX=9615 GN=QSOX1 PE=3 SV=3	3.96	3.88	379.10	11399383.9	5	1	0	2.46	568	33056
>tr|F1PRL1|F1PRL1_CANLF Dppa2_A domain-containing protein OS=Canis lupus familiaris OX=9615 PE=4 SV=2	0.12	0.02	52.40	11309431.9	6	1	0	2.79	179	17222
>sp|Q9XS65|PTGDS_CANLF Prostaglandin-H2 D-isomerase OS=Canis lupus familiaris OX=9615 GN=PTGDS PE=2 SV=1	1.20	1.10	254.10	11294616.9	3	2	1	32.46	191	165
>tr|A0A5F4C6B5|A0A5F4C6B5_CANLF Plastin 3 OS=Canis lupus familiaris OX=9615 GN=PLS3 PE=4 SV=1	5.62	3.74	575.20	11155603.6	7	2	0	9.39	639	30730
>tr|A0A5F4C6L3|A0A5F4C6L3_CANLF Cyclic nucleotide binding domain containing 1 OS=Canis lupus familiaris OX=9615 GN=CNBD1 PE=4 SV=1	0.10	0.06	98.30	11131387.5	3	1	0	1.07	563	8180
>tr|A0A5F4D2Z7|A0A5F4D2Z7_CANLF Peptidyl-prolyl cis-trans isomerase OS=Canis lupus familiaris OX=9615 GN=CSNK1G1 PE=3 SV=1	2.12	1.68	221.80	10857197.2	4	2	0	9.89	273	35947
>tr|Q5TJG5|Q5TJG5_CANLF MHC class II antigen DO alpha OS=Canis lupus familiaris OX=9615 GN=DLA-DOA PE=3 SV=1	0.49	0.47	151.70	10744635.5	2	1	0	4.00	250	41318
>sp|Q9XS65|PTGDS_CANLF Prostaglandin-H2 D-isomerase OS=Canis lupus familiaris OX=9615 GN=PTGDS PE=2 SV=1	2.18	2.06	365.30	10719513.5	7	2	1	23.56	191	165
>sp|Q9GL25|ESPB1_CANLF Epididymal sperm-binding protein 1 OS=Canis lupus familiaris OX=9615 GN=ELSPBP1 PE=1 SV=1	2.05	2.05	152.60	10607549.0	1	1	0	6.94	245	36
>tr|F6XSF4|F6XSF4_CANLF Shugoshin 1 OS=Canis lupus familiaris OX=9615 GN=SGO1 PE=3 SV=2	0.10	0.02	83.50	10337506.8	2	1	0	1.14	527	40477
>tr|A0A5F4C3M5|A0A5F4C3M5_CANLF IQ motif containing GTPase activating protein 2 OS=Canis lupus familiaris OX=9615 GN=IQGAP2 PE=4 SV=1	0.77	0.61	117.30	10166444.1	9	1	0	0.51	1577	1056
>tr|A0A5F4D4B6|A0A5F4D4B6_CANLF Immunoglobulin superfamily member 8 OS=Canis lupus familiaris OX=9615 GN=IGSF8 PE=4 SV=1	0.22	0.21	144.90	10123758.1	2	1	0	1.99	905	16467
>tr|Q9XSV4|Q9XSV4_CANLF CE10 protein OS=Canis lupus familiaris OX=9615 GN=ce10 PE=2 SV=1	1.97	1.93	178.80	10022413.4	3	1	0	9.09	110	41542
>sp|P49822|ALBU_CANLF Albumin OS=Canis lupus familiaris OX=9615 GN=ALB PE=1 SV=3	10.07	3.61	411.70	9863051.9	13	4	0	9.70	608	490

>tr|A0A5F4C6L3|A0A5F4C6L3_CANLF Cyclic nucleotide binding domain containing 1 OS=Canis lupus familiaris OX=9615 GN=CNBD1 PE=4 SV=1	0.10	0.05	84.80	9825785.2	3	1	0	1.07	563	8180
>tr|F1PB68|F1PB68_CANLF Olfactomedin 4 OS=Canis lupus familiaris OX=9615 GN=OLFM4 PE=4 SV=3	4.54	3.21	375.40	9680575.6	3	2	0	7.55	477	17246
>tr|J9NS29|J9NS29_CANLF Cystatin domain-containing protein OS=Canis lupus familiaris OX=9615 GN=LOC607874 PE=4 SV=2	3.61	3.59	299.40	9618285.7	2	1	0	4.79	313	30016
>tr|J9P1P6|J9P1P6_CANLF Coiled-coil domain-containing protein 25 OS=Canis lupus familiaris OX=9615 PE=3 SV=2	0.48	0.24	70.40	9490796.3	13	1	0	1.71	292	12368
>tr|A0A5F4D4B6|A0A5F4D4B6_CANLF Immunoglobulin superfamily member 8 OS=Canis lupus familiaris OX=9615 GN=IGSF8 PE=4 SV=1	0.88	0.86	167.30	9320732.8	2	1	0	1.99	905	16467
>sp|Q6AW47|EST5A_CANLF Carboxylesterase 5A OS=Canis lupus familiaris OX=9615 GN=CES5A PE=2 SV=1	8.06	7.87	627.20	9277638.4	7	2	1	8.35	575	629
>tr|A0A5F4CKD5|A0A5F4CKD5_CANLF Polypeptide N-acetylgalactosaminyltransferase OS=Canis lupus familiaris OX=9615 GN=GALNT6 PE=3 SV=1	3.47	3.39	364.10	9235958.9	5	1	0	4.97	644	1617
>tr|J9PBB1|J9PBB1_CANLF Glutathione-independent PGD synthase OS=Canis lupus familiaris OX=9615 GN=PTGDS PE=3 SV=2	6.76	3.94	416.80	9171796.5	11	3	1	21.55	181	8521
>tr|E2R6E0|E2R6E0_CANLF Lipocln_cytosolic_FA-bd_dom domain-containing protein OS=Canis lupus familiaris OX=9615 GN=LCNL1 PE=3 SV=2	2.84	2.82	291.90	9019710.2	2	1	0	3.01	299	1932
>tr|F6XVF5|F6XVF5_CANLF WD repeat domain 72 OS=Canis lupus familiaris OX=9615 GN=WDR72 PE=4 SV=2	0.75	0.73	118.90	9019664.3	2	1	0	0.75	1071	16644
>tr|J9NVM9|J9NVM9_CANLF Transporter OS=Canis lupus familiaris OX=9615 GN=SLC6A17 PE=3 SV=1	0.20	0.20	203.00	9005241.1	1	1	0	2.06	727	21199
>sp|P25473|CLUS_CANLF Clusterin OS=Canis lupus familiaris OX=9615 GN=CLU PE=2 SV=1	3.26	1.99	269.80	8924448.6	6	3	0	7.64	445	725
>tr|F1PP44|F1PP44_CANLF Sterol regulatory element binding transcription factor 1 OS=Canis lupus familiaris OX=9615 GN=SREBF1 PE=4 SV=3	0.25	0.20	151.10	8820859.2	4	1	0	0.87	1147	16638
>tr|F1PIZ1|F1PIZ1_CANLF Caspase recruitment domain family member 6 OS=Canis lupus familiaris OX=9615 GN=CARD6 PE=4 SV=3	1.03	0.93	106.00	8750342.3	6	1	0	0.65	1071	14260
>sp|B6V8E6|CTNB1_CANLF Catenin beta-1 OS=Canis lupus familiaris OX=9615 GN=CTNNB1 PE=1 SV=1	1.30	1.30	138.60	8729708.8	1	1	0	0.51	781	442
>tr|F1PMC5|F1PMC5_CANLF Phosphatidylinositol 4-kinase alpha OS=Canis lupus familiaris OX=9615 GN=PI4KA PE=3 SV=3	0.75	0.73	66.30	8610412.2	2	1	0	0.33	2101	27051
>tr|A0A5F4DGF5|A0A5F4DGF5_CANLF Alkaline phosphatase OS=Canis lupus familiaris OX=9615 GN=ALPL PE=3 SV=1	1.70	1.64	270.80	8523199.0	4	1	0	1.92	572	6357
>sp|Q9GL25|ESPB1_CANLF Epididymal sperm-binding protein 1 OS=Canis lupus familiaris OX=9615 GN=ELSPBP1 PE=1 SV=1	0.12	0.07	200.10	8504093.5	4	2	1	3.27	245	36
>sp|Q28259|G3P_CANLF Glyceraldehyde-3-phosphate dehydrogenase OS=Canis lupus familiaris OX=9615 GN=GAPDH PE=2 SV=3	0.42	0.42	156.80	8480953.0	1	1	0	4.20	333	501
>tr|A0A5F4CYM0|A0A5F4CYM0_CANLF Quiescin sulfhydryl oxidase 2 OS=Canis lupus familiaris OX=9615 GN=QSOX2 PE=4 SV=1	1.10	1.06	210.00	8083901.5	3	1	0	1.42	636	1464
>sp|Q28895|NPC2_CANLF NPC intracellular cholesterol transporter 2 OS=Canis lupus familiaris OX=9615 GN=NPC2 PE=2 SV=1	8.12	4.78	492.10	7983135.5	4	2	0	24.16	149	153
>tr|E2R0D9|E2R0D9_CANLF Solute carrier family 17 member 2 OS=Canis lupus familiaris OX=9615 GN=SLC17A2 PE=4 SV=1	0.31	0.31	142.60	7931457.5	1	1	0	2.51	478	30466

>tr|A0A5F4BNS5|A0A5F4BNS5_CANLF PPARG related coactivator 1 OS=Canis lupus familiaris OX=9615 GN=PPRC1 PE=4 SV=1	0.13	0.13	161.10	7783008.2	1	1	1	2.80	1392	10740
>tr|A0A5F4DGF5|A0A5F4DGF5_CANLF Alkaline phosphatase OS=Canis lupus familiaris OX=9615 GN=ALPL PE=3 SV=1	2.22	2.18	328.50	7655483.7	3	1	0	1.92	572	6357
>tr|A0A5F4DGF5|A0A5F4DGF5_CANLF Alkaline phosphatase OS=Canis lupus familiaris OX=9615 GN=ALPL PE=3 SV=1	2.02	1.04	226.40	7583609.5	4	2	0	4.37	572	6357
>tr|E2RCT1|E2RCT1_CANLF WAP domain-containing protein OS=Canis lupus familiaris OX=9615 PE=4 SV=2	5.54	5.46	532.70	7483646.1	5	1	0	13.79	116	21717
>tr|A0A5F4C3Q4|A0A5F4C3Q4_CANLF Polypyrimidine tract binding protein 2 OS=Canis lupus familiaris OX=9615 GN=PTBP2 PE=4 SV=1	1.01	0.99	150.50	7379660.1	2	1	0	2.25	356	10982
>tr|F1PRT7|F1PRT7_CANLF Cation-transporting ATPase OS=Canis lupus familiaris OX=9615 GN=ATP13A4 PE=3 SV=2	0.13	0.09	140.90	7289314.1	3	1	0	0.67	1197	1348
>tr|F1PHZ1|F1PHZ1_CANLF Plexin A2 OS=Canis lupus familiaris OX=9615 GN=PLXNA2 PE=3 SV=3	0.16	0.16	251.90	7286242.9	1	1	1	1.00	1906	44095
>tr|J9NS29|J9NS29_CANLF Cystatin domain-containing protein OS=Canis lupus familiaris OX=9615 GN=LOC607874 PE=4 SV=2	2.78	2.74	260.90	7214607.5	3	1	0	4.79	313	30016
>sp|B6V8E6|CTNB1_CANLF Catenin beta-1 OS=Canis lupus familiaris OX=9615 GN=CTNNB1 PE=1 SV=1	0.46	0.46	156.30	7147571.1	1	1	0	0.51	781	442
>tr|A0A5F4D3T4|A0A5F4D3T4_CANLF IQ motif containing GTPase activating protein 1 OS=Canis lupus familiaris OX=9615 GN=IQGAP1 PE=4 SV=1	0.16	0.12	124.70	6877867.8	4	2	0	0.84	1548	4417
>sp|Q28895|NPC2_CANLF NPC intracellular cholesterol transporter 2 OS=Canis lupus familiaris OX=9615 GN=NPC2 PE=2 SV=1	5.00	4.98	426.10	6873389.7	2	1	0	15.44	149	153
>tr|A0A5F4BNQ6|A0A5F4BNQ6_CANLF Ena/VASP-like protein OS=Canis lupus familiaris OX=9615 GN=EVL PE=4 SV=1	0.32	0.33	257.60	6761642.3	1	1	1	1.55	837	17395
>tr|E2R8Z9|E2R8Z9_CANLF Glutathione S-transferase C-terminal domain-containing protein OS=Canis lupus familiaris OX=9615 GN=GSTCD PE=3 SV=1	0.97	0.97	257.60	6532782.0	1	1	0	3.66	629	6707
>tr|F1PKE7|F1PKE7_CANLF C-type lectin domain containing 16A OS=Canis lupus familiaris OX=9615 GN=CLEC16A PE=3 SV=3	0.29	0.29	160.70	6532297.5	1	1	0	1.52	990	2034
>tr|F1PB68|F1PB68_CANLF Olfactomedin 4 OS=Canis lupus familiaris OX=9615 GN=OLFM4 PE=4 SV=3	1.48	1.18	170.20	6521989.0	2	2	0	6.92	477	17246
>sp|P31637|SC5A3_CANLF Sodium/myo-inositol cotransporter OS=Canis lupus familiaris OX=9615 GN=SLC5A3 PE=2 SV=1	1.08	1.08	154.30	6358453.9	1	1	0	0.56	718	62
>tr|A0A5F4C6B5|A0A5F4C6B5_CANLF Plastin 3 OS=Canis lupus familiaris OX=9615 GN=PLS3 PE=4 SV=1	1.13	0.93	224.30	6330344.2	3	3	0	14.55	639	30730
>tr|J9NVE0|J9NVE0_CANLF KRAB domain-containing protein OS=Canis lupus familiaris OX=9615 GN=LOC606925 PE=4 SV=1	0.10	0.06	112.00	6297681.1	2	1	0	4.08	147	3752
>sp|Q6AW47|EST5A_CANLF Carboxylesterase 5A OS=Canis lupus familiaris OX=9615 GN=CES5A PE=2 SV=1	6.99	3.53	413.20	6241052.4	4	2	0	6.78	575	629
>tr|F1PIZ1|F1PIZ1_CANLF Caspase recruitment domain family member 6 OS=Canis lupus familiaris OX=9615 GN=CARD6 PE=4 SV=3	0.92	0.86	163.00	6092617.3	4	1	0	0.65	1071	14260
>sp|Q7YRU7|DSG3_CANLF Desmoglein-3 OS=Canis lupus familiaris OX=9615 GN=DSG3 PE=2 SV=1	0.10	0.08	61.70	6054166.9	1	1	0	2.32	993	508
>tr|A0A5F4CTN2|A0A5F4CTN2_CANLF Zinc finger CCHC domain-containing protein 4 OS=Canis lupus familiaris OX=9615 GN=ZCCHC4 PE=3 SV=1	0.12	0.12	175.70	6020006.2	1	1	1	2.96	506	8047

>tr|F1PM28|F1PM28_CANLF Complex I subunit B13 OS=Canis lupus familiaris OX=9615 PE=3 SV=3	0.75	0.73	90.10	5928330.2	2	1	0	8.64	81	3261
>tr|E2RF73|E2RF73_CANLF DNA helicase OS=Canis lupus familiaris OX=9615 GN=MCM3 PE=3 SV=3	0.12	0.10	105.10	5387011.1	2	1	0	1.96	817	14486
>tr|A0A5F4D9S5|A0A5F4D9S5_CANLF Hyaluronoglucosaminidase OS=Canis lupus familiaris OX=9615 GN=CEMIP PE=3 SV=1	0.44	0.44	152.00	5369280.1	1	1	0	0.24	1684	9775
>sp|O46607|GPX5_CANLF Epididymal secretory glutathione peroxidase OS=Canis lupus familiaris OX=9615 GN=GPX5 PE=2 SV=1	1.43	1.37	263.00	5345706.2	4	1	0	6.79	221	564
>tr|F1PJ71|F1PJ71_CANLF Glutathione peroxidase OS=Canis lupus familiaris OX=9615 GN=GPX5 PE=3 SV=2	5.88	2.84	315.20	5260544.0	8	3	0	18.55	221	19009
>sp|Q05052|OST48_CANLF Dolichyl-diphosphooligosaccharide--protein glycosyltransferase 48 kDa subunit OS=Canis lupus familiaris OX=9615 GN=DDOST PE=1 SV=1	1.70	1.70	253.00	5232268.1	1	1	0	0.90	445	254
>tr|F1PE21|F1PE21_CANLF Tubulin alpha chain OS=Canis lupus familiaris OX=9615 GN=LOC477570 PE=3 SV=2	4.57	3.46	316.10	5218487.2	3	2	1	7.56	450	14589
>sp|Q6AW47|EST5A_CANLF Carboxylesterase 5A OS=Canis lupus familiaris OX=9615 GN=CES5A PE=2 SV=1	2.78	2.76	405.00	5212677.7	2	1	0	3.13	575	629
>tr|F1PEN8|F1PEN8_CANLF Carboxylic ester hydrolase OS=Canis lupus familiaris OX=9615 GN=BCHE PE=3 SV=1	0.11	0.09	139.70	5191221.4	2	1	0	1.33	602	4179
>tr|A0A5F4CKD5|A0A5F4CKD5_CANLF Polypeptide N-acetylgalactosaminyltransferase OS=Canis lupus familiaris OX=9615 GN=GALNT6 PE=3 SV=1	1.07	1.05	222.00	5155218.6	2	1	0	4.97	644	1617
>sp|Q6AW47|EST5A_CANLF Carboxylesterase 5A OS=Canis lupus familiaris OX=9615 GN=CES5A PE=2 SV=1	3.79	3.78	353.40	5083490.0	2	1	0	3.13	575	629
>tr|F1PKE7|F1PKE7_CANLF C-type lectin domain containing 16A OS=Canis lupus familiaris OX=9615 GN=CLEC16A PE=3 SV=3	1.04	1.04	171.70	5076150.0	1	1	0	1.52	990	2034
>tr|F1PIZ1|F1PIZ1_CANLF Caspase recruitment domain family member 6 OS=Canis lupus familiaris OX=9615 GN=CARD6 PE=4 SV=3	0.10	0.08	61.10	5024515.0	2	1	0	0.65	1071	14260
>tr|F1PLT8|F1PLT8_CANLF Sulfhydryl oxidase OS=Canis lupus familiaris OX=9615 GN=QSOX1 PE=3 SV=3	1.75	1.75	246.90	4971443.1	1	1	0	1.76	568	33056
>tr|A0A5F4CKD5|A0A5F4CKD5_CANLF Polypeptide N-acetylgalactosaminyltransferase OS=Canis lupus familiaris OX=9615 GN=GALNT6 PE=3 SV=1	1.93	1.91	310.40	4825124.1	2	1	0	4.97	644	1617
>tr|A0A5F4BYH0|A0A5F4BYH0_CANLF Exportin 6 OS=Canis lupus familiaris OX=9615 GN=XPO6 PE=3 SV=1	0.28	0.28	45.30	4819807.6	1	1	0	1.77	1071	11957
>tr|A0A5F4D6X1|A0A5F4D6X1_CANLF Cysteine rich secretory protein 2 OS=Canis lupus familiaris OX=9615 GN=CRISP2 PE=3 SV=1	0.11	0.11	183.50	4795280.9	1	1	1	5.30	321	3538
>sp|P11615|ADA1B_CANLF Alpha-1B adrenergic receptor (Fragment) OS=Canis lupus familiaris OX=9615 GN=ADRA1B PE=2 SV=1	0.10	0.05	153.50	4707576.7	1	1	0	2.40	417	676
>tr|F1PI87|F1PI87_CANLF Obg-like ATPase 1 OS=Canis lupus familiaris OX=9615 GN=OLA1 PE=3 SV=3	0.10	0.07	125.00	4608422.0	1	1	1	4.10	415	24107
>sp|P25291|GP2_CANLF Pancreatic secretory granule membrane major glycoprotein GP2 OS=Canis lupus familiaris OX=9615 GN=GP2 PE=1 SV=1	0.21	0.21	160.40	4570702.1	1	1	0	2.36	509	142
>tr|E2RPK8|E2RPK8_CANLF Phosphatidylethanolamine binding protein 4 OS=Canis lupus familiaris OX=9615 GN=PEBP4 PE=3 SV=2	4.31	4.25	464.70	4527926.9	4	1	0	8.91	247	4725

>tr|A0A5F4BY35|A0A5F4BY35_CANLF Myosin IIIA OS=Canis lupus familiaris OX=9615 GN=MYO3A PE=3 SV=1	0.39	0.37	242.60	4472307.5	2	2	2	2.72	1473	18205
>tr|E2RR34|E2RR34_CANLF V-type proton ATPase subunit a OS=Canis lupus familiaris OX=9615 GN=ATP6V0A4 PE=3 SV=2	0.25	0.24	90.10	4362840.4	2	1	0	2.86	839	21320
>tr|A0A5F4DGF5|A0A5F4DGF5_CANLF Alkaline phosphatase OS=Canis lupus familiaris OX=9615 GN=ALPL PE=3 SV=1	1.11	1.09	183.60	4341403.1	2	1	0	1.92	572	6357
>tr|E2R0Z6|E2R0Z6_CANLF Junctophilin OS=Canis lupus familiaris OX=9615 GN=JPH1 PE=3 SV=1	0.17	0.12	129.90	4319466.1	4	1	0	1.06	662	32945
>tr|A0A5F4C6B5|A0A5F4C6B5_CANLF Plastin 3 OS=Canis lupus familiaris OX=9615 GN=PLS3 PE=4 SV=1	1.98	1.94	391.30	4292584.5	3	1	0	4.07	639	30730
>tr|E2REZ4|E2REZ4_CANLF Coiled-coil and C2 domain containing 2A OS=Canis lupus familiaris OX=9615 GN=CC2D2A PE=4 SV=3	0.22	0.22	298.60	4239053.6	1	1	0	0.76	1585	29419
>tr|E2RT80|E2RT80_CANLF ADP ribosylation factor GTPase activating protein 3 OS=Canis lupus familiaris OX=9615 GN=ARFGAP3 PE=4 SV=3	0.73	0.73	44.80	4168676.5	1	1	0	4.86	473	36754
>sp|P25473|CLUS_CANLF Clusterin OS=Canis lupus familiaris OX=9615 GN=CLU PE=2 SV=1	2.15	2.15	278.00	4152171.9	1	1	1	4.49	445	725
>tr|E2RQC4|E2RQC4_CANLF WW domain-containing oxidoreductase OS=Canis lupus familiaris OX=9615 GN=WWOX PE=4 SV=3	0.13	0.12	156.60	4125433.8	2	1	0	2.05	391	23035
>sp|Q7YRB7|AOFB_CANLF Amine oxidase [flavin-containing] B OS=Canis lupus familiaris OX=9615 GN=MAOB PE=2 SV=3	0.14	0.12	114.40	4125433.8	2	1	0	1.54	520	646
>tr|J9P758|J9P758_CANLF Sorcin OS=Canis lupus familiaris OX=9615 GN=SRI PE=4 SV=2	0.29	0.29	165.60	4008996.6	1	1	0	2.30	348	7242
>sp|P61162|ACTZ_CANLF Alpha-centractin OS=Canis lupus familiaris OX=9615 GN=ACTR1A PE=2 SV=1	0.71	0.71	103.70	4005625.4	1	1	0	5.59	376	693
>sp|Q8WNN6|SODC_CANLF Superoxide dismutase [Cu-Zn] OS=Canis lupus familiaris OX=9615 GN=SOD1 PE=2 SV=1	0.83	0.83	250.10	3990437.1	1	1	0	23.53	153	6
>tr|A0A5F4CKD5|A0A5F4CKD5_CANLF Polypeptide N-acetylgalactosaminyltransferase OS=Canis lupus familiaris OX=9615 GN=GALNT6 PE=3 SV=1	2.71	2.69	342.60	3971920.7	2	1	0	4.97	644	1617
>sp|P25291|GP2_CANLF Pancreatic secretory granule membrane major glycoprotein GP2 OS=Canis lupus familiaris OX=9615 GN=GP2 PE=1 SV=1	0.28	0.28	162.10	3949608.5	1	1	0	2.36	509	142
>tr|F1PLV2|F1PLV2_CANLF Peptidyl-prolyl cis-trans isomerase OS=Canis lupus familiaris OX=9615 GN=CSNK1G1 PE=3 SV=3	1.09	1.09	183.00	3904986.1	1	1	0	5.35	243	4290
>sp|Q9GL25|ESPB1_CANLF Epididymal sperm-binding protein 1 OS=Canis lupus familiaris OX=9615 GN=ELSPBP1 PE=1 SV=1	0.10	0.05	161.20	3884693.1	2	1	0	3.27	245	36
>tr|A0A5F4CDM1|A0A5F4CDM1_CANLF L-lactate dehydrogenase OS=Canis lupus familiaris OX=9615 PE=3 SV=1	1.29	1.25	146.40	3883074.0	3	1	0	2.87	279	43073
>tr|F1PIZ1|F1PIZ1_CANLF Caspase recruitment domain family member 6 OS=Canis lupus familiaris OX=9615 GN=CARD6 PE=4 SV=3	1.27	1.23	134.70	3756557.7	3	1	0	0.65	1071	14260
>tr|A0A5F4BQ34|A0A5F4BQ34_CANLF Galectin-3-binding protein OS=Canis lupus familiaris OX=9615 GN=CANT1 PE=4 SV=1	1.22	1.22	222.50	3755470.5	1	1	0	4.27	539	12448
>tr|A0A5F4C2Y3|A0A5F4C2Y3_CANLF Carboxypeptidase X. M14 family member 1 OS=Canis lupus familiaris OX=9615 GN=CPXM1 PE=3 SV=1	0.94	0.90	121.00	3671195.5	3	1	0	1.21	661	21027
>tr|E2RS75|E2RS75_CANLF C3/C5 convertase OS=Canis lupus familiaris OX=9615 GN=CFB PE=4 SV=2	0.57	0.53	109.00	3645960.3	3	1	0	0.85	823	1888

>tr|Q9XSV4|Q9XSV4_CANLF CE10 protein OS=Canis lupus familiaris OX=9615 GN=ce10 PE=2 SV=1	1.46	1.46	151.20	3617459.7	1	1	0	9.09	110	41542
>tr|F1PH13|F1PH13_CANLF Potassium channel tetramerization domain containing 8 OS=Canis lupus familiaris OX=9615 GN=KCTD8 PE=4 SV=3	0.20	0.20	197.30	3591566.5	1	1	0	5.08	472	2616
>tr|E2REQ4|E2REQ4_CANLF NIPA like domain containing 3 OS=Canis lupus familiaris OX=9615 GN=NIPAL3 PE=3 SV=3	0.10	0.02	140.70	3561431.3	4	1	0	2.17	368	39451
>tr|A0A5F4DFY8|A0A5F4DFY8_CANLF Splicing factor 3b subunit 3 OS=Canis lupus familiaris OX=9615 GN=SF3B3 PE=4 SV=1	0.31	0.31	179.40	3543830.3	1	1	0	0.93	1185	5042
>sp|O46607|GPX5_CANLF Epididymal secretory glutathione peroxidase OS=Canis lupus familiaris OX=9615 GN=GPX5 PE=2 SV=1	2.60	2.54	370.40	3504534.0	4	1	0	6.79	221	564
>tr|F1PB68|F1PB68_CANLF Olfactomedin 4 OS=Canis lupus familiaris OX=9615 GN=OLFM4 PE=4 SV=3	2.95	2.93	306.10	3486039.8	2	1	0	4.40	477	17246
>tr|F1Q4J2|F1Q4J2_CANLF Carboxypeptidase A6 OS=Canis lupus familiaris OX=9615 GN=CPA6 PE=3 SV=3	0.87	0.79	93.20	3431049.3	5	1	0	1.75	458	7546
>tr|F1PRT7|F1PRT7_CANLF Cation-transporting ATPase OS=Canis lupus familiaris OX=9615 GN=ATP13A4 PE=3 SV=2	0.28	0.29	120.40	3396727.6	1	1	0	0.67	1197	1348
>tr|E2R269|E2R269_CANLF Ubiquitinyl hydrolase 1 OS=Canis lupus familiaris OX=9615 GN=VCPIP1 PE=4 SV=1	2.05	2.05	198.00	3370842.9	1	1	0	1.39	1220	40849
>tr|A0A5F4DFI0|A0A5F4DFI0_CANLF Modulator of VRAC current 1 OS=Canis lupus familiaris OX=9615 GN=MLC1 PE=4 SV=1	0.22	0.22	149.90	3331619.3	1	1	0	1.10	724	31348
>tr|A0A5F4CX24|A0A5F4CX24_CANLF Essential for reactive oxygen species protein OS=Canis lupus familiaris OX=9615 GN=CYBC1 PE=3 SV=1	0.22	0.22	82.50	3299520.3	1	1	0	3.03	231	16149
>tr|F6XVF5|F6XVF5_CANLF WD repeat domain 72 OS=Canis lupus familiaris OX=9615 GN=WDR72 PE=4 SV=2	1.02	0.98	146.30	3234741.3	3	1	0	0.75	1071	16644
>tr|E2R5P5|E2R5P5_CANLF Calcyclin OS=Canis lupus familiaris OX=9615 GN=S100A8 PE=4 SV=2	0.10	0.08	193.70	3229992.8	2	1	0	14.66	191	27843
>tr|A0A5F4C3M5|A0A5F4C3M5_CANLF IQ motif containing GTPase activating protein 2 OS=Canis lupus familiaris OX=9615 GN=IQGAP2 PE=4 SV=1	1.21	1.20	138.00	3212578.1	2	1	0	0.51	1577	1056
>tr|E2R8Z9|E2R8Z9_CANLF Glutathione S-transferase C-terminal domain-containing protein OS=Canis lupus familiaris OX=9615 GN=GSTCD PE=3 SV=1	0.35	0.35	150.40	3208617.7	1	1	0	3.66	629	6707
>sp|O46607|GPX5_CANLF Epididymal secretory glutathione peroxidase OS=Canis lupus familiaris OX=9615 GN=GPX5 PE=2 SV=1	9.91	9.53	709.00	3166249.4	6	3	0	26.70	221	564
>tr|F1PIZ1|F1PIZ1_CANLF Caspase recruitment domain family member 6 OS=Canis lupus familiaris OX=9615 GN=CARD6 PE=4 SV=3	0.50	0.48	132.30	3134967.7	2	1	0	0.65	1071	14260
>tr|F1PB68|F1PB68_CANLF Olfactomedin 4 OS=Canis lupus familiaris OX=9615 GN=OLFM4 PE=4 SV=3	4.93	2.49	273.90	3097096.9	4	2	0	7.55	477	17246
>tr|A0A5F4C840|A0A5F4C840_CANLF KIAA1109 OS=Canis lupus familiaris OX=9615 GN=KIAA1109 PE=4 SV=1	0.29	0.29	167.70	3093149.0	1	1	0	0.34	5041	21640
>tr|A0A5F4C6B5|A0A5F4C6B5_CANLF Plastin 3 OS=Canis lupus familiaris OX=9615 GN=PLS3 PE=4 SV=1	1.26	1.24	247.50	3084067.1	2	2	0	6.26	639	30730
>tr|E2RFZ5|E2RFZ5_CANLF Cilia and flagella associated protein 61 OS=Canis lupus familiaris OX=9615 GN=CFAP61 PE=4 SV=1	0.20	0.18	102.70	3078613.1	2	2	2	4.12	1237	12378

>tr|F1Q407|F1Q407_CANLF Ventricular zone expressed PH domain containing 1 OS=Canis lupus familiaris OX=9615 GN=VEPH1 PE=3 SV=2	0.10	0.05	132.10	3061290.7	2	1	0	0.72	833	10639
>tr|F1PCE5|F1PCE5_CANLF Serpin family A member 1 OS=Canis lupus familiaris OX=9615 GN=SERPINA1 PE=3 SV=3	2.31	1.26	232.60	3054966.1	2	2	0	8.79	421	8323
>tr|A0A5F4CX24|A0A5F4CX24_CANLF Essential for reactive oxygen species protein OS=Canis lupus familiaris OX=9615 GN=CYBC1 PE=3 SV=1	0.28	0.29	102.10	3044705.1	1	1	0	3.03	231	16149
>tr|E2R5P5|E2R5P5_CANLF Calcyclin OS=Canis lupus familiaris OX=9615 GN=S100A8 PE=4 SV=2	1.72	1.68	372.20	3036362.5	3	1	0	14.66	191	27843
>tr|A0A5F4C2J2|A0A5F4C2J2_CANLF Alpha-mannosidase OS=Canis lupus familiaris OX=9615 GN=MAN2B1 PE=3 SV=1	2.51	2.49	231.20	3029024.0	2	1	0	2.21	1042	3130
>tr|J9NRX4|J9NRX4_CANLF Glutathione transferase OS=Canis lupus familiaris OX=9615 GN=LOC481841 PE=3 SV=2	4.42	4.40	504.10	3025548.4	2	1	0	10.98	246	15581
>tr|A0A5F4C3Q4|A0A5F4C3Q4_CANLF Polypyrimidine tract binding protein 2 OS=Canis lupus familiaris OX=9615 GN=PTBP2 PE=4 SV=1	0.25	0.23	191.40	3025548.4	2	1	0	2.25	356	10982
>tr|Q5TJG5|Q5TJG5_CANLF MHC class II antigen DO alpha OS=Canis lupus familiaris OX=9615 GN=DLA-DOA PE=3 SV=1	1.15	1.13	82.30	2971764.4	2	1	0	4.00	250	41318
>sp|P30804|ADCY6_CANLF Adenylate cyclase type 6 OS=Canis lupus familiaris OX=9615 GN=ADCY6 PE=1 SV=1	0.10	0.08	146.60	2962920.6	1	1	1	1.89	1165	641
>tr|A0A5F4C6B5|A0A5F4C6B5_CANLF Plastin 3 OS=Canis lupus familiaris OX=9615 GN=PLS3 PE=4 SV=1	0.94	0.49	269.60	2947256.0	3	2	0	9.39	639	30730
>tr|E2RAK5|E2RAK5_CANLF Sulfotransferase OS=Canis lupus familiaris OX=9615 GN=SULT1C3 PE=3 SV=1	0.13	0.09	152.90	2946696.1	3	1	0	2.30	304	12938
>tr|F6XSF4|F6XSF4_CANLF Shugoshin 1 OS=Canis lupus familiaris OX=9615 GN=SGO1 PE=3 SV=2	0.12	0.12	72.50	2909919.9	1	1	0	1.14	527	40477
>sp|Q5ECR9|CCR5_CANLF C-C chemokine receptor type 5 OS=Canis lupus familiaris OX=9615 GN=CCR5 PE=2 SV=1	0.10	0.08	143.30	2885580.0	1	1	0	3.41	352	687
>tr|A0A5F4C3V3|A0A5F4C3V3_CANLF Olfactory receptor OS=Canis lupus familiaris OX=9615 GN=OR4C11I PE=3 SV=1	0.55	0.55	47.40	2841254.4	1	1	0	4.81	312	9939
>tr|A0A5F4CKD5|A0A5F4CKD5_CANLF Polypeptide N-acetylgalactosaminyltransferase OS=Canis lupus familiaris OX=9615 GN=GALNT6 PE=3 SV=1	5.34	3.45	281.50	2836813.8	2	2	0	7.30	644	1617
>tr|A0A5F4C3M5|A0A5F4C3M5_CANLF IQ motif containing GTPase activating protein 2 OS=Canis lupus familiaris OX=9615 GN=IQGAP2 PE=4 SV=1	0.12	0.09	113.00	2830856.7	3	1	0	0.51	1577	1056
>tr|F1P6I0|F1P6I0_CANLF V-set immunoregulatory receptor OS=Canis lupus familiaris OX=9615 GN=VSIR PE=4 SV=3	0.10	0.08	43.60	2805260.8	1	1	0	1.65	423	42941
>tr|E2RAK5|E2RAK5_CANLF Sulfotransferase OS=Canis lupus familiaris OX=9615 GN=SULT1C3 PE=3 SV=1	0.10	0.05	149.00	2770170.5	3	1	0	2.30	304	12938
>tr|F1PBG8|F1PBG8_CANLF DNA_MISMATCH_REPAIR_2 domain-containing protein OS=Canis lupus familiaris OX=9615 GN=MSH5 PE=3 SV=3	0.45	0.45	187.70	2760880.3	1	1	0	1.26	793	42812
>tr|J9NVE0|J9NVE0_CANLF KRAB domain-containing protein OS=Canis lupus familiaris OX=9615 GN=LOC606925 PE=4 SV=1	0.11	0.09	88.20	2745429.5	2	1	0	4.08	147	3752
>tr|E2RDG6|E2RDG6_CANLF V(D)J recombination-activating protein 2 OS=Canis lupus familiaris OX=9615 GN=RAG2 PE=3 SV=1	0.10	0.10	97.70	2745009.6	1	1	1	6.45	527	29372

>tr|A0A5F4CPA9|A0A5F4CPA9_CANLF Bromodomain containing 4 OS=Canis lupus familiaris OX=9615 GN=BRD4 PE=4 SV=1	0.14	0.12	89.80	2733515.2	2	1	1	10.27	146	26797
>tr|E2RL61|E2RL61_CANLF Myomesin 3 OS=Canis lupus familiaris OX=9615 GN=MYOM3 PE=4 SV=2	0.18	0.18	225.50	2720606.2	1	1	0	3.11	1449	15946
>tr|E2R0Z6|E2R0Z6_CANLF Junctophilin OS=Canis lupus familiaris OX=9615 GN=JPH1 PE=3 SV=1	0.30	0.26	120.60	2715959.2	3	1	0	1.06	662	32945
>tr|A0A5F4C9S3|A0A5F4C9S3_CANLF Boule homolog. RNA binding protein OS=Canis lupus familiaris OX=9615 GN=BOLL PE=4 SV=1	0.79	0.77	194.30	2710834.2	2	1	0	1.92	365	16536
>tr|A0A5F4C2Y3|A0A5F4C2Y3_CANLF Carboxypeptidase X. M14 family member 1 OS=Canis lupus familiaris OX=9615 GN=CPXM1 PE=3 SV=1	0.33	0.31	98.60	2704153.1	2	1	0	1.21	661	21027
>tr|J9NZM4|J9NZM4_CANLF Ataxin-10 OS=Canis lupus familiaris OX=9615 GN=ATXN10 PE=3 SV=1	0.45	0.45	115.80	2691060.1	1	1	0	1.89	475	1508
>tr|F1PGF7|F1PGF7_CANLF Acyltransferase OS=Canis lupus familiaris OX=9615 GN=AWAT1 PE=3 SV=2	0.15	0.11	30.30	2688651.0	3	1	0	5.64	337	4210
>tr|J9P6S2|J9P6S2_CANLF Solute carrier family 12 member 4 OS=Canis lupus familiaris OX=9615 GN=SLC12A4 PE=3 SV=2	0.24	0.22	62.80	2685987.2	2	1	0	0.66	1055	3880
>tr|E2RHS5|E2RHS5_CANLF tRNA (guanine-N(7)-)-methyltransferase non-catalytic subunit WDR4 OS=Canis lupus familiaris OX=9615 GN=WDR4 PE=3 SV=1	0.21	0.17	99.60	2652408.1	3	1	0	1.72	406	22738
>sp|Q28894|WFDC2_CANLF WAP four-disulfide core domain protein 2 OS=Canis lupus familiaris OX=9615 GN=WFDC2 PE=2 SV=1	2.32	2.15	216.20	2623082.3	6	2	1	12.90	124	53
>sp|Q6AW47|EST5A_CANLF Carboxylesterase 5A OS=Canis lupus familiaris OX=9615 GN=CES5A PE=2 SV=1	3.73	2.76	391.10	2589539.7	4	2	0	6.78	575	629
>tr|A0A5F4D4B6|A0A5F4D4B6_CANLF Immunoglobulin superfamily member 8 OS=Canis lupus familiaris OX=9615 GN=IGSF8 PE=4 SV=1	1.30	1.28	184.40	2566159.8	2	1	0	1.99	905	16467
>tr|A0A5F4C3Z0|A0A5F4C3Z0_CANLF Metaxin 2 OS=Canis lupus familiaris OX=9615 GN=MTX2 PE=3 SV=1	0.86	0.87	279.40	2522383.9	1	1	0	16.10	267	14491
>tr|E2R6E0|E2R6E0_CANLF Lipocln_cytosolic_FA-bd_dom domain-containing protein OS=Canis lupus familiaris OX=9615 GN=LCNL1 PE=3 SV=2	4.18	4.04	438.20	2516704.3	8	1	0	3.68	299	1932
>tr|F2Z4Q6|F2Z4Q6_CANLF Alpha fetoprotein OS=Canis lupus familiaris OX=9615 GN=AFP PE=4 SV=2	1.34	1.34	210.60	2500545.4	1	1	0	2.04	637	24990
>tr|A0A5F4CDJ1|A0A5F4CDJ1_CANLF Transforming growth factor beta receptor 3 OS=Canis lupus familiaris OX=9615 GN=TGFBR3 PE=4 SV=1	0.73	0.73	69.20	2484850.5	1	1	0	0.91	770	10452
>tr|F1PLT8|F1PLT8_CANLF Sulfhydryl oxidase OS=Canis lupus familiaris OX=9615 GN=QSOX1 PE=3 SV=3	4.25	4.23	402.50	2478425.1	2	1	0	3.35	568	33056
>tr|J9P434|J9P434_CANLF Myotubularin related protein 14 OS=Canis lupus familiaris OX=9615 GN=MTMR14 PE=4 SV=2	0.39	0.37	122.60	2466216.2	2	1	0	1.51	596	17413
>tr|A0A5F4C6B5|A0A5F4C6B5_CANLF Plastin 3 OS=Canis lupus familiaris OX=9615 GN=PLS3 PE=4 SV=1	1.11	0.94	176.10	2429891.2	2	2	0	9.39	639	30730
>tr|J9P9J4|J9P9J4_CANLF Aldehyde dehydrogenase 1 family member A1 OS=Canis lupus familiaris OX=9615 GN=ALDH1A1 PE=3 SV=1	3.57	3.57	379.70	2410549.9	1	1	0	2.69	484	13765
>tr|J9NT93|J9NT93_CANLF SCP domain-containing protein OS=Canis lupus familiaris OX=9615 PE=3 SV=2	0.19	0.17	149.60	2373504.0	2	1	0	15.26	190	43704
>tr|F1PRL1|F1PRL1_CANLF Dppa2_A domain-containing protein OS=Canis lupus familiaris OX=9615 PE=4 SV=2	0.10	0.02	56.80	2349058.8	4	1	0	2.79	179	17222

>tr|A0A5F4CQU2|A0A5F4CQU2_CANLF Ig-like domain-containing protein OS=Canis lupus familiaris OX=9615 GN=DLA-DMB PE=3 SV=1	0.17	0.17	137.00	2299643.1	1	1	0	4.10	244	18966
>tr|J9P432|J9P432_CANLF Glutamine--fructose-6-phosphate transaminase (isomerizing) OS=Canis lupus familiaris OX=9615 GN=GFPT1 PE=4 SV=2	0.11	0.11	190.30	2288263.6	1	1	0	1.18	677	7191
>tr|A0A5F4CDM1|A0A5F4CDM1_CANLF L-lactate dehydrogenase OS=Canis lupus familiaris OX=9615 PE=3 SV=1	0.39	0.37	134.60	2285583.9	2	1	0	2.87	279	43073
>tr|A0A5F4CKD5|A0A5F4CKD5_CANLF Polypeptide N-acetylgalactosaminyltransferase OS=Canis lupus familiaris OX=9615 GN=GALNT6 PE=3 SV=1	1.49	1.47	307.80	2263491.3	2	1	0	4.97	644	1617
>sp|Q28895|NPC2_CANLF NPC intracellular cholesterol transporter 2 OS=Canis lupus familiaris OX=9615 GN=NPC2 PE=2 SV=1	3.68	3.66	442.50	2255318.2	2	1	0	15.44	149	153
>tr|A0A5F4CGS8|A0A5F4CGS8_CANLF Olfactory receptor OS=Canis lupus familiaris OX=9615 GN=OR6C63 PE=3 SV=1	0.11	0.11	41.90	2225580.3	1	1	0	8.06	310	26555
>tr|A0A5F4CCD0|A0A5F4CCD0_CANLF Cysteine rich secretory protein 2 OS=Canis lupus familiaris OX=9615 GN=CRISP2 PE=3 SV=1	2.41	2.33	268.00	2221665.5	5	1	0	4.82	311	11017
>tr|A0A5F4D799|A0A5F4D799_CANLF MPN domain containing OS=Canis lupus familiaris OX=9615 GN=MPND PE=4 SV=1	0.82	0.80	104.40	2187291.3	2	1	0	3.07	618	8215
>tr|A0A5F4CIC9|A0A5F4CIC9_CANLF Prolylcarboxypeptidase OS=Canis lupus familiaris OX=9615 GN=PRCP PE=3 SV=1	0.10	0.05	30.40	2137369.6	1	1	0	7.78	450	20776
>tr|A0A5F4CQU2|A0A5F4CQU2_CANLF Ig-like domain-containing protein OS=Canis lupus familiaris OX=9615 GN=DLA-DMB PE=3 SV=1	0.41	0.41	100.10	2133015.0	1	1	0	4.10	244	18966
>tr|A0A5F4CCD0|A0A5F4CCD0_CANLF Cysteine rich secretory protein 2 OS=Canis lupus familiaris OX=9615 GN=CRISP2 PE=3 SV=1	4.59	4.51	438.50	2128551.2	5	1	0	4.82	311	11017
>tr|F1Q1L4|F1Q1L4_CANLF Chromosome 6 C16orf89 homolog OS=Canis lupus familiaris OX=9615 GN=C6H16orf89 PE=4 SV=2	5.11	3.85	254.90	2081809.0	2	2	0	8.31	373	20319
>tr|F1PEN8|F1PEN8_CANLF Carboxylic ester hydrolase OS=Canis lupus familiaris OX=9615 GN=BCHE PE=3 SV=1	1.07	1.07	192.20	2070592.3	1	1	0	1.33	602	4179
>tr|A0A5F4CKD5|A0A5F4CKD5_CANLF Polypeptide N-acetylgalactosaminyltransferase OS=Canis lupus familiaris OX=9615 GN=GALNT6 PE=3 SV=1	0.91	0.91	195.10	2068815.9	1	1	0	4.97	644	1617
>tr|J9NRX4|J9NRX4_CANLF Glutathione transferase OS=Canis lupus familiaris OX=9615 GN=LOC481841 PE=3 SV=2	1.57	1.57	302.90	2041225.5	1	1	0	10.98	246	15581
>tr|A0A5F4BT11|A0A5F4BT11_CANLF Ecdysoneless cell cycle regulator OS=Canis lupus familiaris OX=9615 GN=ECD PE=4 SV=1	0.10	0.10	99.30	2034336.8	1	1	0	1.52	656	8613
>tr|A0A5F4CKD5|A0A5F4CKD5_CANLF Polypeptide N-acetylgalactosaminyltransferase OS=Canis lupus familiaris OX=9615 GN=GALNT6 PE=3 SV=1	1.43	1.43	304.30	2021793.5	1	1	0	4.97	644	1617
>tr|A0A5F4CS14|A0A5F4CS14_CANLF Guanylate cyclase OS=Canis lupus familiaris OX=9615 GN=GUCY1A2 PE=3 SV=1	0.10	0.04	128.50	1996180.5	2	1	0	1.03	773	1560
>sp|O46607|GPX5_CANLF Epididymal secretory glutathione peroxidase OS=Canis lupus familiaris OX=9615 GN=GPX5 PE=2 SV=1	0.97	0.91	385.10	1993964.0	4	1	0	6.79	221	564
>tr|J9P3D0|J9P3D0_CANLF Solute carrier family 4 member 9 OS=Canis lupus familiaris OX=9615 GN=SLC4A9 PE=3 SV=2	0.16	0.16	100.90	1970303.4	1	1	0	1.12	893	31921
>tr|J9P8X1|J9P8X1_CANLF Exportin 5 OS=Canis lupus familiaris OX=9615 GN=XPO5 PE=4 SV=1	0.63	0.63	177.70	1935986.6	1	1	0	3.42	1345	1738

>tr|A0A5F4C3Q4|A0A5F4C3Q4_CANLF Polypyrimidine tract binding protein 2 OS=Canis lupus familiaris OX=9615 GN=PTBP2 PE=4 SV=1	0.87	0.87	166.70	1865265.3	1	1	0	2.25	356	10982
>tr|F1Q021|F1Q021_CANLF Family with sequence similarity 160 member A1 OS=Canis lupus familiaris OX=9615 GN=FAM160A1 PE=3 SV=3	0.10	0.04	126.70	1805815.1	1	1	0	1.73	1040	41948
>tr|F1PUY8|F1PUY8_CANLF Anoctamin OS=Canis lupus familiaris OX=9615 GN=ANO7 PE=3 SV=3	1.12	1.12	82.60	1769223.1	1	1	0	0.92	865	40808
>tr|A0A5F4CKD5|A0A5F4CKD5_CANLF Polypeptide N-acetylgalactosaminyltransferase OS=Canis lupus familiaris OX=9615 GN=GALNT6 PE=3 SV=1	1.01	1.01	231.30	1763524.0	1	1	0	4.97	644	1617
>tr|F1PIZ1|F1PIZ1_CANLF Caspase recruitment domain family member 6 OS=Canis lupus familiaris OX=9615 GN=CARD6 PE=4 SV=3	0.10	0.10	57.30	1762056.6	1	1	0	0.65	1071	14260
>tr|F1PB68|F1PB68_CANLF Olfactomedin 4 OS=Canis lupus familiaris OX=9615 GN=OLFM4 PE=4 SV=3	3.45	3.41	381.60	1678911.3	3	1	0	4.40	477	17246
>tr|A0A5F4CQS4|A0A5F4CQS4_CANLF von Willebrand factor A domain containing 1 OS=Canis lupus familiaris OX=9615 GN=VWA1 PE=4 SV=1	4.29	4.27	258.00	1675401.4	2	1	0	3.95	456	3829
>tr|F1Q074|F1Q074_CANLF A-kinase anchoring protein 13 OS=Canis lupus familiaris OX=9615 GN=AKAP13 PE=4 SV=3	0.87	0.82	190.60	1661255.8	4	1	0	0.29	2781	14817
>tr|E2REZ4|E2REZ4_CANLF Coiled-coil and C2 domain containing 2A OS=Canis lupus familiaris OX=9615 GN=CC2D2A PE=4 SV=3	0.10	0.02	231.80	1661127.2	3	1	0	0.76	1585	29419
>tr|A0A5F4D032|A0A5F4D032_CANLF ATP binding cassette subfamily C member 11 OS=Canis lupus familiaris OX=9615 GN=ABCC11 PE=4 SV=1	0.31	0.30	136.50	1638436.4	2	1	0	0.58	1372	7749
>tr|F1PUY8|F1PUY8_CANLF Anoctamin OS=Canis lupus familiaris OX=9615 GN=ANO7 PE=3 SV=3	0.35	0.36	66.50	1626138.0	1	1	0	0.92	865	40808
>tr|J9NS29|J9NS29_CANLF Cystatin domain-containing protein OS=Canis lupus familiaris OX=9615 GN=LOC607874 PE=4 SV=2	1.60	1.60	230.10	1602615.8	1	1	0	4.79	313	30016
>sp|Q6AW47|EST5A_CANLF Carboxylesterase 5A OS=Canis lupus familiaris OX=9615 GN=CES5A PE=2 SV=1	3.31	3.29	402.70	1587805.2	2	1	0	3.65	575	629
>tr|E2QUV3|E2QUV3_CANLF Alpha-2-HS-glycoprotein OS=Canis lupus familiaris OX=9615 GN=AHSG PE=4 SV=2	1.18	1.16	241.50	1533431.5	2	1	0	8.49	365	20747
>sp|O18840|ACTB_CANLF Actin. cytoplasmic 1 OS=Canis lupus familiaris OX=9615 GN=ACTB PE=2 SV=3	1.70	1.02	226.20	1527815.7	3	2	0	8.27	375	642
>tr|E2RN70|E2RN70_CANLF Periphilin 1 OS=Canis lupus familiaris OX=9615 GN=PPHLN1 PE=4 SV=3	0.25	0.25	94.80	1526019.1	1	1	0	1.92	416	41626
>tr|E2R740|E2R740_CANLF AMP deaminase OS=Canis lupus familiaris OX=9615 GN=AMPD3 PE=3 SV=2	0.14	0.14	93.50	1509846.9	1	1	0	3.99	777	24146
>tr|J9P540|J9P540_CANLF Glyceraldehyde-3-phosphate dehydrogenase OS=Canis lupus familiaris OX=9615 GN=LOC477441 PE=3 SV=1	0.83	0.83	176.20	1459388.0	1	1	0	7.19	334	13273
>tr|A0A5F4CPK4|A0A5F4CPK4_CANLF Formin binding protein 1 OS=Canis lupus familiaris OX=9615 GN=FNBP1 PE=4 SV=1	1.19	1.20	105.70	1430119.3	1	1	0	1.42	562	14071
>tr|E2R5P5|E2R5P5_CANLF Calcyclin OS=Canis lupus familiaris OX=9615 GN=S100A8 PE=4 SV=2	0.99	0.99	333.20	1371921.0	1	1	0	14.66	191	27843
>tr|A0A5F4CDM1|A0A5F4CDM1_CANLF L-lactate dehydrogenase OS=Canis lupus familiaris OX=9615 PE=3 SV=1	0.75	0.75	141.70	1346833.8	1	1	0	2.87	279	43073
>tr|F1PX57|F1PX57_CANLF 26S proteasome non-ATPase regulatory subunit 1 OS=Canis lupus familiaris OX=9615 GN=PSMD1 PE=3 SV=3	0.25	0.23	77.40	1340730.7	2	1	0	0.84	953	11564

>tr|A0A5F4CDM1|A0A5F4CDM1_CANLF L-lactate dehydrogenase OS=Canis lupus familiaris OX=9615 PE=3 SV=1	0.47	0.47	128.90	1340687.1	1	1	0	2.87	279	43073
>tr|E2R5F1|E2R5F1_CANLF Exocyst complex component 8 OS=Canis lupus familiaris OX=9615 GN=EXOC8 PE=3 SV=1	0.64	0.64	54.00	1328079.5	1	1	0	2.20	727	7157
>tr|F1P9C4|F1P9C4_CANLF Alpha-mannosidase OS=Canis lupus familiaris OX=9615 GN=MAN2A1 PE=3 SV=3	2.38	2.39	325.70	1326348.2	1	1	0	2.62	1106	11653
>tr|E2R8Z9|E2R8Z9_CANLF Glutathione S-transferase C-terminal domain-containing protein OS=Canis lupus familiaris OX=9615 GN=GSTCD PE=3 SV=1	0.49	0.47	279.40	1301258.4	2	1	0	3.66	629	6707
>tr|E2RGI8|E2RGI8_CANLF Bestrophin OS=Canis lupus familiaris OX=9615 GN=BEST4 PE=3 SV=1	0.12	0.12	90.70	1296156.2	1	1	0	3.59	473	27005
>tr|F2Z4Q6|F2Z4Q6_CANLF Alpha fetoprotein OS=Canis lupus familiaris OX=9615 GN=AFP PE=4 SV=2	2.54	2.54	268.90	1293047.1	1	1	0	2.04	637	24990
>tr|A0A5F4D662|A0A5F4D662_CANLF Par-3 family cell polarity regulator beta OS=Canis lupus familiaris OX=9615 GN=PARD3B PE=3 SV=1	0.25	0.25	132.70	1285599.9	1	1	0	0.70	1142	8202
>tr|F1PCE5|F1PCE5_CANLF Serpin family A member 1 OS=Canis lupus familiaris OX=9615 GN=SERPINA1 PE=3 SV=3	2.50	1.56	245.80	1281937.6	2	2	0	5.46	421	8323
>tr|A0A5F4CXJ3|A0A5F4CXJ3_CANLF Neurofibromin 1 OS=Canis lupus familiaris OX=9615 GN=NF1 PE=4 SV=1	0.49	0.49	178.70	1237785.6	1	1	0	1.79	2733	32406
>tr|J9NSV2|J9NSV2_CANLF Olfactory receptor OS=Canis lupus familiaris OX=9615 GN=OR5AC27 PE=3 SV=2	0.10	0.06	47.60	1236988.8	1	1	0	5.88	306	42329
>sp|Q9XS65|PTGDS_CANLF Prostaglandin-H2 D-isomerase OS=Canis lupus familiaris OX=9615 GN=PTGDS PE=2 SV=1	2.52	2.44	301.70	1199427.4	5	1	0	8.90	191	165
>tr|J9P434|J9P434_CANLF Myotubularin related protein 14 OS=Canis lupus familiaris OX=9615 GN=MTMR14 PE=4 SV=2	0.90	0.90	87.80	1154158.4	1	1	0	1.51	596	17413
>sp|Q8WNN6|SODC_CANLF Superoxide dismutase [Cu-Zn] OS=Canis lupus familiaris OX=9615 GN=SOD1 PE=2 SV=1	1.05	1.05	145.10	1110661.1	1	1	0	15.69	153	6
>tr|F1PFF2|F1PFF2_CANLF Protein S100 OS=Canis lupus familiaris OX=9615 GN=S100A11 PE=3 SV=3	0.10	0.06	162.00	1108493.4	1	1	1	10.48	105	36095
>sp|Q9TT90|ANDR_CANLF Androgen receptor OS=Canis lupus familiaris OX=9615 GN=AR PE=2 SV=1	0.34	0.34	30.30	1107220.0	1	1	0	0.33	907	397
>tr|F1PBI5|F1PBI5_CANLF CUB and Sushi multiple domains 1 OS=Canis lupus familiaris OX=9615 GN=CSMD1 PE=4 SV=3	0.24	0.24	118.60	1103722.4	1	1	0	0.57	3535	25631
>tr|F1PW86|F1PW86_CANLF Inositol 1.4.5-trisphosphate receptor type 1 OS=Canis lupus familiaris OX=9615 GN=ITPR1 PE=3 SV=3	0.10	0.09	58.80	1082468.2	1	1	1	0.76	2628	4403
>tr|A0A5F4C9R4|A0A5F4C9R4_CANLF Integrin subunit alpha V OS=Canis lupus familiaris OX=9615 GN=ITGAV PE=3 SV=1	0.10	0.07	85.60	1077738.8	2	1	0	1.45	1033	3280
>tr|F6PLX8|F6PLX8_CANLF Beta-defensin OS=Canis lupus familiaris OX=9615 GN=DEFB118 PE=3 SV=1	2.25	2.23	172.10	1059339.8	2	1	0	8.77	171	34287
>tr|F6XVF5|F6XVF5_CANLF WD repeat domain 72 OS=Canis lupus familiaris OX=9615 GN=WDR72 PE=4 SV=2	0.88	0.88	136.90	1059203.5	1	1	0	0.75	1071	16644
>sp|P49822|ALBU_CANLF Albumin OS=Canis lupus familiaris OX=9615 GN=ALB PE=1 SV=3	2.27	2.27	311.80	1049389.2	1	1	0	3.45	608	490
>tr|A0A5F4C6B5|A0A5F4C6B5_CANLF Plastin 3 OS=Canis lupus familiaris OX=9615 GN=PLS3 PE=4 SV=1	1.12	1.12	260.60	1045936.4	1	1	0	4.07	639	30730

>tr|F1PDB9|F1PDB9_CANLF Toll like receptor 4 OS=Canis lupus familiaris OX=9615 GN=TLR4 PE=3 SV=2	0.10	0.10	102.60	1028130.0	1	1	0	0.96	833	36972
>tr|A0A5F4D9R9|A0A5F4D9R9_CANLF Phosphoribosyl pyrophosphate synthetase 1 like 1 OS=Canis lupus familiaris OX=9615 GN=PRPS1L1 PE=3 SV=1	0.80	0.80	141.10	1027846.7	1	1	0	6.72	268	31528
>tr|E2RG75|E2RG75_CANLF Inactive ribonuclease-like protein 9 OS=Canis lupus familiaris OX=9615 GN=RNASE9 PE=3 SV=2	3.07	3.05	289.30	1011133.2	2	1	0	7.07	198	41734
>tr|A0A5F4D654|A0A5F4D654_CANLF Nucleotide binding oligomerization domain containing 2 OS=Canis lupus familiaris OX=9615 GN=NOD2 PE=4 SV=1	0.21	0.21	123.50	973418.0	1	1	0	2.09	957	2067
>tr|A0A5F4DA59|A0A5F4DA59_CANLF Cystatin B OS=Canis lupus familiaris OX=9615 GN=CSTB PE=3 SV=1	0.67	0.65	178.20	960092.3	2	1	0	12.50	96	8919
>tr|A0A5F4CKD5|A0A5F4CKD5_CANLF Polypeptide N-acetylgalactosaminyltransferase OS=Canis lupus familiaris OX=9615 GN=GALNT6 PE=3 SV=1	2.13	2.13	271.60	935901.4	1	1	0	4.97	644	1617
>tr|E2RGC9|E2RGC9_CANLF Target of EGR1. exonuclease OS=Canis lupus familiaris OX=9615 GN=TOE1 PE=3 SV=1	0.24	0.24	90.40	885958.7	1	1	0	1.57	510	36568
>tr|A0A5F4CPK4|A0A5F4CPK4_CANLF Formin binding protein 1 OS=Canis lupus familiaris OX=9615 GN=FNBP1 PE=4 SV=1	0.10	0.09	99.30	856436.5	1	1	0	1.42	562	14071
>tr|A0A5F4BVH6|A0A5F4BVH6_CANLF KIAA1549 OS=Canis lupus familiaris OX=9615 GN=KIAA1549 PE=4 SV=1	0.60	0.59	196.60	851657.8	2	1	0	0.50	1793	5440
>tr|A0A5F4D662|A0A5F4D662_CANLF Par-3 family cell polarity regulator beta OS=Canis lupus familiaris OX=9615 GN=PARD3B PE=3 SV=1	0.13	0.13	111.60	838803.2	1	1	0	0.70	1142	8202
>tr|A0A5F4C3M5|A0A5F4C3M5_CANLF IQ motif containing GTPase activating protein 2 OS=Canis lupus familiaris OX=9615 GN=IQGAP2 PE=4 SV=1	0.11	0.11	95.20	834096.3	1	1	0	0.51	1577	1056
>tr|E2R6E0|E2R6E0_CANLF Lipocln_cytosolic_FA-bd_dom domain-containing protein OS=Canis lupus familiaris OX=9615 GN=LCNL1 PE=3 SV=2	0.97	0.91	267.20	830526.5	4	1	0	3.68	299	1932
>tr|E2RGF6|E2RGF6_CANLF WD repeat and HMG-box DNA binding protein 1 OS=Canis lupus familiaris OX=9615 GN=WDHD1 PE=4 SV=3	0.10	0.09	60.50	824042.5	1	1	0	0.73	1103	21537
>tr|A0A5F4DE62|A0A5F4DE62_CANLF Ataxin 2 like OS=Canis lupus familiaris OX=9615 GN=ATXN2L PE=3 SV=1	0.81	0.81	107.50	813562.7	1	1	0	0.90	1005	12591
>tr|E2RSH8|E2RSH8_CANLF Tyrosine-protein kinase OS=Canis lupus familiaris OX=9615 GN=SRC PE=3 SV=2	0.21	0.21	124.70	805266.1	1	1	1	8.40	536	23529
>tr|A0A5F4CS14|A0A5F4CS14_CANLF Guanylate cyclase OS=Canis lupus familiaris OX=9615 GN=GUCY1A2 PE=3 SV=1	0.18	0.18	128.20	788052.7	1	1	0	1.03	773	1560
>tr|E2R6E0|E2R6E0_CANLF Lipocln_cytosolic_FA-bd_dom domain-containing protein OS=Canis lupus familiaris OX=9615 GN=LCNL1 PE=3 SV=2	1.23	1.21	202.40	774560.2	2	1	0	3.68	299	1932
>tr|F1PRT7|F1PRT7_CANLF Cation-transporting ATPase OS=Canis lupus familiaris OX=9615 GN=ATP13A4 PE=3 SV=2	0.44	0.44	112.80	770209.0	1	1	0	0.67	1197	1348
>tr|F1PNW0|F1PNW0_CANLF PMS1 homolog 2. mismatch repair system component OS=Canis lupus familiaris OX=9615 GN=PMS2 PE=3 SV=3	0.28	0.28	69.00	759546.8	1	1	0	1.45	897	14263
>tr|E2R6E0|E2R6E0_CANLF Lipocln_cytosolic_FA-bd_dom domain-containing protein OS=Canis lupus familiaris OX=9615 GN=LCNL1 PE=3 SV=2	2.67	2.61	320.40	757271.8	4	1	0	3.68	299	1932

>tr|F1PTW3|F1PTW3_CANLF KIAA0753 OS=Canis lupus familiaris OX=9615 GN=KIAA0753 PE=4 SV=3	0.49	0.49	151.00	739731.3	1	1	0	0.88	905	33899
>tr|E2QSN3|E2QSN3_CANLF Teashirt zinc finger homeobox 1 OS=Canis lupus familiaris OX=9615 GN=TSHZ1 PE=3 SV=2	0.95	0.95	75.20	735263.0	1	1	0	1.82	1098	44714
>tr|A0A5F4CPK4|A0A5F4CPK4_CANLF Formin binding protein 1 OS=Canis lupus familiaris OX=9615 GN=FNBP1 PE=4 SV=1	0.10	0.08	85.40	700779.6	1	1	0	1.42	562	14071
>tr|E2R8Z9|E2R8Z9_CANLF Glutathione S-transferase C-terminal domain-containing protein OS=Canis lupus familiaris OX=9615 GN=GSTCD PE=3 SV=1	0.28	0.26	123.70	691489.8	2	1	0	3.66	629	6707
>tr|F1PF06|F1PF06_CANLF Sodium channel protein OS=Canis lupus familiaris OX=9615 GN=SCN7A PE=3 SV=3	0.29	0.25	94.80	662161.6	3	1	0	0.36	1677	19116
>tr|E2RG75|E2RG75_CANLF Inactive ribonuclease-like protein 9 OS=Canis lupus familiaris OX=9615 GN=RNASE9 PE=3 SV=2	1.78	1.74	267.60	655679.1	3	1	0	7.07	198	41734
>sp|Q95168|ZO2_CANLF Tight junction protein ZO-2 OS=Canis lupus familiaris OX=9615 GN=TJP2 PE=1 SV=1	0.73	0.73	118.70	650746.5	1	1	1	1.87	1174	13
>tr|F6UVH0|F6UVH0_CANLF WD_REPEATS_REGION domain-containing protein OS=Canis lupus familiaris OX=9615 GN=WDR77 PE=4 SV=2	0.10	0.02	78.40	624851.4	2	1	0	2.74	475	26525
>tr|E2RD46|E2RD46_CANLF Apolipoprotein B mRNA editing enzyme catalytic polypeptide like 4 OS=Canis lupus familiaris OX=9615 GN=APOBEC4 PE=4 SV=2	0.22	0.22	123.30	619578.0	1	1	0	8.12	357	31853
>sp|P25473|CLUS_CANLF Clusterin OS=Canis lupus familiaris OX=9615 GN=CLU PE=2 SV=1	0.81	0.81	206.60	617120.7	1	1	0	2.70	445	725
>tr|F1PLT8|F1PLT8_CANLF Sulfhydryl oxidase OS=Canis lupus familiaris OX=9615 GN=QSOX1 PE=3 SV=3	2.07	2.07	265.00	617115.5	1	1	0	3.35	568	33056
>tr|A0A5F4D8W3|A0A5F4D8W3_CANLF Vitamin K-dependent protein S OS=Canis lupus familiaris OX=9615 GN=PROS1 PE=4 SV=1	0.48	0.42	34.40	603845.3	4	1	0	1.58	505	1354
>tr|A0A5F4D6X1|A0A5F4D6X1_CANLF Cysteine rich secretory protein 2 OS=Canis lupus familiaris OX=9615 GN=CRISP2 PE=3 SV=1	0.43	0.43	201.10	602729.8	1	1	1	5.30	321	3538
>tr|A0A5F4D4G2|A0A5F4D4G2_CANLF LLGL scribble cell polarity complex component 1 OS=Canis lupus familiaris OX=9615 GN=LLGL1 PE=3 SV=1	0.25	0.25	66.90	580727.6	1	1	0	0.52	1159	7946
>tr|A0A5F4DHV7|A0A5F4DHV7_CANLF Helicase with zinc finger OS=Canis lupus familiaris OX=9615 GN=HELZ PE=4 SV=1	0.51	0.51	113.80	574010.6	1	1	0	1.12	1424	33012
>tr|E2RPJ3|E2RPJ3_CANLF Serine/threonine-protein kinase RIO3 OS=Canis lupus familiaris OX=9615 GN=RIOK3 PE=3 SV=2	0.79	0.79	161.10	567930.3	1	1	0	4.43	519	16472
>tr|A0A5F4BQT1|A0A5F4BQT1_CANLF YEATS domain containing 2 OS=Canis lupus familiaris OX=9615 GN=YEATS2 PE=4 SV=1	0.10	0.11	106.00	554184.8	1	1	1	1.73	1326	5342
>tr|E2QX17|E2QX17_CANLF Proteasome subunit beta OS=Canis lupus familiaris OX=9615 GN=PSMB3 PE=3 SV=1	0.27	0.27	70.50	549481.2	1	1	1	17.07	205	13730
>tr|A0A5F4DCP3|A0A5F4DCP3_CANLF Centrobin. centriole duplication and spindle assembly protein OS=Canis lupus familiaris OX=9615 GN=CNTROB PE=4 SV=1	0.27	0.27	50.50	537471.3	1	1	1	2.33	858	1454
>tr|A0A5F4CLU1|A0A5F4CLU1_CANLF Superoxide dismutase [Cu-Zn] OS=Canis lupus familiaris OX=9615 GN=SOD1 PE=3 SV=1	0.83	0.83	141.50	529059.9	1	1	0	7.09	141	25417
>tr|F1PUC4|F1PUC4_CANLF Olfactory receptor OS=Canis lupus familiaris OX=9615 GN=OR51T1 PE=3 SV=3	0.31	0.31	159.00	526689.9	1	1	0	2.45	327	41557

>tr|F1PS03|F1PS03_CANLF 1-phosphatidylinositol 4.5-bisphosphate phosphodiesterase gamma OS=Canis lupus familiaris OX=9615 GN=PLCG1 PE=4 SV=2	0.13	0.13	130.30	524786.1	1	1	0	1.23	1217	6493
>tr|A0A5F4DEV4|A0A5F4DEV4_CANLF Calmodulin 1 OS=Canis lupus familiaris OX=9615 GN=CALM1 PE=4 SV=1	0.51	0.51	138.70	524387.4	1	1	0	9.09	187	2591
>tr|A0A5F4D4G2|A0A5F4D4G2_CANLF LLGL scribble cell polarity complex component 1 OS=Canis lupus familiaris OX=9615 GN=LLGL1 PE=3 SV=1	0.10	0.04	86.00	514249.6	1	1	0	0.52	1159	7946
>tr|F1PRT7|F1PRT7_CANLF Cation-transporting ATPase OS=Canis lupus familiaris OX=9615 GN=ATP13A4 PE=3 SV=2	1.12	1.12	96.90	513919.2	1	1	0	0.67	1197	1348
>tr|E2R6E0|E2R6E0_CANLF Lipocln_cytosolic_FA-bd_dom domain-containing protein OS=Canis lupus familiaris OX=9615 GN=LCNL1 PE=3 SV=2	0.78	0.76	272.40	503244.9	2	1	0	3.68	299	1932
>tr|E2R472|E2R472_CANLF Acyl-coenzyme A oxidase OS=Canis lupus familiaris OX=9615 GN=ACOX2 PE=3 SV=2	0.52	0.52	125.30	496063.9	1	1	0	1.47	681	42004
>sp|Q9GL25|ESPB1_CANLF Epididymal sperm-binding protein 1 OS=Canis lupus familiaris OX=9615 GN=ELSPBP1 PE=1 SV=1	0.60	0.60	125.90	487682.0	1	1	0	6.94	245	36
>tr|F1P744|F1P744_CANLF Chondroitin sulfate proteoglycan 5 OS=Canis lupus familiaris OX=9615 GN=CSPG5 PE=4 SV=2	0.95	0.95	183.00	471758.0	1	1	0	4.74	401	18644
>tr|A0A5F4CFB6|A0A5F4CFB6_CANLF Potassium voltage-gated channel interacting protein 3 OS=Canis lupus familiaris OX=9615 GN=KCNIP3 PE=3 SV=1	0.49	0.49	101.90	467339.8	1	1	0	1.84	708	2187
>tr|F6XIT9|F6XIT9_CANLF Alpha kinase 2 OS=Canis lupus familiaris OX=9615 GN=ALPK2 PE=4 SV=1	0.51	0.52	108.80	458434.5	1	1	0	0.95	2115	6659
>tr|E2R840|E2R840_CANLF Olfactory receptor OS=Canis lupus familiaris OX=9615 GN=OR4E2 PE=3 SV=2	0.52	0.52	37.90	456403.9	1	1	0	4.22	308	8491
>sp|P25473|CLUS_CANLF Clusterin OS=Canis lupus familiaris OX=9615 GN=CLU PE=2 SV=1	0.26	0.26	158.40	450233.4	1	1	0	2.70	445	725
>tr|F6UZ83|F6UZ83_CANLF HscB mitochondrial iron-sulfur cluster cochaperone OS=Canis lupus familiaris OX=9615 GN=HSCB PE=3 SV=1	0.81	0.81	87.40	421146.5	1	1	0	3.40	235	2614
>tr|A0A5F4CCD0|A0A5F4CCD0_CANLF Cysteine rich secretory protein 2 OS=Canis lupus familiaris OX=9615 GN=CRISP2 PE=3 SV=1	2.73	2.73	270.40	393124.7	1	1	0	4.82	311	11017
>tr|E2QYJ9|E2QYJ9_CANLF Diphosphomevalonate decarboxylase OS=Canis lupus familiaris OX=9615 GN=MVD PE=3 SV=1	0.20	0.20	56.90	377243.6	1	1	0	5.25	400	43817
>tr|A0A5F4CCJ1|A0A5F4CCJ1_CANLF Transmembrane protein 241 OS=Canis lupus familiaris OX=9615 GN=TMEM241 PE=4 SV=1	0.91	0.91	239.00	363065.5	1	1	0	3.75	347	27419
>tr|A0A5F4DFG2|A0A5F4DFG2_CANLF SH3 domain and tetratricopeptide repeats 1 OS=Canis lupus familiaris OX=9615 GN=SH3TC1 PE=4 SV=1	0.16	0.16	53.70	360435.6	1	1	1	1.30	1463	23163
>tr|A0A5F4BUE2|A0A5F4BUE2_CANLF SpoU_methylase domain-containing protein OS=Canis lupus familiaris OX=9615 GN=TARBP1 PE=4 SV=1	0.93	0.93	107.00	354581.1	1	1	0	0.50	1602	2815
>tr|J9NRL7|J9NRL7_CANLF Phosphatidylinositol-3.4.5-trisphosphate dependent Rac exchange factor 1 OS=Canis lupus familiaris OX=9615 GN=PREX1 PE=4 SV=2	0.31	0.31	119.70	347874.9	1	1	0	1.32	1585	13450
>tr|A0A5F4D4V6|A0A5F4D4V6_CANLF Ankyrin repeat domain 52 OS=Canis lupus familiaris OX=9615 GN=ANKRD52 PE=4 SV=1	0.10	0.09	140.80	347283.3	1	1	0	1.87	1067	6228
>tr|F1PG67|F1PG67_CANLF Myelin transcription factor 1 OS=Canis lupus familiaris OX=9615 GN=MYT1 PE=3 SV=3	0.25	0.24	76.00	343529.7	2	1	0	1.29	1166	1094

>tr|E2REQ4|E2REQ4_CANLF NIPA like domain containing 3 OS=Canis lupus familiaris OX=9615 GN=NIPAL3 PE=3 SV=3	0.79	0.79	95.50	340565.2	1	1	0	1.90	368	39451
>tr|L7N0I7|L7N0I7_CANLF Tubulin beta chain OS=Canis lupus familiaris OX=9615 GN=TUBB4B PE=3 SV=1	1.37	1.37	207.60	338150.6	1	1	0	6.07	445	2921
>tr|J9P9I8|J9P9I8_CANLF Chromosome 20 C3orf22 homolog OS=Canis lupus familiaris OX=9615 GN=C20H3orf22 PE=4 SV=2	0.27	0.27	23.40	331690.1	1	1	1	5.80	276	21775
>tr|F1PVA2|F1PVA2_CANLF Adhesion G protein-coupled receptor V1 OS=Canis lupus familiaris OX=9615 GN=ADGRV1 PE=4 SV=3	0.10	0.04	99.80	316673.7	1	1	1	0.22	6300	19804
>tr|F1PVU3|F1PVU3_CANLF Non-specific serine/threonine protein kinase OS=Canis lupus familiaris OX=9615 GN=MARK2 PE=3 SV=3	1.05	1.05	116.00	309933.1	1	1	0	0.98	813	14910
>tr|A0A5F4CNW2|A0A5F4CNW2_CANLF Radical S-adenosyl methionine and flavodoxin domain-containing protein 1 OS=Canis lupus familiaris OX=9615 GN=LOC479708 PE=3 SV=1	0.14	0.14	119.80	304090.8	1	1	1	1.04	670	13683
>tr|F1Q0W5|F1Q0W5_CANLF Interleukin 17 receptor D OS=Canis lupus familiaris OX=9615 GN=IL17RD PE=4 SV=3	0.10	0.11	61.70	291910.2	1	1	0	2.19	594	34031
>tr|Q2Z1P8|Q2Z1P8_CANLF Anion exchange protein OS=Canis lupus familiaris OX=9615 GN=SLC4A1 PE=2 SV=1	0.28	0.28	90.00	282877.5	1	1	0	1.08	930	41370
>tr|E2RJS2|E2RJS2_CANLF Solute carrier family 27 member 1 OS=Canis lupus familiaris OX=9615 GN=SLC27A1 PE=3 SV=1	0.10	0.06	91.40	280942.1	1	1	0	2.01	646	3080
>tr|F1PZI2|F1PZI2_CANLF RING-type E3 ubiquitin transferase OS=Canis lupus familiaris OX=9615 GN=MIB2 PE=4 SV=3	0.95	0.95	148.10	270721.4	1	1	0	1.19	1005	7737
>tr|A0A5F4CX38|A0A5F4CX38_CANLF Neurobeachin like 2 OS=Canis lupus familiaris OX=9615 GN=NBEAL2 PE=3 SV=1	0.10	0.05	32.30	260456.3	1	1	1	0.67	2521	10261
>tr|F1PS03|F1PS03_CANLF 1-phosphatidylinositol 4.5-bisphosphate phosphodiesterase gamma OS=Canis lupus familiaris OX=9615 GN=PLCG1 PE=4 SV=2	0.82	0.82	44.30	260383.5	1	1	0	1.23	1217	6493
>tr|A0A5F4D1W5|A0A5F4D1W5_CANLF PITPNM family member 3 OS=Canis lupus familiaris OX=9615 GN=PITPNM3 PE=3 SV=1	0.95	0.95	87.80	241359.5	1	1	0	0.98	918	2286
>tr|A0A5F4CMG4|A0A5F4CMG4_CANLF Family with sequence similarity 114 member A2 OS=Canis lupus familiaris OX=9615 GN=FAM114A2 PE=3 SV=1	0.17	0.17	89.20	239104.5	1	1	0	3.44	436	7934
>tr|J9NZ15|J9NZ15_CANLF Cadherin EGF LAG seven-pass G-type receptor 3 OS=Canis lupus familiaris OX=9615 GN=CELSR3 PE=3 SV=2	0.10	0.10	65.70	231059.4	1	1	1	0.59	2546	1516
>tr|F1PZI2|F1PZI2_CANLF RING-type E3 ubiquitin transferase OS=Canis lupus familiaris OX=9615 GN=MIB2 PE=4 SV=3	0.44	0.44	135.70	229703.4	1	1	0	1.19	1005	7737
>tr|A0A5F4BUL6|A0A5F4BUL6_CANLF Piezo-type mechanosensitive ion channel component OS=Canis lupus familiaris OX=9615 GN=PIEZO2 PE=3 SV=1	0.47	0.47	92.90	222554.0	1	1	0	0.33	2696	1427
>sp|P49822|ALBU_CANLF Albumin OS=Canis lupus familiaris OX=9615 GN=ALB PE=1 SV=3	0.25	0.25	81.00	222048.7	1	1	0	1.97	608	490
>tr|A0A5F4CCJ1|A0A5F4CCJ1_CANLF Transmembrane protein 241 OS=Canis lupus familiaris OX=9615 GN=TMEM241 PE=4 SV=1	0.41	0.41	147.40	215163.6	1	1	0	3.75	347	27419
>tr|F1PE24|F1PE24_CANLF SRF-dependent transcription regulation-associated protein OS=Canis lupus familiaris OX=9615 GN=SRFBP1 PE=4 SV=3	0.71	0.71	57.00	214069.9	1	1	0	3.27	428	19920
>tr|A0A5F4DGF5|A0A5F4DGF5_CANLF Alkaline phosphatase OS=Canis lupus familiaris OX=9615 GN=ALPL PE=3 SV=1	0.74	0.74	199.20	213265.5	1	1	0	1.75	572	6357

>tr|F1Q2F6|F1Q2F6_CANLF 3-hydroxyacyl-[acyl-carrier-protein] dehydratase OS=Canis lupus familiaris OX=9615 GN=FASN PE=4 SV=3	0.10	0.10	32.40	210432.3	1	1	0	0.52	2478	22936
>tr|F1PXQ6|F1PXQ6_CANLF Polypeptide N-acetylgalactosaminyltransferase OS=Canis lupus familiaris OX=9615 GN=GALNT17 PE=3 SV=2	0.38	0.38	145.70	203405.9	1	1	0	1.34	598	6799
>tr|F1PZI2|F1PZI2_CANLF RING-type E3 ubiquitin transferase OS=Canis lupus familiaris OX=9615 GN=MIB2 PE=4 SV=3	1.18	1.18	132.80	199402.9	1	1	0	1.19	1005	7737
>tr|A0A5F4D7J3|A0A5F4D7J3_CANLF Non-specific serine/threonine protein kinase OS=Canis lupus familiaris OX=9615 GN=CDC42BPA PE=3 SV=1	0.24	0.25	108.80	197697.5	1	1	0	0.50	1794	1069
>tr|A0A5F4BUE2|A0A5F4BUE2_CANLF SpoU_methylase domain-containing protein OS=Canis lupus familiaris OX=9615 GN=TARBP1 PE=4 SV=1	0.95	0.95	56.50	193001.7	1	1	0	0.50	1602	2815
>tr|F1PHM8|F1PHM8_CANLF Hexosyltransferase OS=Canis lupus familiaris OX=9615 GN=B3GNT4 PE=3 SV=2	0.79	0.79	34.60	191931.1	1	1	0	2.24	357	33620
>tr|A0A5F4C9R4|A0A5F4C9R4_CANLF Integrin subunit alpha V OS=Canis lupus familiaris OX=9615 GN=ITGAV PE=3 SV=1	1.01	0.99	42.80	191599.7	2	1	0	1.45	1033	3280
>tr|E2RJG1|E2RJG1_CANLF Armadillo repeat containing X-linked 3 OS=Canis lupus familiaris OX=9615 GN=ARMCX3 PE=3 SV=2	0.24	0.24	30.90	182099.3	1	1	1	5.54	379	5713
>tr|J9NUK8|J9NUK8_CANLF C-C motif chemokine OS=Canis lupus familiaris OX=9615 GN=CCL22 PE=3 SV=1	0.95	0.95	68.30	180390.9	1	1	0	9.68	93	2965
>tr|E2RL57|E2RL57_CANLF Pre-mRNA-splicing factor SPF27 OS=Canis lupus familiaris OX=9615 GN=BCAS2 PE=3 SV=1	0.14	0.14	79.30	178071.9	1	1	0	6.67	225	29223
>tr|E2RH09|E2RH09_CANLF Small RNA binding exonuclease protection factor La OS=Canis lupus familiaris OX=9615 GN=SSB PE=4 SV=2	0.10	0.07	89.40	176993.6	1	1	0	1.97	406	21679
>tr|F1Q0P9|F1Q0P9_CANLF AT-hook containing transcription factor 1 OS=Canis lupus familiaris OX=9615 GN=AHCTF1 PE=4 SV=3	0.12	0.12	35.00	172283.4	1	1	0	0.39	2321	1521
>tr|J9P2E5|J9P2E5_CANLF PPFIA binding protein 1 OS=Canis lupus familiaris OX=9615 GN=PPFIBP1 PE=3 SV=2	0.35	0.35	50.60	169581.7	1	1	0	1.39	1005	1621
>sp|Q9XSR3|RBM47_CANLF RNA-binding protein 47 OS=Canis lupus familiaris OX=9615 GN=RBM47 PE=2 SV=1	0.52	0.52	55.00	167701.5	1	1	0	1.52	592	166
>tr|J9P3E6|J9P3E6_CANLF Zinc finger protein 250 OS=Canis lupus familiaris OX=9615 GN=ZNF250 PE=4 SV=2	0.10	0.09	38.60	161137.5	1	1	1	2.87	557	9280
>tr|F1PZ92|F1PZ92_CANLF Purinergic receptor P2Y14 OS=Canis lupus familiaris OX=9615 GN=P2RY14 PE=4 SV=3	0.22	0.22	49.30	144065.8	1	1	0	2.35	340	27948
>tr|F1P795|F1P795_CANLF RAS guanyl releasing protein 2 OS=Canis lupus familiaris OX=9615 GN=RASGRP2 PE=3 SV=2	0.25	0.25	6.40	140693.8	1	1	0	1.32	606	41099
>tr|F1PAV7|F1PAV7_CANLF ATP/GTP binding protein like 2 OS=Canis lupus familiaris OX=9615 GN=AGBL2 PE=3 SV=3	1.01	1.01	69.50	139042.3	1	1	0	0.93	863	6782
>tr|A0A5F4CTD7|A0A5F4CTD7_CANLF Acetyl-CoA carboxylase 1 OS=Canis lupus familiaris OX=9615 GN=ACACA PE=4 SV=1	0.45	0.45	40.40	115326.9	1	1	1	0.67	2380	11170
