# Supplementary material for: Proteome Profiling of Canine Epididymal Fluid: In Search of Protein Markers of Epididymal Sperm Motility
Source: Int J Mol Sci. 2023 Sep 30;24(19):14790. doi: 10.3390/ijms241914790 (PMC10573609; doi:10.3390/ijms241914790)
Supplement: Supplementary file 1 [file ijms-24-14790-s001.zip › Supplementary Table S2.pdf]

Supplementary Table S2. Proteins of the cauda epididymal fluid of dogs (*Canis lupus familiaris*) with poor sperm motility (PSM) evaluated by nanoUPLC-Q-TOF/MS.

Description	|Log Prob|	Best |Log Prob|	Best score	Total Intensity	# of spectra	# of unique peptides	# of mod peptides	Coverage %	# AA's in protein	Protein DB number
>tr|F1PR54|F1PR54_CANLF Lactotransferrin OS=Canis lupus familiaris OX=9615 GN=LTF PE=3 SV=1	82,78	6,65	624,50	2903895301,9	136	24	2	33,90	708	40436
>sp|O18840|ACTB_CANLF Actin, cytoplasmic 1 OS=Canis lupus familiaris OX=9615 GN=ACTB PE=2 SV=3	23,21	4,76	463,00	1260422780,2	47	7	0	14,93	375	642
>tr|F1PJ71|F1PJ71_CANLF Glutathione peroxidase OS=Canis lupus familiaris OX=9615 GN=GPX5 PE=3 SV=2	26,18	7,08	645,30	857174949,9	80	7	1	45,25	221	19009
>sp|P25473|CLUS_CANLF Clusterin OS=Canis lupus familiaris OX=9615 GN=CLU PE=2 SV=1	14,78	4,60	459,20	482472376,1	22	9	2	18,20	445	725
>tr|F1PR54|F1PR54_CANLF Lactotransferrin OS=Canis lupus familiaris OX=9615 GN=LTF PE=3 SV=1	70,57	8,44	673,60	345105913,0	86	17	3	32,06	708	40436
>sp|O46607|GPX5_CANLF Epididymal secretory glutathione peroxidase OS=Canis lupus familiaris OX=9615 GN=GPX5 PE=2 SV=1	9,01	4,38	489,60	330057705,9	17	4	0	18,10	221	564
>sp|Q9XS65|PTGDS_CANLF Prostaglandin-H2 D-isomerase OS=Canis lupus familiaris OX=9615 GN=PTGDS PE=2 SV=1	12,26	8,60	658,80	303880413,7	25	3	2	23,56	191	165
>sp|Q28895|NPC2_CANLF NPC intracellular cholesterol transporter 2 OS=Canis lupus familiaris OX=9615 GN=NPC2 PE=2 SV=1	14,28	8,96	712,30	293600777,7	12	2	0	24,16	149	153
>sp|P49822|ALBU_CANLF Albumin OS=Canis lupus familiaris OX=9615 GN=ALB PE=1 SV=3	10,36	4,83	539,70	289532592,1	13	4	0	10,36	608	490
>tr|F1PR54|F1PR54_CANLF Lactotransferrin OS=Canis lupus familiaris OX=9615 GN=LTF PE=3 SV=1	75,34	5,92	641,40	274242371,5	83	22	6	38,70	708	40436
>tr|A0A5F4CCD0|A0A5F4CCD0_CANLF Cysteine rich secretory protein 2 OS=Canis lupus familiaris OX=9615 GN=CRISP2 PE=3 SV=1	8,63	4,27	449,30	247778382,8	9	2	0	10,29	311	11017
>tr|F1PR54|F1PR54_CANLF Lactotransferrin OS=Canis lupus familiaris OX=9615 GN=LTF PE=3 SV=1	51,42	8,08	669,60	229432832,4	76	12	1	22,03	708	40436
>sp|Q6AW47|EST5A_CANLF Carboxylesterase 5A OS=Canis lupus familiaris OX=9615 GN=CES5A PE=2 SV=1	6,06	4,36	469,60	209362731,1	17	2	0	5,74	575	629
>sp|Q9XS65|PTGDS_CANLF Prostaglandin-H2 D-isomerase OS=Canis lupus familiaris OX=9615 GN=PTGDS PE=2 SV=1	8,23	5,30	539,50	208206170,1	13	3	1	39,79	191	165
>sp|Q659K0|CCNB3_CANLF G2/mitotic-specific cyclin-B3 OS=Canis lupus familiaris OX=9615 GN=CCNB3 PE=2 SV=1	1,69	1,41	115,90	194692521,2	15	1	0	0,38	1330	483
>sp|P49822|ALBU_CANLF Albumin OS=Canis lupus familiaris OX=9615 GN=ALB PE=1 SV=3	22,15	5,88	563,90	180272050,6	28	7	2	14,47	608	490
>tr|J9NWY1|J9NWY1_CANLF Quinoid dihydropteridine reductase OS=Canis lupus familiaris OX=9615 GN=QDPR PE=3 SV=2	3,15	3,03	374,50	176250556,5	7	1	0	6,42	296	22710
>tr|A0A5F4DA00|A0A5F4DA00_CANLF G2/mitotic-specific cyclin-B3 OS=Canis lupus familiaris OX=9615 GN=CCNB3 PE=3 SV=1	1,35	0,99	93,80	175048340,4	19	2	0	0,52	1352	45045
>sp|Q28895|NPC2_CANLF NPC intracellular cholesterol transporter 2 OS=Canis lupus familiaris OX=9615 GN=NPC2 PE=2 SV=1	10,99	10,71	776,40	163008295,7	15	1	0	15,44	149	153
>sp|Q9GL25|ESPB1_CANLF Epididymal sperm-binding protein 1	2,96	1,63	351,00	157043247,6	12	2	1	3,27	245	36

OS=Canis lupus familiaris OX=9615 GN=ELSPBP1 PE=1 SV=1										
>tr|F1PB68|F1PB68_CANLF Olfactomedin 4 OS=Canis lupus familiaris OX=9615 GN=OLFM4 PE=4 SV=3	11,20	4,06	342,50	145528720,0	9	3	0	10,69	477	17246
>sp|Q659K0|CCNB3_CANLF G2/mitotic-specific cyclin-B3 OS=Canis lupus familiaris OX=9615 GN=CCNB3 PE=2 SV=1	0,38	0,14	125,40	141875792,7	13	1	0	0,38	1330	483
>tr|J9NVE0|J9NVE0_CANLF KRAB domain-containing protein OS=Canis lupus familiaris OX=9615 GN=LOC606925 PE=4 SV=1	0,19	0,04	125,00	139278712,7	9	1	0	4,08	147	3752
>tr|F1PR54|F1PR54_CANLF Lactotransferrin OS=Canis lupus familiaris OX=9615 GN=LTF PE=3 SV=1	25,42	7,24	659,50	132982807,8	25	8	0	15,96	708	40436
>tr|A0A5F4BVX3|A0A5F4BVX3_CANLF ATP-dependent RNA helicase DHX29 OS=Canis lupus familiaris OX=9615 GN=DHX29 PE=3 SV=1	0,11	0,03	217,40	122017438,6	5	1	0	0,71	1261	11633
>sp|P25473|CLUS_CANLF Clusterin OS=Canis lupus familiaris OX=9615 GN=CLU PE=2 SV=1	26,61	6,39	634,70	119971934,8	37	7	1	22,47	445	725
>tr|F2Z4Q6|F2Z4Q6_CANLF Alpha fetoprotein OS=Canis lupus familiaris OX=9615 GN=AFP PE=4 SV=2	35,14	7,54	664,10	107309933,1	32	10	1	27,63	637	24990
>tr|A0A5F4C1S8|A0A5F4C1S8_CANLF E3 ubiquitin-protein ligase CBL OS=Canis lupus familiaris OX=9615 GN=CBL PE=4 SV=1	0,18	0,08	195,00	106460722,8	6	1	0	0,52	773	1308
>tr|F1PR54|F1PR54_CANLF Lactotransferrin OS=Canis lupus familiaris OX=9615 GN=LTF PE=3 SV=1	51,90	8,52	688,90	101463395,7	67	14	1	25,99	708	40436
>tr|A0A5F4C6B5|A0A5F4C6B5_CANLF Plastin 3 OS=Canis lupus familiaris OX=9615 GN=PLS3 PE=4 SV=1	8,53	4,00	477,70	96569661,8	10	5	0	14,71	639	30730
>sp|Q659K0|CCNB3_CANLF G2/mitotic-specific cyclin-B3 OS=Canis lupus familiaris OX=9615 GN=CCNB3 PE=2 SV=1	0,78	0,44	120,30	94654876,1	18	1	0	0,38	1330	483
>sp|O18840|ACTB_CANLF Actin, cytoplasmic 1 OS=Canis lupus familiaris OX=9615 GN=ACTB PE=2 SV=3	6,63	3,26	432,80	90839807,5	18	3	0	7,20	375	642
>tr|E2RCT1|E2RCT1_CANLF WAP domain-containing protein OS=Canis lupus familiaris OX=9615 PE=4 SV=2	4,14	4,06	366,30	89215392,1	5	1	0	13,79	116	21717
>sp|P49822|ALBU_CANLF Albumin OS=Canis lupus familiaris OX=9615 GN=ALB PE=1 SV=3	18,41	7,01	640,40	88369343,1	19	5	0	13,32	608	490
>tr|F2Z4Q6|F2Z4Q6_CANLF Alpha fetoprotein OS=Canis lupus familiaris OX=9615 GN=AFP PE=4 SV=2	42,27	10,55	744,10	86879158,2	40	7	0	17,90	637	24990
>tr|F1PR54|F1PR54_CANLF Lactotransferrin OS=Canis lupus familiaris OX=9615 GN=LTF PE=3 SV=1	40,79	8,28	673,20	84848416,5	54	12	1	18,93	708	40436
>sp|Q6AW47|EST5A_CANLF Carboxylesterase 5A OS=Canis lupus familiaris OX=9615 GN=CES5A PE=2 SV=1	19,22	11,59	761,30	78741726,5	20	5	0	18,09	575	629
>sp|Q6AW47|EST5A_CANLF Carboxylesterase 5A OS=Canis lupus familiaris OX=9615 GN=CES5A PE=2 SV=1	11,73	5,45	568,00	75449919,4	17	3	0	9,74	575	629
>tr|E2QZK8|E2QZK8_CANLF Potassium channel tetramerization domain containing 13 OS=Canis lupus familiaris OX=9615 GN=KCTD13 PE=4 SV=1	0,36	0,08	77,80	74321631,3	15	1	0	1,52	329	2883
>tr|F1PR54|F1PR54_CANLF Lactotransferrin OS=Canis lupus familiaris OX=9615 GN=LTF PE=3 SV=1	42,10	5,92	628,10	71831632,5	51	16	2	28,53	708	40436
>tr|F1PJ71|F1PJ71_CANLF Glutathione peroxidase OS=Canis lupus familiaris OX=9615 GN=GPX5 PE=3 SV=2	9,70	3,55	419,20	64942776,3	10	3	0	16,74	221	19009
>tr|F1PJ71|F1PJ71_CANLF Glutathione peroxidase OS=Canis lupus familiaris OX=9615 GN=GPX5 PE=3 SV=2	12,57	5,34	557,60	60196690,9	17	4	0	23,53	221	19009

>sp|Q28895|NPC2_CANLF NPC intracellular cholesterol transporter 2 OS=Canis lupus familiaris OX=9615 GN=NPC2 PE=2 SV=1	15,24	9,78	742,20	60071338,4	18	3	0	30,87	149	153
>tr|F1PLT8|F1PLT8_CANLF Sulfhydryl oxidase OS=Canis lupus familiaris OX=9615 GN=QSOX1 PE=3 SV=3	3,42	3,32	251,40	59419835,3	5	2	0	5,46	568	33056
>tr|F1PR54|F1PR54_CANLF Lactotransferrin OS=Canis lupus familiaris OX=9615 GN=LTF PE=3 SV=1	40,69	8,49	660,60	55984690,4	58	13	1	22,46	708	40436
>tr|J9NVE0|J9NVE0_CANLF KRAB domain-containing protein OS=Canis lupus familiaris OX=9615 GN=LOC606925 PE=4 SV=1	0,20	0,10	122,60	55238541,0	6	1	0	4,08	147	3752
>tr|A0A5F4D454|A0A5F4D454_CANLF Phosphoinositide-3-kinase regulatory subunit 4 OS=Canis lupus familiaris OX=9615 GN=PIK3R4 PE=4 SV=1	0,44	0,42	184,50	54900368,0	2	1	0	0,99	1313	3150
>sp|O18840|ACTB_CANLF Actin, cytoplasmic 1 OS=Canis lupus familiaris OX=9615 GN=ACTB PE=2 SV=3	8,21	4,14	431,00	53549872,4	15	4	0	14,13	375	642
>tr|F1PR54|F1PR54_CANLF Lactotransferrin OS=Canis lupus familiaris OX=9615 GN=LTF PE=3 SV=1	50,07	3,63	548,60	52934880,3	75	20	1	33,62	708	40436
>tr|A0A5F4DEZ2|A0A5F4DEZ2_CANLF DAB2 interacting protein OS=Canis lupus familiaris OX=9615 GN=DAB2IP PE=4 SV=1	0,10	0,06	203,30	51761766,5	2	1	0	0,79	1272	12373
>tr|F1PLV2|F1PLV2_CANLF Peptidyl-prolyl cis-trans isomerase OS=Canis lupus familiaris OX=9615 GN=CSNK1G1 PE=3 SV=3	3,57	3,53	293,00	51412001,1	3	1	0	5,35	243	4290
>tr|A0A5F4C9S3|A0A5F4C9S3_CANLF Boule homolog, RNA binding protein OS=Canis lupus familiaris OX=9615 GN=BOLL PE=4 SV=1	0,22	0,04	174,10	50393536,9	10	1	0	1,92	365	16536
>tr|E2REQ4|E2REQ4_CANLF NIPA like domain containing 3 OS=Canis lupus familiaris OX=9615 GN=NIPAL3 PE=3 SV=3	0,10	0,03	122,00	49581947,1	3	2	0	2,17	368	39451
>tr|F1PYU7|F1PYU7_CANLF Cyclin A1 OS=Canis lupus familiaris OX=9615 GN=CCNA1 PE=3 SV=2	0,10	0,02	153,20	47824851,1	3	1	0	2,38	421	9029
>sp|Q6AW47|EST5A_CANLF Carboxylesterase 5A OS=Canis lupus familiaris OX=9615 GN=CES5A PE=2 SV=1	4,34	4,16	439,70	47480101,6	10	1	0	3,13	575	629
>sp|Q28895|NPC2_CANLF NPC intracellular cholesterol transporter 2 OS=Canis lupus familiaris OX=9615 GN=NPC2 PE=2 SV=1	15,31	9,10	726,30	47099576,9	22	2	0	24,16	149	153
>sp|Q9GL25|ESPB1_CANLF Epididymal sperm-binding protein 1 OS=Canis lupus familiaris OX=9615 GN=ELSPBP1 PE=1 SV=1	4,74	3,48	315,60	45398380,2	11	3	1	10,20	245	36
>tr|A0A5F4CKD5|A0A5F4CKD5_CANLF Polypeptide N-acetylgalactosaminyltransferase OS=Canis lupus familiaris OX=9615 GN=GALNT6 PE=3 SV=1	5,01	3,13	322,00	45307211,3	3	2	0	7,30	644	1617
>sp|O46607|GPX5_CANLF Epididymal secretory glutathione peroxidase OS=Canis lupus familiaris OX=9615 GN=GPX5 PE=2 SV=1	16,83	9,04	740,40	45137405,8	21	5	1	33,94	221	564
>tr|J9NS29|J9NS29_CANLF Cystatin domain-containing protein OS=Canis lupus familiaris OX=9615 GN=LOC607874 PE=4 SV=2	4,13	4,07	348,80	44905501,6	4	1	0	4,79	313	30016
>tr|J9P748|J9P748_CANLF Bromodomain adjacent to zinc finger domain 1A OS=Canis lupus familiaris OX=9615 GN=BAZ1A PE=4 SV=1	1,23	1,22	299,60	44798397,9	2	1	0	0,45	1557	10939
>sp|Q28895|NPC2_CANLF NPC intracellular cholesterol transporter 2 OS=Canis lupus familiaris OX=9615 GN=NPC2 PE=2 SV=1	10,69	10,47	787,80	41745352,9	12	1	0	15,44	149	153
>sp|P49822|ALBU_CANLF Albumin OS=Canis lupus familiaris OX=9615 GN=ALB PE=1 SV=3	3,83	1,86	362,70	39324167,1	8	4	2	7,24	608	490
>sp|Q9GL25|ESPB1_CANLF Epididymal sperm-binding protein 1 OS=Canis lupus familiaris OX=9615 GN=ELSPBP1 PE=1 SV=1	5,69	4,25	285,60	37587280,5	10	3	1	10,20	245	36

>tr|J9P432|J9P432_CANLF Glutamine--fructose-6-phosphate transaminase (isomerizing) OS=Canis lupus familiaris OX=9615 GN=GFPT1 PE=4 SV=2	0,14	0,10	149,50	35083407,2	3	1	0	1,18	677	7191
>tr|A0A5F4DGF5|A0A5F4DGF5_CANLF Alkaline phosphatase OS=Canis lupus familiaris OX=9615 GN=ALPL PE=3 SV=1	2,45	2,41	249,70	34518166,7	3	1	0	1,92	572	6357
>tr|J9P1P6|J9P1P6_CANLF Coiled-coil domain-containing protein 25 OS=Canis lupus familiaris OX=9615 PE=3 SV=2	1,03	0,81	99,30	33600395,0	12	1	0	1,71	292	12368
>sp|Q28895|NPC2_CANLF NPC intracellular cholesterol transporter 2 OS=Canis lupus familiaris OX=9615 GN=NPC2 PE=2 SV=1	8,20	8,06	687,00	33063508,8	8	1	0	15,44	149	153
>tr|J9NWY1|J9NWY1_CANLF Quinoid dihydropteridine reductase OS=Canis lupus familiaris OX=9615 GN=QDPR PE=3 SV=2	3,16	3,04	311,70	32809086,6	7	1	0	6,42	296	22710
>tr|J9NVE0|J9NVE0_CANLF KRAB domain-containing protein OS=Canis lupus familiaris OX=9615 GN=LOC606925 PE=4 SV=1	0,12	0,04	112,60	32654726,5	6	1	0	4,08	147	3752
>sp|Q9XS65|PTGDS_CANLF Prostaglandin-H2 D-isomerase OS=Canis lupus familiaris OX=9615 GN=PTGDS PE=2 SV=1	5,51	4,00	428,50	32515314,1	9	2	0	32,46	191	165
>sp|Q9XS65|PTGDS_CANLF Prostaglandin-H2 D-isomerase OS=Canis lupus familiaris OX=9615 GN=PTGDS PE=2 SV=1	7,67	5,93	568,10	32510605,5	14	3	1	32,46	191	165
>tr|E2RNH2|E2RNH2_CANLF Ras protein specific guanine nucleotide releasing factor 1 OS=Canis lupus familiaris OX=9615 GN=RASGRF1 PE=4 SV=2	0,11	0,03	102,20	31642930,1	5	1	0	0,39	1287	9564
>sp|P25473|CLUS_CANLF Clusterin OS=Canis lupus familiaris OX=9615 GN=CLU PE=2 SV=1	3,10	1,88	248,20	31230418,7	8	3	0	7,64	445	725
>tr|A0A5F4D4B6|A0A5F4D4B6_CANLF Immunoglobulin superfamily member 8 OS=Canis lupus familiaris OX=9615 GN=IGSF8 PE=4 SV=1	0,23	0,19	139,00	30867342,4	3	1	0	1,99	905	16467
>sp|Q9XS65|PTGDS_CANLF Prostaglandin-H2 D-isomerase OS=Canis lupus familiaris OX=9615 GN=PTGDS PE=2 SV=1	7,32	3,79	511,90	30149701,9	8	2	1	16,23	191	165
>tr|A0A5F4C9S3|A0A5F4C9S3_CANLF Boule homolog, RNA binding protein OS=Canis lupus familiaris OX=9615 GN=BOLL PE=4 SV=1	0,10	0,05	156,00	30149248,7	3	1	0	1,92	365	16536
>tr|E2REQ4|E2REQ4_CANLF NIPA like domain containing 3 OS=Canis lupus familiaris OX=9615 GN=NIPAL3 PE=3 SV=3	0,11	0,03	137,80	30108504,1	5	2	0	2,17	368	39451
>tr|E2R838|E2R838_CANLF Intraflagellar transport 88 OS=Canis lupus familiaris OX=9615 GN=IFT88 PE=4 SV=2	0,10	0,05	115,00	29961826,5	3	1	0	0,73	825	7901
>tr|A0A5F4D4B6|A0A5F4D4B6_CANLF Immunoglobulin superfamily member 8 OS=Canis lupus familiaris OX=9615 GN=IGSF8 PE=4 SV=1	0,11	0,09	168,10	29468807,3	2	1	0	1,99	905	16467
>tr|A0A5F4C9S3|A0A5F4C9S3_CANLF Boule homolog, RNA binding protein OS=Canis lupus familiaris OX=9615 GN=BOLL PE=4 SV=1	0,11	0,02	184,70	29413623,8	9	1	0	1,92	365	16536
>tr|A0A5F4C4Q6|A0A5F4C4Q6_CANLF Arylsulfatase B OS=Canis lupus familiaris OX=9615 GN=ARSB PE=3 SV=1	0,10	0,08	109,00	28541326,5	2	1	0	1,46	411	38230
>tr|J9P1P6|J9P1P6_CANLF Coiled-coil domain-containing protein 25 OS=Canis lupus familiaris OX=9615 PE=3 SV=2	1,03	0,79	95,20	26899095,1	13	1	0	1,71	292	12368
>tr|F1PIZ1|F1PIZ1_CANLF Caspase recruitment domain family member 6 OS=Canis lupus familiaris OX=9615 GN=CARD6 PE=4 SV=3	1,50	1,26	117,10	26635879,0	13	1	0	0,65	1071	14260
>tr|J9NVE0|J9NVE0_CANLF KRAB domain-containing protein OS=Canis lupus familiaris OX=9615 GN=LOC606925 PE=4 SV=1	0,11	0,05	124,50	26464712,7	4	1	0	4,08	147	3752
>tr|A0A5F4C3M5|A0A5F4C3M5_CANLF IQ motif containing GTPase activating protein 2 OS=Canis lupus familiaris OX=9615 GN=IQGAP2 PE=4 SV=1	0,10	0,03	78,20	26309008,9	3	1	0	0,51	1577	1056
>tr|F2Z4Q6|F2Z4Q6_CANLF Alpha fetoprotein OS=Canis lupus familiaris	29,44	9,90	801,40	26270075,3	33	7	0	18,05	637	24990

OX=9615 GN=AFP PE=4 SV=2										
>sp|P31637|SC5A3_CANLF Sodium/myo-inositol cotransporter OS=Canis lupus familiaris OX=9615 GN=SLC5A3 PE=2 SV=1	0,21	0,21	202,40	25526495,7	1	1	0	0,56	718	62
>tr|A0A5F4CCD0|A0A5F4CCD0_CANLF Cysteine rich secretory protein 2 OS=Canis lupus familiaris OX=9615 GN=CRISP2 PE=3 SV=1	7,15	2,96	352,60	24822205,8	6	3	1	15,76	311	11017
>tr|E2RCT1|E2RCT1_CANLF WAP domain-containing protein OS=Canis lupus familiaris OX=9615 PE=4 SV=2	6,36	6,26	649,80	24493652,4	6	1	0	13,79	116	21717
>tr|E2RH40|E2RH40_CANLF Cilia and flagella associated protein 69 OS=Canis lupus familiaris OX=9615 GN=CFAP69 PE=4 SV=2	0,42	0,37	56,00	24040685,6	4	1	0	0,53	941	3287
>sp|O18835|BGLR_CANLF Beta-glucuronidase OS=Canis lupus familiaris OX=9615 GN=GUSB PE=2 SV=1	0,29	0,27	204,80	23964494,5	2	1	0	4,30	651	699
>tr|E2RIN3|E2RIN3_CANLF Otoancorin OS=Canis lupus familiaris OX=9615 GN=OTOA PE=3 SV=3	0,10	0,03	89,00	23858117,0	3	1	0	0,70	1139	18241
>tr|J9P1P6|J9P1P6_CANLF Coiled-coil domain-containing protein 25 OS=Canis lupus familiaris OX=9615 PE=3 SV=2	1,14	0,98	101,60	23841076,9	9	1	0	1,71	292	12368
>sp|Q9XS65|PTGDS_CANLF Prostaglandin-H2 D-isomerase OS=Canis lupus familiaris OX=9615 GN=PTGDS PE=2 SV=1	3,89	3,39	343,50	23787909,2	4	2	1	16,23	191	165
>tr|F2Z4Q6|F2Z4Q6_CANLF Alpha fetoprotein OS=Canis lupus familiaris OX=9615 GN=AFP PE=4 SV=2	21,03	3,89	501,70	23429036,8	16	6	0	16,17	637	24990
>tr|A0A5F4C6B5|A0A5F4C6B5_CANLF Plastin 3 OS=Canis lupus familiaris OX=9615 GN=PLS3 PE=4 SV=1	6,46	2,75	339,50	23154831,8	7	3	0	8,14	639	30730
>sp|E2RK33|GATC_CANLF Glutamyl-tRNA(Gln) amidotransferase subunit C, mitochondrial OS=Canis lupus familiaris OX=9615 GN=GATC PE=3 SV=1	1,16	1,16	166,80	22564281,2	1	1	0	2,58	155	39
>sp|Q9XS65|PTGDS_CANLF Prostaglandin-H2 D-isomerase OS=Canis lupus familiaris OX=9615 GN=PTGDS PE=2 SV=1	10,72	3,79	402,50	22378291,7	24	5	3	47,12	191	165
>tr|E2RSI6|E2RSI6_CANLF Ezrin OS=Canis lupus familiaris OX=9615 GN=EZR PE=4 SV=1	0,32	0,30	219,60	22156133,5	2	1	0	2,90	586	15650
>tr|A0A5F4C9S3|A0A5F4C9S3_CANLF Boule homolog, RNA binding protein OS=Canis lupus familiaris OX=9615 GN=BOLL PE=4 SV=1	0,16	0,06	158,60	21941903,4	6	1	0	1,92	365	16536
>tr|A0A5F4D8K0|A0A5F4D8K0_CANLF Phosphatidylinositol-4-phosphate 3-kinase OS=Canis lupus familiaris OX=9615 GN=PIK3C2B PE=3 SV=1	0,12	0,12	108,80	21744786,9	1	1	1	1,00	1606	24952
>sp|Q28895|NPC2_CANLF NPC intracellular cholesterol transporter 2 OS=Canis lupus familiaris OX=9615 GN=NPC2 PE=2 SV=1	13,11	7,67	682,40	20811083,0	8	2	0	24,16	149	153
>tr|F1Q1M8|F1Q1M8_CANLF Beta-N-acetylhexosaminidase OS=Canis lupus familiaris OX=9615 GN=HEXB PE=3 SV=3	2,10	2,06	316,90	20521807,8	3	1	0	6,36	330	5947
>sp|Q28895|NPC2_CANLF NPC intracellular cholesterol transporter 2 OS=Canis lupus familiaris OX=9615 GN=NPC2 PE=2 SV=1	5,84	5,78	566,80	19916920,7	4	1	0	15,44	149	153
>tr|E2R0D9|E2R0D9_CANLF Solute carrier family 17 member 2 OS=Canis lupus familiaris OX=9615 GN=SLC17A2 PE=4 SV=1	0,20	0,18	200,40	19844907,0	2	1	0	2,51	478	30466
>tr|F1P9H6|F1P9H6_CANLF Chloride intracellular channel protein OS=Canis lupus familiaris OX=9615 GN=CLIC5 PE=3 SV=3	1,68	1,68	249,10	19627818,2	1	1	0	2,78	252	14180
>sp|Q6AW47|EST5A_CANLF Carboxylesterase 5A OS=Canis lupus familiaris OX=9615 GN=CES5A PE=2 SV=1	4,13	3,78	418,70	19021538,1	6	2	0	6,09	575	629
>tr|F1PLV2|F1PLV2_CANLF Peptidyl-prolyl cis-trans isomerase OS=Canis lupus familiaris OX=9615 GN=CSNK1G1 PE=3 SV=3	2,93	2,91	289,70	18955680,8	2	1	0	5,35	243	4290

>tr|F1PKE7|F1PKE7_CANLF C-type lectin domain containing 16A OS=Canis lupus familiaris OX=9615 GN=CLEC16A PE=3 SV=3	0,16	0,16	158,10	17816551,0	1	1	0	1,52	990	2034
>sp|Q9XS65|PTGDS_CANLF Prostaglandin-H2 D-isomerase OS=Canis lupus familiaris OX=9615 GN=PTGDS PE=2 SV=1	5,30	3,85	416,20	17285021,9	10	2	1	32,46	191	165
>sp|P49822|ALBU_CANLF Albumin OS=Canis lupus familiaris OX=9615 GN=ALB PE=1 SV=3	13,88	3,18	485,90	17165785,2	22	5	0	13,16	608	490
>tr|A0A5F4DGF5|A0A5F4DGF5_CANLF Alkaline phosphatase OS=Canis lupus familiaris OX=9615 GN=ALPL PE=3 SV=1	3,99	3,89	453,90	16779524,8	6	1	0	1,92	572	6357
>tr|A0A5F4DGF5|A0A5F4DGF5_CANLF Alkaline phosphatase OS=Canis lupus familiaris OX=9615 GN=ALPL PE=3 SV=1	1,17	1,13	239,00	16764800,4	3	1	0	1,92	572	6357
>tr|F1PSC2|F1PSC2_CANLF Talin 1 OS=Canis lupus familiaris OX=9615 GN=TLN1 PE=4 SV=3	0,54	0,54	139,90	16574271,5	1	1	0	0,63	2558	6161
>tr|A0A5F4CDM1|A0A5F4CDM1_CANLF L-lactate dehydrogenase OS=Canis lupus familiaris OX=9615 PE=3 SV=1	0,10	0,07	113,30	16475929,5	2	1	0	2,87	279	43073
>tr|J9P3D0|J9P3D0_CANLF Solute carrier family 4 member 9 OS=Canis lupus familiaris OX=9615 GN=SLC4A9 PE=3 SV=2	0,10	0,07	147,80	16474014,5	1	1	0	1,12	893	31921
>tr|J9P432|J9P432_CANLF Glutamine--fructose-6-phosphate transaminase (isomerizing) OS=Canis lupus familiaris OX=9615 GN=GFPT1 PE=4 SV=2	0,37	0,29	182,50	15658742,1	5	1	0	1,18	677	7191
>tr|J9NVE0|J9NVE0_CANLF KRAB domain-containing protein OS=Canis lupus familiaris OX=9615 GN=LOC606925 PE=4 SV=1	0,54	0,48	105,70	15564671,2	4	1	0	4,08	147	3752
>tr|A0A5F4CQU2|A0A5F4CQU2_CANLF Ig-like domain-containing protein OS=Canis lupus familiaris OX=9615 GN=DLA-DMB PE=3 SV=1	0,10	0,10	112,20	15539007,3	1	1	0	4,10	244	18966
>tr|A0A5F4CI02|A0A5F4CI02_CANLF Solute carrier family 35 member F4 OS=Canis lupus familiaris OX=9615 GN=SLC35F4 PE=3 SV=1	0,12	0,10	151,90	15322390,1	2	1	0	2,29	480	7991
>tr|J9NWY1|J9NWY1_CANLF Quinoid dihydropteridine reductase OS=Canis lupus familiaris OX=9615 GN=QDPR PE=3 SV=2	1,25	1,19	232,70	15292373,8	4	1	0	6,42	296	22710
>tr|E2QY78|E2QY78_CANLF BRCA1 associated ATM activator 1 OS=Canis lupus familiaris OX=9615 GN=BRAT1 PE=4 SV=1	0,41	0,42	189,40	14634704,2	1	1	1	2,31	824	7928
>sp|Q9GL25|ESPB1_CANLF Epididymal sperm-binding protein 1 OS=Canis lupus familiaris OX=9615 GN=ELSPBP1 PE=1 SV=1	3,78	1,41	287,00	14553556,4	10	3	1	12,65	245	36
>tr|F1PEZ5|F1PEZ5_CANLF Laminin subunit alpha 2 OS=Canis lupus familiaris OX=9615 GN=LAMA2 PE=4 SV=3	0,70	0,62	78,70	14359506,4	5	1	0	0,51	3112	8576
>sp|Q9XS65|PTGDS_CANLF Prostaglandin-H2 D-isomerase OS=Canis lupus familiaris OX=9615 GN=PTGDS PE=2 SV=1	1,10	0,72	242,20	13819713,3	6	3	1	32,46	191	165
>tr|A0A5F4CCD0|A0A5F4CCD0_CANLF Cysteine rich secretory protein 2 OS=Canis lupus familiaris OX=9615 GN=CRISP2 PE=3 SV=1	4,55	4,06	356,50	13780661,0	8	2	1	10,29	311	11017
>tr|E2RSI6|E2RSI6_CANLF Ezrin OS=Canis lupus familiaris OX=9615 GN=EZR PE=4 SV=1	2,68	2,64	307,50	13754811,4	3	1	0	2,90	586	15650
>tr|F1PB68|F1PB68_CANLF Olfactomedin 4 OS=Canis lupus familiaris OX=9615 GN=OLFM4 PE=4 SV=3	7,39	3,93	405,80	13649912,6	5	3	0	9,64	477	17246
>sp|Q9XS65|PTGDS_CANLF Prostaglandin-H2 D-isomerase OS=Canis lupus familiaris OX=9615 GN=PTGDS PE=2 SV=1	5,25	4,02	544,60	13397958,5	10	2	1	16,23	191	165
>tr|F6Y258|F6Y258_CANLF General transcription factor IIi OS=Canis lupus familiaris OX=9615 GN=GTF2I PE=4 SV=1	0,10	0,07	91,40	13390505,4	2	1	0	0,70	999	5437
>tr|E2RCT1|E2RCT1_CANLF WAP domain-containing protein	5,46	5,35	574,60	13072308,9	5	2	1	13,79	116	21717

OS=Canis lupus familiaris OX=9615 PE=4 SV=2										
>tr|J9P3D0|J9P3D0_CANLF Solute carrier family 4 member 9 OS=Canis lupus familiaris OX=9615 GN=SLC4A9 PE=3 SV=2	0,48	0,44	181,80	12726751,4	3	1	0	1,12	893	31921
>tr|E2RNH2|E2RNH2_CANLF Ras protein specific guanine nucleotide releasing factor 1 OS=Canis lupus familiaris OX=9615 GN=RASGRF1 PE=4 SV=2	0,47	0,45	100,30	12402737,6	2	1	0	0,39	1287	9564
>tr|J9NS29|J9NS29_CANLF Cystatin domain-containing protein OS=Canis lupus familiaris OX=9615 GN=LOC607874 PE=4 SV=2	4,26	4,20	522,20	11828286,0	4	1	0	4,79	313	30016
>tr|A0A5F4C6B5|A0A5F4C6B5_CANLF Plastin 3 OS=Canis lupus familiaris OX=9615 GN=PLS3 PE=4 SV=1	4,01	2,06	389,30	11704135,3	6	2	0	9,39	639	30730
>tr|F1Q1M8|F1Q1M8_CANLF Beta-N-acetylhexosaminidase OS=Canis lupus familiaris OX=9615 GN=HEXB PE=3 SV=3	1,80	1,76	323,30	11621449,5	3	1	0	8,18	330	5947
>tr|A0A5F4CCD0|A0A5F4CCD0_CANLF Cysteine rich secretory protein 2 OS=Canis lupus familiaris OX=9615 GN=CRISP2 PE=3 SV=1	5,96	4,58	537,50	11415435,2	6	2	1	16,40	311	11017
>tr|A0A5F4CQU2|A0A5F4CQU2_CANLF Ig-like domain-containing protein OS=Canis lupus familiaris OX=9615 GN=DLA-DMB PE=3 SV=1	0,48	0,48	137,20	11331722,5	1	1	0	4,10	244	18966
>tr|A0A5F4CYM0|A0A5F4CYM0_CANLF Quiescin sulfhydryl oxidase 2 OS=Canis lupus familiaris OX=9615 GN=QSOX2 PE=4 SV=1	1,26	1,26	169,10	11093475,4	1	1	0	1,42	636	1464
>tr|J9P1P6|J9P1P6_CANLF Coiled-coil domain-containing protein 25 OS=Canis lupus familiaris OX=9615 PE=3 SV=2	1,21	1,11	56,50	10792952,3	6	1	0	1,71	292	12368
>tr|J9P432|J9P432_CANLF Glutamine--fructose-6-phosphate transaminase (isomerizing) OS=Canis lupus familiaris OX=9615 GN=GFPT1 PE=4 SV=2	0,37	0,29	190,70	10775970,7	5	1	0	1,18	677	7191
>tr|A0A5F4CKD5|A0A5F4CKD5_CANLF Polypeptide N-acetylgalactosaminyltransferase OS=Canis lupus familiaris OX=9615 GN=GALNT6 PE=3 SV=1	2,53	2,49	436,80	10736169,6	3	1	0	4,97	644	1617
>tr|A0A5F4CCD0|A0A5F4CCD0_CANLF Cysteine rich secretory protein 2 OS=Canis lupus familiaris OX=9615 GN=CRISP2 PE=3 SV=1	5,98	3,04	484,40	10434750,8	5	2	1	10,29	311	11017
>tr|F6X9G7|F6X9G7_CANLF Lipocalin 9 OS=Canis lupus familiaris OX=9615 GN=LCN9 PE=3 SV=2	1,69	1,69	201,90	10361421,3	1	1	0	6,85	219	14681
>tr|F1Q1M8|F1Q1M8_CANLF Beta-N-acetylhexosaminidase OS=Canis lupus familiaris OX=9615 GN=HEXB PE=3 SV=3	3,19	3,17	269,90	10278207,1	2	1	0	6,36	330	5947
>tr|E2R6E0|E2R6E0_CANLF Lipocln_cytosolic_FA-bd_dom domain-containing protein OS=Canis lupus familiaris OX=9615 GN=LCNL1 PE=3 SV=2	3,56	3,54	262,40	10246112,3	2	1	0	3,01	299	1932
>tr|A0A5F4DBM1|A0A5F4DBM1_CANLF Lipocln_cytosolic_FA-bd_dom domain-containing protein OS=Canis lupus familiaris OX=9615 GN=LCN6 PE=3 SV=1	4,70	3,50	383,20	9876472,7	4	2	1	14,41	236	4304
>tr|A0A5F4C6B5|A0A5F4C6B5_CANLF Plastin 3 OS=Canis lupus familiaris OX=9615 GN=PLS3 PE=4 SV=1	4,89	2,08	373,70	9821997,6	5	3	0	9,39	639	30730
>tr|F1PJ71|F1PJ71_CANLF Glutathione peroxidase OS=Canis lupus familiaris OX=9615 GN=GPX5 PE=3 SV=2	11,20	3,77	455,70	9792671,4	14	5	0	26,24	221	19009
>tr|A0A5F4C1D8|A0A5F4C1D8_CANLF Acrosin OS=Canis lupus familiaris OX=9615 GN=ACR PE=3 SV=1	1,06	1,06	182,30	9785536,5	1	1	0	2,13	423	43346
>tr|E2RJF6|E2RJF6_CANLF Mortality factor 4 like 1 OS=Canis lupus familiaris OX=9615 GN=MORF4L1 PE=4 SV=3	0,17	0,17	95,40	9416311,9	1	1	0	4,56	439	18584
>tr|A0A5F4CQU2|A0A5F4CQU2_CANLF Ig-like domain-containing protein OS=Canis lupus familiaris OX=9615 GN=DLA-DMB PE=3 SV=1	0,89	0,87	107,60	9342887,3	2	1	0	4,10	244	18966

>tr|E2RSI6|E2RSI6_CANLF Ezrin OS=Canis lupus familiaris OX=9615 GN=EZR PE=4 SV=1	1,12	1,10	236,50	9024482,8	2	1	0	1,54	586	15650
>tr|A0A5F4C6L3|A0A5F4C6L3_CANLF Cyclic nucleotide binding domain containing 1 OS=Canis lupus familiaris OX=9615 GN=CNBD1 PE=4 SV=1	0,50	0,48	84,40	8524384,1	2	1	0	1,07	563	8180
>tr|F2Z4Q6|F2Z4Q6_CANLF Alpha fetoprotein OS=Canis lupus familiaris OX=9615 GN=AFP PE=4 SV=2	7,72	2,87	404,90	8442472,4	19	5	0	12,56	637	24990
>tr|F1PJ71|F1PJ71_CANLF Glutathione peroxidase OS=Canis lupus familiaris OX=9615 GN=GPX5 PE=3 SV=2	4,77	3,60	467,30	8437068,9	5	2	0	10,41	221	19009
>tr|F1PHH0|F1PHH0_CANLF Neurotrimin OS=Canis lupus familiaris OX=9615 GN=NTM PE=4 SV=3	0,13	0,13	259,80	8302105,3	1	1	0	6,67	345	11396
>tr|A0A5F4D7L4|A0A5F4D7L4_CANLF Metalloproteinase inhibitor 1 OS=Canis lupus familiaris OX=9615 GN=TIMP1 PE=4 SV=1	0,29	0,29	269,00	7854050,9	1	1	1	2,79	359	13115
>tr|F1PB68|F1PB68_CANLF Olfactomedin 4 OS=Canis lupus familiaris OX=9615 GN=OLFM4 PE=4 SV=3	3,95	3,93	350,80	7620326,5	2	1	0	4,40	477	17246
>tr|J9PA86|J9PA86_CANLF Zinc finger protein 630 OS=Canis lupus familiaris OX=9615 GN=ZNF630 PE=4 SV=1	0,50	0,48	84,70	7598137,1	2	1	0	0,89	675	839
>tr|A0A5F4C9S3|A0A5F4C9S3_CANLF Boule homolog, RNA binding protein OS=Canis lupus familiaris OX=9615 GN=BOLL PE=4 SV=1	0,29	0,24	198,10	7575300,9	4	1	0	1,92	365	16536
>sp|B6V8E6|CTNB1_CANLF Catenin beta-1 OS=Canis lupus familiaris OX=9615 GN=CTNNB1 PE=1 SV=1	1,08	1,06	175,70	7563280,8	2	1	0	0,51	781	442
>tr|A0A5F4C3V3|A0A5F4C3V3_CANLF Olfactory receptor OS=Canis lupus familiaris OX=9615 GN=OR4C11I PE=3 SV=1	0,20	0,16	139,30	7471399,7	3	1	0	4,81	312	9939
>sp|Q28895|NPC2_CANLF NPC intracellular cholesterol transporter 2 OS=Canis lupus familiaris OX=9615 GN=NPC2 PE=2 SV=1	8,91	4,30	542,70	7356904,9	11	3	0	30,20	149	153
>tr|A0A5F4C6L3|A0A5F4C6L3_CANLF Cyclic nucleotide binding domain containing 1 OS=Canis lupus familiaris OX=9615 GN=CNBD1 PE=4 SV=1	0,27	0,20	78,80	7316858,6	4	2	1	2,13	563	8180
>tr|E2R5K8|E2R5K8_CANLF G protein pathway suppressor 2 OS=Canis lupus familiaris OX=9615 GN=GPS2 PE=4 SV=3	0,14	0,14	133,30	7283578,6	1	1	0	3,98	327	32341
>tr|A0A5F4CXB9|A0A5F4CXB9_CANLF Testis expressed 9 OS=Canis lupus familiaris OX=9615 GN=TEX9 PE=4 SV=1	0,10	0,06	201,50	7191022,8	3	1	0	1,89	424	41102
>tr|A0A5F4CKD5|A0A5F4CKD5_CANLF Polypeptide N-acetylgalactosaminyltransferase OS=Canis lupus familiaris OX=9615 GN=GALNT6 PE=3 SV=1	9,23	9,15	745,00	6980515,3	5	1	0	4,97	644	1617
>tr|A0A5F4C6B5|A0A5F4C6B5_CANLF Plastin 3 OS=Canis lupus familiaris OX=9615 GN=PLS3 PE=4 SV=1	1,21	1,02	279,00	6682088,4	4	2	0	9,39	639	30730
>sp|O46607|GPX5_CANLF Epididymal secretory glutathione peroxidase OS=Canis lupus familiaris OX=9615 GN=GPX5 PE=2 SV=1	2,06	2,01	383,10	6592133,0	4	1	0	6,79	221	564
>sp|Q6AW47|EST5A_CANLF Carboxylesterase 5A OS=Canis lupus familiaris OX=9615 GN=CES5A PE=2 SV=1	2,14	2,12	263,40	6326996,9	2	1	0	3,13	575	629
>tr|A0A5F4C9S3|A0A5F4C9S3_CANLF Boule homolog, RNA binding protein OS=Canis lupus familiaris OX=9615 GN=BOLL PE=4 SV=1	0,90	0,82	172,80	6282147,0	5	1	0	1,92	365	16536
>sp|P25473|CLUS_CANLF Clusterin OS=Canis lupus familiaris OX=9615 GN=CLU PE=2 SV=1	9,03	3,48	339,30	5882980,3	12	4	0	9,89	445	725
>tr|A0A5F4C6B5|A0A5F4C6B5_CANLF Plastin 3 OS=Canis lupus familiaris OX=9615 GN=PLS3 PE=4 SV=1	3,10	2,01	257,60	5686347,3	3	2	0	6,26	639	30730

>tr|F1Q4G5|F1Q4G5_CANLF Helt bHLH transcription factor OS=Canis lupus familiaris OX=9615 GN=HELT PE=4 SV=2	0,14	0,14	192,10	5540898,4	1	1	0	4,76	231	31936
>tr|A0A5F4C1S8|A0A5F4C1S8_CANLF E3 ubiquitin-protein ligase CBL OS=Canis lupus familiaris OX=9615 GN=CBL PE=4 SV=1	0,14	0,14	162,50	5540752,4	1	1	0	0,52	773	1308
>tr|F1PB83|F1PB83_CANLF Integrin subunit alpha 9 OS=Canis lupus familiaris OX=9615 GN=ITGA9 PE=3 SV=3	0,60	0,58	176,60	5423474,6	2	1	0	1,03	975	39570
>tr|A0A5F4CQU2|A0A5F4CQU2_CANLF Ig-like domain-containing protein OS=Canis lupus familiaris OX=9615 GN=DLA-DMB PE=3 SV=1	0,90	0,90	108,10	5406225,0	1	1	0	4,10	244	18966
>tr|A0A5F4C6L3|A0A5F4C6L3_CANLF Cyclic nucleotide binding domain containing 1 OS=Canis lupus familiaris OX=9615 GN=CNBD1 PE=4 SV=1	0,12	0,10	89,00	5359118,1	2	1	0	1,07	563	8180
>tr|A0A5F4C3M5|A0A5F4C3M5_CANLF IQ motif containing GTPase activating protein 2 OS=Canis lupus familiaris OX=9615 GN=IQGAP2 PE=4 SV=1	1,21	1,13	134,90	5297370,8	5	1	0	0,51	1577	1056
>sp|Q9GL25|ESPB1_CANLF Epididymal sperm-binding protein 1 OS=Canis lupus familiaris OX=9615 GN=ELSPBP1 PE=1 SV=1	6,14	3,40	413,10	5162129,7	6	2	0	16,33	245	36
>tr|A0A5F4CHN1|A0A5F4CHN1_CANLF Serine/arginine repetitive matrix 2 OS=Canis lupus familiaris OX=9615 GN=SRRM2 PE=4 SV=1	0,14	0,10	126,30	5029683,4	2	2	0	0,47	2564	3180
>sp|O18840|ACTB_CANLF Actin, cytoplasmic 1 OS=Canis lupus familiaris OX=9615 GN=ACTB PE=2 SV=3	9,51	3,75	355,60	4914556,3	8	5	1	22,13	375	642
>sp|P25473|CLUS_CANLF Clusterin OS=Canis lupus familiaris OX=9615 GN=CLU PE=2 SV=1	1,35	1,06	212,40	4870772,8	2	2	1	8,54	445	725
>tr|E2RAK5|E2RAK5_CANLF Sulfotransferase OS=Canis lupus familiaris OX=9615 GN=SULT1C3 PE=3 SV=1	0,14	0,12	139,20	4789099,3	2	1	0	2,30	304	12938
>sp|O46607|GPX5_CANLF Epididymal secretory glutathione peroxidase OS=Canis lupus familiaris OX=9615 GN=GPX5 PE=2 SV=1	2,75	2,68	445,60	4782732,3	5	1	0	6,79	221	564
>tr|Q5TJG5|Q5TJG5_CANLF MHC class II antigen DO alpha OS=Canis lupus familiaris OX=9615 GN=DLA-DOA PE=3 SV=1	0,75	0,73	136,00	4692040,7	2	1	0	4,00	250	41318
>tr|J9NVE0|J9NVE0_CANLF KRAB domain-containing protein OS=Canis lupus familiaris OX=9615 GN=LOC606925 PE=4 SV=1	0,20	0,18	88,00	4692040,7	2	1	0	4,08	147	3752
>tr|F1Q107|F1Q107_CANLF Multiple C2 and transmembrane domain containing 1 OS=Canis lupus familiaris OX=9615 GN=MCTP1 PE=4 SV=3	0,10	0,07	112,80	4686595,4	1	1	0	1,06	752	28373
>tr|J9P432|J9P432_CANLF Glutamine--fructose-6-phosphate transaminase (isomerizing) OS=Canis lupus familiaris OX=9615 GN=GFPT1 PE=4 SV=2	0,11	0,09	170,30	4669041,6	2	1	0	1,18	677	7191
>tr|E2R6E0|E2R6E0_CANLF Lipocln_cytosolic_FA-bd_dom domain-containing protein OS=Canis lupus familiaris OX=9615 GN=LCNL1 PE=3 SV=2	2,80	2,68	295,10	4653961,3	7	1	0	3,68	299	1932
>tr|F2Z4Q6|F2Z4Q6_CANLF Alpha fetoprotein OS=Canis lupus familiaris OX=9615 GN=AFP PE=4 SV=2	6,36	2,92	381,70	4644236,7	8	4	0	9,26	637	24990
>tr|J9NS29|J9NS29_CANLF Cystatin domain-containing protein OS=Canis lupus familiaris OX=9615 GN=LOC607874 PE=4 SV=2	3,06	3,06	245,30	4623424,8	1	1	0	4,79	313	30016
>tr|J9NS29|J9NS29_CANLF Cystatin domain-containing protein OS=Canis lupus familiaris OX=9615 GN=LOC607874 PE=4 SV=2	1,50	1,48	234,90	4619058,7	2	1	0	4,79	313	30016
>tr|J9P9J4|J9P9J4_CANLF Aldehyde dehydrogenase 1 family member A1 OS=Canis lupus familiaris OX=9615 GN=ALDH1A1 PE=3 SV=1	6,43	3,25	426,50	4616697,4	3	3	0	7,44	484	13765
>tr|F2Z4Q6|F2Z4Q6_CANLF Alpha fetoprotein OS=Canis lupus familiaris OX=9615 GN=AFP PE=4 SV=2	2,12	2,10	284,30	4585107,4	2	1	0	2,04	637	24990
>tr|F6X5V2|F6X5V2_CANLF Dual oxidase maturation factor 2	0,18	0,17	181,30	4416978,8	2	1	1	7,81	320	40162

OS=Canis lupus familiaris OX=9615 GN=DUOXA2 PE=3 SV=1										
>sp|Q9GL25|ESPB1_CANLF Epididymal sperm-binding protein 1 OS=Canis lupus familiaris OX=9615 GN=ELSPBP1 PE=1 SV=1	4,71	2,46	241,90	4167228,6	3	3	0	19,59	245	36
>tr|E2R6E0|E2R6E0_CANLF Lipocln_cytosolic_FA-bd_dom domain-containing protein OS=Canis lupus familiaris OX=9615 GN=LCNL1 PE=3 SV=2	9,30	3,76	419,60	3895695,2	9	3	0	10,37	299	1932
>sp|Q6AW47|EST5A_CANLF Carboxylesterase 5A OS=Canis lupus familiaris OX=9615 GN=CES5A PE=2 SV=1	3,44	3,42	292,40	3841637,6	2	1	0	3,13	575	629
>tr|A0A5F4C6L3|A0A5F4C6L3_CANLF Cyclic nucleotide binding domain containing 1 OS=Canis lupus familiaris OX=9615 GN=CNBD1 PE=4 SV=1	0,16	0,12	77,30	3829222,7	3	1	0	1,07	563	8180
>tr|F1PQ67|F1PQ67_CANLF LARGE xylosyl- and glucuronyltransferase 2 OS=Canis lupus familiaris OX=9615 GN=LARGE2 PE=4 SV=3	0,18	0,18	138,60	3824495,2	1	1	0	2,08	721	39792
>tr|E2RQF9|E2RQF9_CANLF Minichromosome maintenance complex component 3 associated protein OS=Canis lupus familiaris OX=9615 GN=MCM3AP PE=4 SV=3	0,89	0,89	289,00	3705304,3	1	1	0	0,25	1980	24083
>tr|E2RG75|E2RG75_CANLF Inactive ribonuclease-like protein 9 OS=Canis lupus familiaris OX=9615 GN=RNASE9 PE=3 SV=2	2,42	2,42	241,70	3580465,0	1	1	0	10,10	198	41734
>sp|P49822|ALBU_CANLF Albumin OS=Canis lupus familiaris OX=9615 GN=ALB PE=1 SV=3	0,77	0,47	356,10	3532564,0	2	2	1	7,24	608	490
>tr|A0A5F4C2J2|A0A5F4C2J2_CANLF Alpha-mannosidase OS=Canis lupus familiaris OX=9615 GN=MAN2B1 PE=3 SV=1	1,37	1,36	204,00	3529999,1	2	1	0	2,21	1042	3130
>tr|E2RGF2|E2RGF2_CANLF BRCA1 associated protein 1 OS=Canis lupus familiaris OX=9615 GN=BAP1 PE=3 SV=2	0,76	0,72	265,60	3429037,4	3	1	1	7,62	446	25285
>tr|J9P758|J9P758_CANLF Sorcin OS=Canis lupus familiaris OX=9615 GN=SRI PE=4 SV=2	0,20	0,20	150,60	3393556,2	1	1	0	2,30	348	7242
>tr|J9NY16|J9NY16_CANLF Zinc finger protein 541 OS=Canis lupus familiaris OX=9615 GN=ZNF541 PE=4 SV=2	0,35	0,35	172,40	3342780,6	1	1	0	0,96	1357	25760
>tr|E2RKA1|E2RKA1_CANLF Tyrosine-protein kinase receptor OS=Canis lupus familiaris OX=9615 GN=NTRK2 PE=3 SV=2	0,27	0,24	94,50	3308700,2	4	1	0	0,97	822	20603
>tr|E2RHS5|E2RHS5_CANLF tRNA (guanine-N(7)-)-methyltransferase non-catalytic subunit WDR4 OS=Canis lupus familiaris OX=9615 GN=WDR4 PE=3 SV=1	0,11	0,09	56,80	3238526,3	2	1	0	1,72	406	22738
>tr|E2RQ68|E2RQ68_CANLF Ceramide synthase 2 OS=Canis lupus familiaris OX=9615 GN=CERS2 PE=4 SV=2	0,28	0,28	76,70	3214573,7	1	1	0	2,62	381	26313
>tr|A0A5F4CCD0|A0A5F4CCD0_CANLF Cysteine rich secretory protein 2 OS=Canis lupus familiaris OX=9615 GN=CRISP2 PE=3 SV=1	2,39	1,33	376,80	3192188,5	3	2	1	10,29	311	11017
>tr|A0A5F4D662|A0A5F4D662_CANLF Par-3 family cell polarity regulator beta OS=Canis lupus familiaris OX=9615 GN=PARD3B PE=3 SV=1	0,28	0,24	133,60	3190170,7	3	1	0	0,70	1142	8202
>tr|A0A5F4CPK4|A0A5F4CPK4_CANLF Formin binding protein 1 OS=Canis lupus familiaris OX=9615 GN=FNBP1 PE=4 SV=1	0,91	0,89	80,90	3159596,1	2	1	0	1,42	562	14071
>tr|F1PRL1|F1PRL1_CANLF Dppa2_A domain-containing protein OS=Canis lupus familiaris OX=9615 PE=4 SV=2	0,18	0,12	51,90	3152783,5	4	1	0	2,79	179	17222
>tr|F1PPY4|F1PPY4_CANLF Urokinase-type plasminogen activator OS=Canis lupus familiaris OX=9615 GN=PLAU PE=4 SV=2	0,32	0,32	145,70	3137645,3	1	1	0	3,31	543	42729
>sp|P49822|ALBU_CANLF Albumin OS=Canis lupus familiaris OX=9615 GN=ALB PE=1 SV=3	3,12	3,08	359,10	3127780,9	3	1	0	2,14	608	490
>tr|A0A5F4C9S3|A0A5F4C9S3_CANLF Boule homolog, RNA binding protein OS=Canis lupus familiaris OX=9615 GN=BOLL PE=4 SV=1	0,14	0,12	80,00	3112925,1	2	1	0	1,92	365	16536

>sp|Q6AW47|EST5A_CANLF Carboxylesterase 5A OS=Canis lupus familiaris OX=9615 GN=CES5A PE=2 SV=1	2,17	2,15	264,20	3043005,1	2	1	0	3,13	575	629
>tr|F1PB68|F1PB68_CANLF Olfactomedin 4 OS=Canis lupus familiaris OX=9615 GN=OLFM4 PE=4 SV=3	3,52	3,52	379,60	3020465,0	1	1	0	4,40	477	17246
>tr|F1PCA2|F1PCA2_CANLF Tripartite motif containing 27 OS=Canis lupus familiaris OX=9615 GN=TRIM27 PE=4 SV=3	0,96	0,96	207,60	2879057,6	1	1	0	4,98	462	7129
>tr|E2RQN2|E2RQN2_CANLF Peptidase S1 domain-containing protein OS=Canis lupus familiaris OX=9615 GN=PRSS58 PE=4 SV=1	1,68	1,66	400,60	2788005,1	2	1	0	10,79	241	44651
>tr|J9P434|J9P434_CANLF Myotubularin related protein 14 OS=Canis lupus familiaris OX=9615 GN=MTMR14 PE=4 SV=2	0,57	0,55	130,50	2742231,2	2	1	0	1,51	596	17413
>tr|E2R5P5|E2R5P5_CANLF Calcyclin OS=Canis lupus familiaris OX=9615 GN=S100A8 PE=4 SV=2	2,44	2,38	506,20	2695848,2	4	1	0	14,66	191	27843
>tr|E2R6E0|E2R6E0_CANLF Lipocln_cytosolic_FA-bd_dom domain-containing protein OS=Canis lupus familiaris OX=9615 GN=LCNL1 PE=3 SV=2	2,55	2,39	372,70	2669779,5	9	1	0	3,68	299	1932
>tr|E2R4X9|E2R4X9_CANLF Pre-mRNA processing factor 8 OS=Canis lupus familiaris OX=9615 GN=PRPF8 PE=4 SV=2	0,20	0,20	77,40	2666423,5	1	1	0	0,34	2335	29235
>tr|A0A5F4D6N0|A0A5F4D6N0_CANLF IF rod domain-containing protein OS=Canis lupus familiaris OX=9615 PE=3 SV=1	0,55	0,55	227,40	2567902,8	1	1	1	7,06	439	38799
>tr|A0A5F4DGF5|A0A5F4DGF5_CANLF Alkaline phosphatase OS=Canis lupus familiaris OX=9615 GN=ALPL PE=3 SV=1	1,07	1,05	205,70	2494054,1	2	1	0	1,92	572	6357
>tr|A0A5F4CWZ1|A0A5F4CWZ1_CANLF Nuclear receptor subfamily 3 group C member 2 OS=Canis lupus familiaris OX=9615 GN=NR3C2 PE=4 SV=1	0,97	0,97	258,30	2492161,0	1	1	1	2,43	947	33679
>tr|J9NS29|J9NS29_CANLF Cystatin domain-containing protein OS=Canis lupus familiaris OX=9615 GN=LOC607874 PE=4 SV=2	1,00	0,96	337,10	2483724,5	3	1	0	9,58	313	30016
>tr|E2RCT1|E2RCT1_CANLF WAP domain-containing protein OS=Canis lupus familiaris OX=9615 PE=4 SV=2	3,68	3,68	248,90	2350865,6	1	1	0	13,79	116	21717
>tr|F1PB68|F1PB68_CANLF Olfactomedin 4 OS=Canis lupus familiaris OX=9615 GN=OLFM4 PE=4 SV=3	1,32	1,32	256,10	2328468,0	1	1	0	4,40	477	17246
>tr|F6Y1C9|F6Y1C9_CANLF DNA polymerase OS=Canis lupus familiaris OX=9615 GN=POLA1 PE=3 SV=1	0,83	0,83	111,00	2328468,0	1	1	0	1,70	1467	1834
>tr|E2R6E0|E2R6E0_CANLF Lipocln_cytosolic_FA-bd_dom domain-containing protein OS=Canis lupus familiaris OX=9615 GN=LCNL1 PE=3 SV=2	3,43	2,44	314,30	2316264,1	2	2	0	7,36	299	1932
>tr|F1PZI2|F1PZI2_CANLF RING-type E3 ubiquitin transferase OS=Canis lupus familiaris OX=9615 GN=MIB2 PE=4 SV=3	0,88	0,80	235,50	2279261,5	5	1	0	1,19	1005	7737
>sp|P25291|GP2_CANLF Pancreatic secretory granule membrane major glycoprotein GP2 OS=Canis lupus familiaris OX=9615 GN=GP2 PE=1 SV=1	0,13	0,13	168,80	2219087,2	1	1	0	2,36	509	142
>tr|A0A5F4DGF5|A0A5F4DGF5_CANLF Alkaline phosphatase OS=Canis lupus familiaris OX=9615 GN=ALPL PE=3 SV=1	1,08	1,08	177,20	2217922,9	1	1	0	1,92	572	6357
>tr|A0A5F4BT11|A0A5F4BT11_CANLF Ecdysoneless cell cycle regulator OS=Canis lupus familiaris OX=9615 GN=ECD PE=4 SV=1	0,13	0,13	127,10	2211908,5	1	1	0	1,52	656	8613
>tr|J9JHE0|J9JHE0_CANLF cGMP-dependent protein kinase OS=Canis lupus familiaris OX=9615 GN=PRKG1 PE=3 SV=2	0,13	0,14	78,50	2210663,8	1	1	0	2,68	671	5787
>tr|E2R5P5|E2R5P5_CANLF Calcyclin OS=Canis lupus familiaris OX=9615 GN=S100A8 PE=4 SV=2	1,00	0,98	203,80	2184448,7	2	1	0	14,66	191	27843

>tr|A0A5F4C379|A0A5F4C379_CANLF Protein kinase C OS=Canis lupus familiaris OX=9615 GN=PRKD2 PE=3 SV=1	0,20	0,20	30,40	2174936,4	1	1	0	0,49	1018	9891
>tr|E2R0Z6|E2R0Z6_CANLF Junctophilin OS=Canis lupus familiaris OX=9615 GN=JPH1 PE=3 SV=1	0,27	0,25	50,00	2163667,0	2	1	0	1,06	662	32945
>tr|F1Q0I8|F1Q0I8_CANLF Glutathione transferase OS=Canis lupus familiaris OX=9615 GN=LOC476006 PE=4 SV=3	0,10	0,09	79,10	2143810,1	1	1	0	9,96	241	8185
>tr|F1Q074|F1Q074_CANLF A-kinase anchoring protein 13 OS=Canis lupus familiaris OX=9615 GN=AKAP13 PE=4 SV=3	0,67	0,59	187,30	2124844,0	5	1	0	0,29	2781	14817
>tr|E2R0Z6|E2R0Z6_CANLF Junctophilin OS=Canis lupus familiaris OX=9615 GN=JPH1 PE=3 SV=1	1,01	0,99	61,00	2087695,2	2	1	0	1,06	662	32945
>tr|F1PRL1|F1PRL1_CANLF Dppa2_A domain-containing protein OS=Canis lupus familiaris OX=9615 PE=4 SV=2	0,36	0,20	54,60	2073501,6	9	1	0	2,79	179	17222
>tr|F1PGP5|F1PGP5_CANLF Argonaute RISC component 1 OS=Canis lupus familiaris OX=9615 GN=AGO1 PE=3 SV=3	0,38	0,38	203,20	2071553,1	1	1	0	1,25	1037	5034
>tr|A0A5F4CKD5|A0A5F4CKD5_CANLF Polypeptide N-acetylgalactosaminyltransferase OS=Canis lupus familiaris OX=9615 GN=GALNT6 PE=3 SV=1	0,43	0,44	222,30	2056818,1	1	1	0	4,97	644	1617
>tr|E2RQC4|E2RQC4_CANLF WW domain-containing oxidoreductase OS=Canis lupus familiaris OX=9615 GN=WWOX PE=4 SV=3	0,11	0,09	133,30	2054856,1	2	1	0	2,05	391	23035
>tr|A0A5F4CCD0|A0A5F4CCD0_CANLF Cysteine rich secretory protein 2 OS=Canis lupus familiaris OX=9615 GN=CRISP2 PE=3 SV=1	2,89	2,46	379,70	2033646,3	2	2	1	16,40	311	11017
>tr|A0A5F4C2Y3|A0A5F4C2Y3_CANLF Carboxypeptidase X, M14 family member 1 OS=Canis lupus familiaris OX=9615 GN=CPXM1 PE=3 SV=1	0,25	0,23	61,60	2024378,6	2	1	0	1,21	661	21027
>sp|O46607|GPX5_CANLF Epididymal secretory glutathione peroxidase OS=Canis lupus familiaris OX=9615 GN=GPX5 PE=2 SV=1	0,90	0,84	263,30	2007399,4	4	1	0	6,79	221	564
>tr|A0A5F4CDM1|A0A5F4CDM1_CANLF L-lactate dehydrogenase OS=Canis lupus familiaris OX=9615 PE=3 SV=1	0,50	0,48	109,10	1966922,5	2	1	0	2,87	279	43073
>tr|F6XBC9|F6XBC9_CANLF Olfactory receptor OS=Canis lupus familiaris OX=9615 GN=LOC607447 PE=3 SV=2	0,18	0,18	60,90	1966414,9	1	1	0	4,81	312	3183
>tr|A0A5F4DKA1|A0A5F4DKA1_CANLF Zinc finger protein 335 OS=Canis lupus familiaris OX=9615 GN=ZNF335 PE=4 SV=1	0,12	0,12	64,50	1925598,9	1	1	0	1,59	1380	32203
>tr|A0A5F4CCD0|A0A5F4CCD0_CANLF Cysteine rich secretory protein 2 OS=Canis lupus familiaris OX=9615 GN=CRISP2 PE=3 SV=1	3,74	3,66	351,10	1907787,9	5	1	0	4,82	311	11017
>sp|B6V8E6|CTNB1_CANLF Catenin beta-1 OS=Canis lupus familiaris OX=9615 GN=CTNNB1 PE=1 SV=1	0,10	0,10	122,70	1897586,5	1	1	0	0,51	781	442
>tr|J9NUX6|J9NUX6_CANLF Protein phosphatase 6 regulatory subunit 2 OS=Canis lupus familiaris OX=9615 GN=PPP6R2 PE=3 SV=2	0,10	0,10	251,30	1884140,0	1	1	0	5,59	930	3081
>tr|F1PS33|F1PS33_CANLF Otogelin OS=Canis lupus familiaris OX=9615 GN=OTOG PE=4 SV=3	0,15	0,14	162,40	1802840,6	2	2	2	1,67	2876	38364
>sp|Q9GL25|ESPB1_CANLF Epididymal sperm-binding protein 1 OS=Canis lupus familiaris OX=9615 GN=ELSPBP1 PE=1 SV=1	6,20	3,06	299,00	1777423,2	7	2	0	6,94	245	36
>tr|J9NS29|J9NS29_CANLF Cystatin domain-containing protein OS=Canis lupus familiaris OX=9615 GN=LOC607874 PE=4 SV=2	0,34	0,32	175,00	1762960,3	2	1	0	9,58	313	30016
>tr|A0A5F4C3M5|A0A5F4C3M5_CANLF IQ motif containing GTPase activating protein 2 OS=Canis lupus familiaris OX=9615 GN=IQGAP2 PE=4 SV=1	0,87	0,81	107,00	1742818,9	4	1	0	0,51	1577	1056

>sp|Q6AW47|EST5A_CANLF Carboxylesterase 5A OS=Canis lupus familiaris OX=9615 GN=CES5A PE=2 SV=1	3,14	3,12	351,30	1724592,0	2	1	0	3,65	575	629
>tr|E2R0Z6|E2R0Z6_CANLF Junctophilin OS=Canis lupus familiaris OX=9615 GN=JPH1 PE=3 SV=1	0,21	0,19	95,60	1700379,7	2	1	0	1,06	662	32945
>tr|F1PB83|F1PB83_CANLF Integrin subunit alpha 9 OS=Canis lupus familiaris OX=9615 GN=ITGA9 PE=3 SV=3	0,12	0,12	147,90	1696053,5	1	1	0	1,03	975	39570
>tr|F6XVF5|F6XVF5_CANLF WD repeat domain 72 OS=Canis lupus familiaris OX=9615 GN=WDR72 PE=4 SV=2	1,08	1,08	120,10	1689397,3	1	1	0	0,75	1071	16644
>tr|A0A5F4CS14|A0A5F4CS14_CANLF Guanylate cyclase OS=Canis lupus familiaris OX=9615 GN=GUCY1A2 PE=3 SV=1	0,39	0,37	147,30	1685659,3	2	1	0	1,03	773	1560
>tr|E2RCT1|E2RCT1_CANLF WAP domain-containing protein OS=Canis lupus familiaris OX=9615 PE=4 SV=2	3,04	3,00	392,80	1662777,2	3	1	0	13,79	116	21717
>tr|F1PBA6|F1PBA6_CANLF HIVEP zinc finger 1 OS=Canis lupus familiaris OX=9615 GN=HIVEP1 PE=4 SV=2	0,25	0,25	276,50	1627631,4	1	1	1	1,61	2726	6985
>tr|J9NSV2|J9NSV2_CANLF Olfactory receptor OS=Canis lupus familiaris OX=9615 GN=OR5AC27 PE=3 SV=2	0,20	0,18	43,10	1612760,8	2	1	0	5,88	306	42329
>tr|F6XXH4|F6XXH4_CANLF Transmembrane channel-like protein OS=Canis lupus familiaris OX=9615 GN=TMC4 PE=3 SV=1	0,77	0,77	239,20	1516687,0	1	1	0	2,72	736	31723
>sp|Q6AW47|EST5A_CANLF Carboxylesterase 5A OS=Canis lupus familiaris OX=9615 GN=CES5A PE=2 SV=1	2,38	2,36	282,60	1499423,9	2	1	0	3,65	575	629
>tr|A0A5F4BXF4|A0A5F4BXF4_CANLF Signal transducer and activator of transcription OS=Canis lupus familiaris OX=9615 GN=STAT6 PE=3 SV=1	0,81	0,79	99,30	1490042,4	2	1	0	0,86	927	29502
>tr|A0A5F4D3Q2|A0A5F4D3Q2_CANLF Non-specific serine/threonine protein kinase OS=Canis lupus familiaris OX=9615 GN=ATR PE=3 SV=1	0,12	0,12	143,90	1479485,4	1	1	0	0,39	2583	7077
>tr|E2QZP6|E2QZP6_CANLF Tectorin alpha OS=Canis lupus familiaris OX=9615 GN=TECTA PE=4 SV=1	0,20	0,20	143,40	1471824,2	1	1	0	2,09	2155	8407
>tr|E2RPJ0|E2RPJ0_CANLF B9 domain containing 1 OS=Canis lupus familiaris OX=9615 GN=B9D1 PE=4 SV=2	0,33	0,33	125,60	1443655,7	1	1	0	6,82	337	3998
>tr|A0A5F4CS14|A0A5F4CS14_CANLF Guanylate cyclase OS=Canis lupus familiaris OX=9615 GN=GUCY1A2 PE=3 SV=1	0,89	0,89	143,50	1433965,6	1	1	0	1,03	773	1560
>tr|E2R6E0|E2R6E0_CANLF Lipocln_cytosolic_FA-bd_dom domain-containing protein OS=Canis lupus familiaris OX=9615 GN=LCNL1 PE=3 SV=2	2,09	2,03	333,60	1425748,0	4	1	0	3,68	299	1932
>tr|F1PIZ1|F1PIZ1_CANLF Caspase recruitment domain family member 6 OS=Canis lupus familiaris OX=9615 GN=CARD6 PE=4 SV=3	1,04	1,04	134,10	1402703,6	1	1	0	0,65	1071	14260
>tr|J9PA59|J9PA59_CANLF Olfactory receptor OS=Canis lupus familiaris OX=9615 GN=OR4D9D PE=3 SV=1	0,80	0,80	288,00	1391820,7	1	1	1	7,40	311	36467
>tr|J9NWY1|J9NWY1_CANLF Quinoid dihydropteridine reductase OS=Canis lupus familiaris OX=9615 GN=QDPR PE=3 SV=2	1,24	1,22	180,10	1388401,2	2	1	0	6,42	296	22710
>tr|E2R9H5|E2R9H5_CANLF Na(+)-dependent phosphate cotransporter 2B OS=Canis lupus familiaris OX=9615 GN=SLC34A2 PE=3 SV=3	0,17	0,17	105,40	1378968,3	1	1	0	1,52	724	1185
>sp|Q8WNN6|SODC_CANLF Superoxide dismutase [Cu-Zn] OS=Canis lupus familiaris OX=9615 GN=SOD1 PE=2 SV=1	1,72	1,70	331,30	1369830,7	2	1	0	15,69	153	6
>tr|E2QUV3|E2QUV3_CANLF Alpha-2-HS-glycoprotein OS=Canis lupus familiaris OX=9615 GN=AHSG PE=4 SV=2	1,70	1,70	431,30	1348281,7	1	1	0	8,49	365	20747
>tr|E2RL47|E2RL47_CANLF Protocadherin 17 OS=Canis lupus familiaris	0,76	0,76	140,20	1314294,0	1	1	0	0,87	1154	27313

OX=9615 GN=PCDH17 PE=4 SV=3										
>tr|E2RG75|E2RG75_CANLF Inactive ribonuclease-like protein 9 OS=Canis lupus familiaris OX=9615 GN=RNASE9 PE=3 SV=2	2,50	2,48	307,30	1290137,0	2	1	0	7,07	198	41734
>tr|A0A5F4CKD5|A0A5F4CKD5_CANLF Polypeptide N-acetylgalactosaminyltransferase OS=Canis lupus familiaris OX=9615 GN=GALNT6 PE=3 SV=1	1,67	1,65	308,40	1284833,2	2	1	0	4,97	644	1617
>sp|Q9GL25|ESPB1_CANLF Epididymal sperm-binding protein 1 OS=Canis lupus familiaris OX=9615 GN=ELSPBP1 PE=1 SV=1	3,05	2,97	228,50	1270739,3	5	1	0	6,94	245	36
>tr|E2RG75|E2RG75_CANLF Inactive ribonuclease-like protein 9 OS=Canis lupus familiaris OX=9615 GN=RNASE9 PE=3 SV=2	3,09	3,03	364,60	1247887,5	4	1	0	7,07	198	41734
>tr|F1P655|F1P655_CANLF Purinergic receptor P2Y13 OS=Canis lupus familiaris OX=9615 GN=P2RY13 PE=3 SV=3	0,28	0,28	148,50	1221463,3	1	1	1	7,04	341	4337
>tr|J9NZ25|J9NZ25_CANLF Shortage in chiasmata 1 OS=Canis lupus familiaris OX=9615 GN=SHOC1 PE=4 SV=2	0,13	0,13	146,60	1194939,3	1	1	1	2,52	1428	1783
>tr|A0A5F4CCD0|A0A5F4CCD0_CANLF Cysteine rich secretory protein 2 OS=Canis lupus familiaris OX=9615 GN=CRISP2 PE=3 SV=1	5,06	3,70	502,40	1192008,1	2	2	1	16,40	311	11017
>tr|E2RSA6|E2RSA6_CANLF Catenin beta like 1 OS=Canis lupus familiaris OX=9615 GN=CTNNBL1 PE=4 SV=2	0,96	0,96	126,90	1179921,7	1	1	0	1,42	563	42500
>tr|J9P887|J9P887_CANLF Vascular endothelial zinc finger 1 OS=Canis lupus familiaris OX=9615 GN=VEZF1 PE=4 SV=2	0,10	0,08	133,90	1167339,4	2	2	2	3,37	504	1015
>tr|A0A5F4CNT7|A0A5F4CNT7_CANLF Phosphatidylinositol glycan anchor biosynthesis class G OS=Canis lupus familiaris OX=9615 GN=PIGG PE=4 SV=1	0,26	0,26	142,90	1159811,4	1	1	1	1,06	850	6631
>tr|A0A5F4C6B5|A0A5F4C6B5_CANLF Plastin 3 OS=Canis lupus familiaris OX=9615 GN=PLS3 PE=4 SV=1	0,73	0,73	236,50	1137141,3	1	1	0	4,07	639	30730
>tr|F1PZI2|F1PZI2_CANLF RING-type E3 ubiquitin transferase OS=Canis lupus familiaris OX=9615 GN=MIB2 PE=4 SV=3	0,12	0,10	237,70	1135441,9	2	1	0	1,19	1005	7737
>tr|J9NS29|J9NS29_CANLF Cystatin domain-containing protein OS=Canis lupus familiaris OX=9615 GN=LOC607874 PE=4 SV=2	0,23	0,23	193,60	1127289,5	1	1	0	4,79	313	30016
>tr|A0A5F4CPK4|A0A5F4CPK4_CANLF Formin binding protein 1 OS=Canis lupus familiaris OX=9615 GN=FNBP1 PE=4 SV=1	0,20	0,20	92,90	1126990,3	1	1	0	1,42	562	14071
>tr|A0A5F4DFY8|A0A5F4DFY8_CANLF Splicing factor 3b subunit 3 OS=Canis lupus familiaris OX=9615 GN=SF3B3 PE=4 SV=1	0,21	0,21	257,30	1126487,9	1	1	1	1,94	1185	5042
>tr|J9NS29|J9NS29_CANLF Cystatin domain-containing protein OS=Canis lupus familiaris OX=9615 GN=LOC607874 PE=4 SV=2	2,22	2,22	255,00	1118240,4	1	1	0	4,79	313	30016
>tr|A0A5F4CDM1|A0A5F4CDM1_CANLF L-lactate dehydrogenase OS=Canis lupus familiaris OX=9615 PE=3 SV=1	0,83	0,81	130,30	1099498,5	2	1	0	2,87	279	43073
>tr|A0A5F4CDI3|A0A5F4CDI3_CANLF Dynein axonemal intermediate chain 4 OS=Canis lupus familiaris OX=9615 GN=DNAI4 PE=4 SV=1	0,11	0,11	113,20	1082266,0	1	1	0	2,78	791	31982
>tr|F1PDB9|F1PDB9_CANLF Toll like receptor 4 OS=Canis lupus familiaris OX=9615 GN=TLR4 PE=3 SV=2	0,77	0,78	66,90	1069260,0	1	1	0	0,96	833	36972
>tr|A0A5F4CZB6|A0A5F4CZB6_CANLF Acetyl-coenzyme A synthetase OS=Canis lupus familiaris OX=9615 GN=ACSS1 PE=3 SV=1	0,16	0,16	72,30	1064814,9	1	1	0	0,89	900	28765
>tr|A0A5F4CUJ1|A0A5F4CUJ1_CANLF Protein tyrosine phosphatase 4A2 OS=Canis lupus familiaris OX=9615 GN=PTP4A2 PE=4 SV=1	0,26	0,26	148,00	1045484,0	1	1	0	9,86	142	9003

>tr|J9P432|J9P432_CANLF Glutamine--fructose-6-phosphate transaminase (isomerizing) OS=Canis lupus familiaris OX=9615 GN=GFPT1 PE=4 SV=2	0,81	0,81	173,90	1035497,0	1	1	0	1,18	677	7191
>tr|J9NRX4|J9NRX4_CANLF Glutathione transferase OS=Canis lupus familiaris OX=9615 GN=LOC481841 PE=3 SV=2	2,84	2,84	390,80	967231,6	1	1	0	10,98	246	15581
>tr|A0A5F4C3Q4|A0A5F4C3Q4_CANLF Polypyrimidine tract binding protein 2 OS=Canis lupus familiaris OX=9615 GN=PTBP2 PE=4 SV=1	0,78	0,78	140,80	952806,8	1	1	0	2,25	356	10982
>tr|F1PNH7|F1PNH7_CANLF Cartilage intermediate layer protein 2 OS=Canis lupus familiaris OX=9615 GN=CILP2 PE=4 SV=3	0,10	0,09	150,90	936344,1	1	1	0	6,27	303	1537
>tr|F1PZI2|F1PZI2_CANLF RING-type E3 ubiquitin transferase OS=Canis lupus familiaris OX=9615 GN=MIB2 PE=4 SV=3	0,80	0,78	209,30	928079,6	2	1	0	1,19	1005	7737
>tr|A0A5F4CS14|A0A5F4CS14_CANLF Guanylate cyclase OS=Canis lupus familiaris OX=9615 GN=GUCY1A2 PE=3 SV=1	0,48	0,46	97,60	904886,3	2	1	0	1,03	773	1560
>tr|E2RIN3|E2RIN3_CANLF Otoancorin OS=Canis lupus familiaris OX=9615 GN=OTOA PE=3 SV=3	0,25	0,25	98,10	851351,4	1	1	0	0,70	1139	18241
>sp|Q9GL25|ESPB1_CANLF Epididymal sperm-binding protein 1 OS=Canis lupus familiaris OX=9615 GN=ELSPBP1 PE=1 SV=1	2,75	2,69	213,70	798630,0	4	1	0	6,94	245	36
>tr|J9P366|J9P366_CANLF Ganglioside induced differentiation associated protein 2 OS=Canis lupus familiaris OX=9615 GN=GDAP2 PE=3 SV=2	0,19	0,19	75,00	783748,4	1	1	0	7,44	497	9162
>tr|F6XVF5|F6XVF5_CANLF WD repeat domain 72 OS=Canis lupus familiaris OX=9615 GN=WDR72 PE=4 SV=2	0,53	0,53	80,40	749247,3	1	1	0	0,75	1071	16644
>tr|A0A5F4BUE2|A0A5F4BUE2_CANLF SpoU_methylase domain-containing protein OS=Canis lupus familiaris OX=9615 GN=TARBP1 PE=4 SV=1	1,15	1,15	143,70	699101,6	1	1	0	0,50	1602	2815
>tr|J9P2E5|J9P2E5_CANLF PPFIA binding protein 1 OS=Canis lupus familiaris OX=9615 GN=PPFIBP1 PE=3 SV=2	1,30	1,30	159,40	697082,8	1	1	0	1,39	1005	1621
>tr|E2R6E0|E2R6E0_CANLF Lipocln_cytosolic_FA-bd_dom domain-containing protein OS=Canis lupus familiaris OX=9615 GN=LCNL1 PE=3 SV=2	1,94	1,90	281,50	683544,2	3	1	0	3,68	299	1932
>tr|E2QWJ0|E2QWJ0_CANLF Poly [ADP-ribose] polymerase OS=Canis lupus familiaris OX=9615 GN=PARP4 PE=4 SV=3	0,27	0,28	189,90	683312,6	1	1	0	2,01	1688	34837
>tr|A0A5F4CNW2|A0A5F4CNW2_CANLF Radical S-adenosyl methionine and flavodoxin domain-containing protein 1 OS=Canis lupus familiaris OX=9615 GN=LOC479708 PE=3 SV=1	0,13	0,14	57,00	680820,4	1	1	1	1,04	670	13683
>tr|A0A5F4C605|A0A5F4C605_CANLF Centrosomal protein 78 OS=Canis lupus familiaris OX=9615 GN=CEP78 PE=4 SV=1	0,36	0,37	76,50	669064,1	1	1	0	0,87	686	7979
>tr|A0A5F4DK72|A0A5F4DK72_CANLF Nuclear factor 1 OS=Canis lupus familiaris OX=9615 GN=NFIA PE=3 SV=1	0,21	0,21	97,20	661679,6	1	1	0	3,97	478	11874
>tr|F1PIZ1|F1PIZ1_CANLF Caspase recruitment domain family member 6 OS=Canis lupus familiaris OX=9615 GN=CARD6 PE=4 SV=3	0,76	0,75	86,00	645761,3	2	1	0	0,65	1071	14260
>sp|Q28894|WFDC2_CANLF WAP four-disulfide core domain protein 2 OS=Canis lupus familiaris OX=9615 GN=WFDC2 PE=2 SV=1	0,98	0,96	109,00	612111,6	2	1	0	12,90	124	53
>tr|A0A5F4CLR5|A0A5F4CLR5_CANLF Ligand dependent nuclear receptor corepressor like OS=Canis lupus familiaris OX=9615 GN=LCORL PE=4 SV=1	0,10	0,09	53,80	602155,3	1	1	0	2,84	634	1650
>tr|A0A5F4D4V6|A0A5F4D4V6_CANLF Ankyrin repeat domain 52 OS=Canis lupus familiaris OX=9615 GN=ANKRD52 PE=4 SV=1	0,36	0,36	135,50	595276,9	1	1	0	1,87	1067	6228
>tr|J9P4P2|J9P4P2_CANLF Cation-transporting ATPase OS=Canis lupus familiaris OX=9615 GN=ATP13A3 PE=3 SV=1	0,11	0,12	78,70	581572,2	1	1	0	1,43	1257	1873

>tr|A0A5F4DEU9|A0A5F4DEU9_CANLF Dehydrogenase/reductase 3 OS=Canis lupus familiaris OX=9615 GN=DHRS3 PE=3 SV=1	0,10	0,10	38,40	573898,8	1	1	0	2,79	323	16256
>tr|F1PZB3|F1PZB3_CANLF PRAME nuclear receptor transcriptional regulator OS=Canis lupus familiaris OX=9615 GN=PRAME PE=3 SV=2	1,08	1,08	72,20	565593,9	1	1	0	3,32	512	30492
>tr|A0A5F4CCJ1|A0A5F4CCJ1_CANLF Transmembrane protein 241 OS=Canis lupus familiaris OX=9615 GN=TMEM241 PE=4 SV=1	0,16	0,16	202,90	558332,0	1	1	0	3,75	347	27419
>tr|E2RG75|E2RG75_CANLF Inactive ribonuclease-like protein 9 OS=Canis lupus familiaris OX=9615 GN=RNASE9 PE=3 SV=2	1,32	1,31	172,50	532308,8	2	1	0	7,07	198	41734
>tr|F1PXA2|F1PXA2_CANLF Delta(24)-sterol reductase OS=Canis lupus familiaris OX=9615 GN=DHCR24 PE=4 SV=2	0,11	0,11	95,10	528394,0	1	1	0	1,74	516	37097
>tr|F1PP49|F1PP49_CANLF Transmembrane protein 8B OS=Canis lupus familiaris OX=9615 GN=TMEM8B PE=3 SV=3	0,97	0,97	62,00	528354,6	1	1	0	2,02	891	36725
>sp|Q8MJ44|KAPCA_CANLF cAMP-dependent protein kinase catalytic subunit alpha OS=Canis lupus familiaris OX=9615 GN=PRKACA PE=1 SV=3	0,57	0,57	117,20	525580,8	1	1	0	4,86	350	405
>tr|A0A5F4BZ56|A0A5F4BZ56_CANLF Chymotrypsin-like elastase family member 1 OS=Canis lupus familiaris OX=9615 GN=CELA1 PE=4 SV=1	0,72	0,72	36,40	522309,6	1	1	0	2,15	372	21422
>tr|A0A5F4C6U7|A0A5F4C6U7_CANLF Early endosome antigen 1 OS=Canis lupus familiaris OX=9615 GN=EEA1 PE=4 SV=1	0,95	0,95	109,90	513140,4	1	1	0	0,51	1369	15937
>tr|A0A5F4C3M5|A0A5F4C3M5_CANLF IQ motif containing GTPase activating protein 2 OS=Canis lupus familiaris OX=9615 GN=IQGAP2 PE=4 SV=1	0,79	0,79	86,80	506931,0	1	1	0	0,51	1577	1056
>tr|A0A5F4CFB6|A0A5F4CFB6_CANLF Potassium voltage-gated channel interacting protein 3 OS=Canis lupus familiaris OX=9615 GN=KCNIP3 PE=3 SV=1	0,23	0,23	99,80	502610,4	1	1	0	1,84	708	2187
>sp|E2RDV1|TDRD7_CANLF Tudor domain-containing protein 7 OS=Canis lupus familiaris OX=9615 GN=TDRD7 PE=3 SV=1	1,07	1,07	40,20	498533,8	1	1	0	0,71	1125	54
>tr|J9P748|J9P748_CANLF Bromodomain adjacent to zinc finger domain 1A OS=Canis lupus familiaris OX=9615 GN=BAZ1A PE=4 SV=1	0,13	0,13	110,70	489844,5	1	1	1	0,71	1557	10939
>tr|J9NXF6|J9NXF6_CANLF Pleckstrin homology and RhoGEF domain containing G5 OS=Canis lupus familiaris OX=9615 GN=PLEKHG5 PE=4 SV=1	0,12	0,12	110,30	487039,7	1	1	0	1,25	1043	9267
>tr|F1PY29|F1PY29_CANLF Olfactory receptor OS=Canis lupus familiaris OX=9615 GN=OR6X1 PE=3 SV=3	0,51	0,51	99,50	486152,3	1	1	0	7,37	312	12603
>tr|F1PEZ5|F1PEZ5_CANLF Laminin subunit alpha 2 OS=Canis lupus familiaris OX=9615 GN=LAMA2 PE=4 SV=3	0,39	0,39	35,20	481210,0	1	1	0	0,51	3112	8576
>tr|A0A5F4CCX4|A0A5F4CCX4_CANLF UHRF1 binding protein 1 OS=Canis lupus familiaris OX=9615 GN=UHRF1BP1 PE=4 SV=1	0,60	0,60	88,10	467957,5	1	1	0	0,77	1430	4238
>tr|A0A5F4CCJ1|A0A5F4CCJ1_CANLF Transmembrane protein 241 OS=Canis lupus familiaris OX=9615 GN=TMEM241 PE=4 SV=1	0,83	0,81	176,00	453167,6	2	1	0	3,75	347	27419
>tr|A0A5F4BVH6|A0A5F4BVH6_CANLF KIAA1549 OS=Canis lupus familiaris OX=9615 GN=KIAA1549 PE=4 SV=1	0,18	0,18	201,10	450870,1	1	1	0	0,50	1793	5440
>tr|E2RR30|E2RR30_CANLF Fatty acid amide hydrolase OS=Canis lupus familiaris OX=9615 GN=FAAH PE=3 SV=2	0,73	0,73	40,10	441460,9	1	1	0	1,38	579	1550
>tr|F1PHM8|F1PHM8_CANLF Hexosyltransferase OS=Canis lupus familiaris OX=9615 GN=B3GNT4 PE=3 SV=2	0,25	0,25	58,70	433737,0	1	1	0	2,24	357	33620
>tr|E2RI00|E2RI00_CANLF Tetratricopeptide repeat domain 5 OS=Canis lupus familiaris OX=9615 GN=TTC5 PE=4 SV=2	0,22	0,22	137,20	416505,0	1	1	0	4,56	439	12220
>tr|F1PPJ8|F1PPJ8_CANLF Hephaestin like 1 OS=Canis lupus familiaris	0,44	0,44	43,90	415660,3	1	1	0	1,33	1204	16608

OX=9615 GN=HEPHL1 PE=3 SV=3										
>tr|F1Q1L4|F1Q1L4_CANLF Chromosome 6 C16orf89 homolog OS=Canis lupus familiaris OX=9615 GN=C6H16orf89 PE=4 SV=2	1,50	1,50	167,80	394147,4	1	1	0	3,49	373	20319
>sp|Q28895|NPC2_CANLF NPC intracellular cholesterol transporter 2 OS=Canis lupus familiaris OX=9615 GN=NPC2 PE=2 SV=1	2,78	2,74	288,10	394021,3	3	1	0	8,72	149	153
>tr|A0A5F4C8F3|A0A5F4C8F3_CANLF Ankyrin 1 OS=Canis lupus familiaris OX=9615 GN=ANK1 PE=4 SV=1	0,95	0,95	85,50	380540,6	1	1	0	0,43	1860	11424
>tr|A0A5F4CKH6|A0A5F4CKH6_CANLF Reverse transcriptase domain-containing protein OS=Canis lupus familiaris OX=9615 PE=4 SV=1	0,44	0,44	108,30	374501,3	1	1	0	0,80	1001	26258
>tr|E2RSH4|E2RSH4_CANLF Sushi domain containing 2 OS=Canis lupus familiaris OX=9615 GN=SUSD2 PE=4 SV=3	0,33	0,33	107,30	360226,2	1	1	0	1,76	795	4865
>tr|A0A5F4C8B1|A0A5F4C8B1_CANLF Glycine cleavage system P protein OS=Canis lupus familiaris OX=9615 GN=GLDC PE=3 SV=1	0,18	0,18	190,20	359608,6	1	1	0	1,08	1023	10505
>tr|A0A5F4CPG8|A0A5F4CPG8_CANLF TM2 domain containing 3 OS=Canis lupus familiaris OX=9615 GN=TM2D3 PE=4 SV=1	0,19	0,19	151,30	357374,8	1	1	1	7,73	220	4691
>tr|E2RPQ4|E2RPQ4_CANLF FAST kinase domains 3 (Fragment) OS=Canis lupus familiaris OX=9615 GN=FASTKD3 PE=4 SV=3	0,10	0,09	88,20	347444,8	1	1	1	2,54	590	17330
>tr|A0A5F4C296|A0A5F4C296_CANLF Valyl-tRNA synthetase OS=Canis lupus familiaris OX=9615 GN=VARS1 PE=3 SV=1	0,11	0,11	78,00	346450,8	1	1	0	0,61	1320	1432
>tr|A0A5F4C407|A0A5F4C407_CANLF V-type proton ATPase subunit a OS=Canis lupus familiaris OX=9615 GN=ATP6V0A1 PE=3 SV=1	0,24	0,24	91,60	323166,1	1	1	0	0,82	857	4387
>tr|A0A5F4C1Y7|A0A5F4C1Y7_CANLF Anoctamin OS=Canis lupus familiaris OX=9615 GN=ANO10 PE=3 SV=1	0,10	0,10	80,70	323166,1	1	1	0	1,09	644	19257
>tr|F1PUE6|F1PUE6_CANLF SUMO specific peptidase 1 OS=Canis lupus familiaris OX=9615 GN=SENP1 PE=3 SV=2	1,23	1,23	94,00	318375,9	1	1	0	1,01	690	7863
>sp|Q28894|WFDC2_CANLF WAP four-disulfide core domain protein 2 OS=Canis lupus familiaris OX=9615 GN=WFDC2 PE=2 SV=1	0,89	0,89	101,70	298881,1	1	1	0	12,90	124	53
>tr|A0A5F4CCJ1|A0A5F4CCJ1_CANLF Transmembrane protein 241 OS=Canis lupus familiaris OX=9615 GN=TMEM241 PE=4 SV=1	0,72	0,72	202,80	293469,0	1	1	0	3,75	347	27419
>tr|A0A5F4CAX4|A0A5F4CAX4_CANLF Myeloid cell nuclear differentiation antigen OS=Canis lupus familiaris OX=9615 GN=MNDA PE=4 SV=1	0,73	0,73	96,80	291913,1	1	1	0	2,02	397	2551
>tr|A0A5F4DH95|A0A5F4DH95_CANLF F-actin monooxygenase OS=Canis lupus familiaris OX=9615 GN=MICAL1 PE=3 SV=1	0,25	0,25	62,60	291417,0	1	1	0	1,57	1017	11153
>tr|J9P673|J9P673_CANLF Nicotinamide N-methyltransferase OS=Canis lupus familiaris OX=9615 GN=NNMT PE=3 SV=1	0,12	0,12	66,10	287425,1	1	1	0	7,95	264	25034
>tr|A0A5F4CLX5|A0A5F4CLX5_CANLF Nucleolar complex associated 4 homolog OS=Canis lupus familiaris OX=9615 GN=NOC4L PE=3 SV=1	0,75	0,75	98,30	278863,1	1	1	0	1,38	581	12985
>tr|E2R9A2|E2R9A2_CANLF Coatomer subunit beta OS=Canis lupus familiaris OX=9615 GN=COPB1 PE=4 SV=2	0,38	0,38	80,70	278075,0	1	1	1	0,84	953	33386
>tr|A0A5F4D153|A0A5F4D153_CANLF Calcium/calmodulin-dependent protein kinase OS=Canis lupus familiaris OX=9615 GN=CAMK2G PE=3 SV=1	0,12	0,12	89,40	270937,9	1	1	1	2,37	590	17859
>tr|A0A5F4D8W3|A0A5F4D8W3_CANLF Vitamin K-dependent protein S OS=Canis lupus familiaris OX=9615 GN=PROS1 PE=4 SV=1	0,72	0,72	55,60	269246,9	1	1	0	1,58	505	1354
>tr|F1PIZ1|F1PIZ1_CANLF Caspase recruitment domain family member 6 OS=Canis lupus familiaris OX=9615 GN=CARD6 PE=4 SV=3	0,35	0,35	48,70	268603,6	1	1	0	0,65	1071	14260

>tr|A0A5F4DLD7|A0A5F4DLD7_CANLF Ig-like domain-containing protein OS=Canis lupus familiaris OX=9615 GN=LOC484343 PE=4 SV=1	0,13	0,13	46,80	267285,9	1	1	0	3,39	443	36632
>tr|A0A5F4BQ43|A0A5F4BQ43_CANLF ATP binding cassette subfamily G member 5 OS=Canis lupus familiaris OX=9615 GN=ABCG5 PE=3 SV=1	0,12	0,12	50,40	249290,4	1	1	0	2,23	628	8651
>tr|F6XVF5|F6XVF5_CANLF WD repeat domain 72 OS=Canis lupus familiaris OX=9615 GN=WDR72 PE=4 SV=2	0,52	0,53	59,90	246479,0	1	1	0	0,75	1071	16644
>tr|E2R3V4|E2R3V4_CANLF Transmembrane 4 L six family member 1 OS=Canis lupus familiaris OX=9615 GN=TM4SF1 PE=3 SV=3	0,29	0,29	41,90	243827,9	1	1	0	16,24	117	14014
>tr|E2RCQ7|E2RCQ7_CANLF Serine peptidase inhibitor, Kunitz type 4 OS=Canis lupus familiaris OX=9615 GN=SPINT4 PE=4 SV=3	0,14	0,14	66,00	239976,0	1	1	0	10,20	98	11536
>sp|O18840|ACTB_CANLF Actin, cytoplasmic 1 OS=Canis lupus familiaris OX=9615 GN=ACTB PE=2 SV=3	0,78	0,78	137,70	220413,7	1	1	0	4,80	375	642
>tr|Q9XSV4|Q9XSV4_CANLF CE10 protein OS=Canis lupus familiaris OX=9615 GN=ce10 PE=2 SV=1	0,37	0,37	171,60	220323,8	1	1	0	10,00	110	41542
>tr|E2RCX8|E2RCX8_CANLF TEF transcription factor, PAR bZIP family member OS=Canis lupus familiaris OX=9615 GN=TEF PE=3 SV=3	0,52	0,52	50,80	209626,8	1	1	1	6,09	345	22800
>tr|F1PP51|F1PP51_CANLF Phosphatidylinositol specific phospholipase C X domain containing 2 OS=Canis lupus familiaris OX=9615 GN=PLCXD2 PE=4 SV=3	0,11	0,11	66,70	197989,2	1	1	1	3,29	304	17707
>tr|F1PN88|F1PN88_CANLF Dynein axonemal heavy chain 17 OS=Canis lupus familiaris OX=9615 GN=DNAH17 PE=3 SV=3	0,74	0,75	55,80	186978,3	1	1	0	0,20	4462	42143
>tr|F1Q2G6|F1Q2G6_CANLF Kelch like family member 10 OS=Canis lupus familiaris OX=9615 GN=KLHL10 PE=4 SV=2	0,80	0,80	63,00	161721,3	1	1	0	1,32	608	4666
>tr|A0A5F4C8I7|A0A5F4C8I7_CANLF Hydroxy-delta-5-steroid dehydrogenase, 3 beta- and steroid delta-isomerase 7 OS=Canis lupus familiaris OX=9615 GN=HSD3B7 PE=3 SV=1	0,10	0,08	41,80	158946,8	1	1	1	8,16	196	19417
>tr|A0A5F4BXR1|A0A5F4BXR1_CANLF Proton-translocating NAD(P)(+) transhydrogenase OS=Canis lupus familiaris OX=9615 GN=NNT PE=3 SV=1	0,76	0,76	111,40	152457,2	1	1	0	0,87	1029	17699
>tr|E2RCT1|E2RCT1_CANLF WAP domain-containing protein OS=Canis lupus familiaris OX=9615 PE=4 SV=2	1,36	1,36	131,70	97579,4	1	1	0	13,79	116	21717
>tr|F1Q0F4|F1Q0F4_CANLF Peptidylprolyl isomerase OS=Canis lupus familiaris OX=9615 GN=FKBP15 PE=4 SV=2	0,15	0,15	107,00	91910,4	1	1	1	0,91	1204	4607
>tr|A0A5F4C8Y0|A0A5F4C8Y0_CANLF Tensin 2 OS=Canis lupus familiaris OX=9615 GN=TNS2 PE=3 SV=1	0,13	0,13	65,00	84011,0	1	1	0	0,69	1306	12630
