# Supplementary material for: Proteome Profiling of Canine Epididymal Fluid: In Search of Protein Markers of Epididymal Sperm Motility
Source: Int J Mol Sci. 2023 Sep 30;24(19):14790. doi: 10.3390/ijms241914790 (PMC10573609; doi:10.3390/ijms241914790)
Supplement: Supplementary file 1 [file ijms-24-14790-s001.zip › Supplementary Table S3.pdf]

Supplementary Table S3. Age and cauda epididymal sperm count of individual dogs (*Canis lupus familiaris*) used in the study, divided into two groups according to epididymal sperm progressive motility: good sperm motility (GSM) and poor sperm motility (PSM).

Dog's number	Dog's age [months]	Dog's cauda epididymal sperm count [$\times 10^8$ spermatozoa/mL]
**GSM**		
1	18	10.9
2	36	5.6
5	12	17.9
6	24	12.0
10	36	16.4
11	24	14.3
13	42	7.2
14	72	15.5
15	72	33.8
21	72	11.2
23	120	8.4
24	108	17.1
25	132	16.5
Av.	59	14.4
**PSM**		
3	30	9.3
4	24	10.2
7	36	15.2
8	24	14.0
12	72	4.5
16	60	13.7
17	60	15.8
22	84	8.6
26	120	16.6
28	84	11.7
Av.	59	12.0
